# Assigning the Absolute Configurations of Chiral Primary Amines Based on Experimental and DFT-Calculated ^19^F Nuclear Magnetic Resonance

**DOI:** 10.3389/fchem.2019.00318

**Published:** 2019-05-20

**Authors:** Shiwei Yang, Guangling Bian, Rongjian Sa, Ling Song

**Affiliations:** ^1^The Key Laboratory of Coal to Ethylene Glycol and Its Related Technology, Fujian Institute of Research on the Structure of Matter, Chinese Academy of Sciences, Fuzhou, China; ^2^University of Chinese Academy of Sciences, Beijing, China; ^3^Institute of Oceanography, Ocean College, Fujian Provincial Key Laboratory of Information Processing and Intelligent Control, Minjiang University, Fuzhou, China

**Keywords:** absolute configuration assignment, primary amine, ^19^F nuclear magnetic resonance, DFT calculation, fluorinated phenylacetic phenylselenoester

## Abstract

In this work, a novel method for assigning the absolute configuration of a chiral primary amine has been developed based on the experimental and DFT-calculated ^19^F NMR chemical shift differences of its derived two fluorinated amides by reacting with two enantiomers of a chiral derivatizing agent FPP (α-fluorinated phenylacetic phenylselenoester) separately. Comparing the experimental chemical shift difference $\Delta \delta_{\alpha -F}^{R,S}$ of (R)-FPA-amide/(S)-FPA-amide with the calculated Δδ_α-F^*R,S*^_ of (R)-FPA-(R)-amide/(S)-FPA-(R)-amide, if the experimental Δδ_α-F^*R,S*^_ has the same symbol (positive or negative) as one of the theoretical Δδ_α-F^*R,S*^_, the assigned configuration of the amine is considered to be consistent with the theoretical one. Our method could be applied to a broad substrate scope avoiding wrong conclusion due to empirical judgment.

## Introduction

Over the decade, the increased tremendous demand of optically pure organic compounds in many fields, such as drug discovery, medicinal chemistry, and asymmetric synthesis, has promoted the exploration of practical strategies for determining the absolute configuration of a chiral molecule (Bijvoet et al., 1951; Flack and Bernardinelli, 2000; De Gussem et al., 2012; Burtea and Rychnovsky, 2017; Liu et al., 2017; Sairenji et al., 2017; Yan et al., 2017; Ma et al., 2018). Among varied developed technologies for this purpose, NMR spectroscopic detection of the chemical shift differences of NMR signals of formed diastereomers of chiral guests with chiral agents is one of the most used methods, which is simple and convenient, giving straightforward information for analysis without the need of standard samples (Shvo et al., 1967; Jacobus et al., 1968; Ohtani et al., 1991; Takeuchi et al., 1991, 1993, 2004, 2006; Hanna and Lau-Cam, 1993; Trost et al., 1994; Hoye and Renner, 1996; Kirk, 1998; Yabuuchi and Kusumi, 2000; Fujiwara et al., 2001; Seco et al., 2004; Freire et al., 2005, 2008; Orlov and Ananikov, 2010, 2011; Wenzel and Chisholm, 2011; Kumari et al., 2013, 2015; Pal et al., 2014; Bian et al., 2015; Lakshmipriya et al., 2016; Laskowski et al., 2016; Yan et al., 2017; Burns et al., 2018).

**Figure 1:**
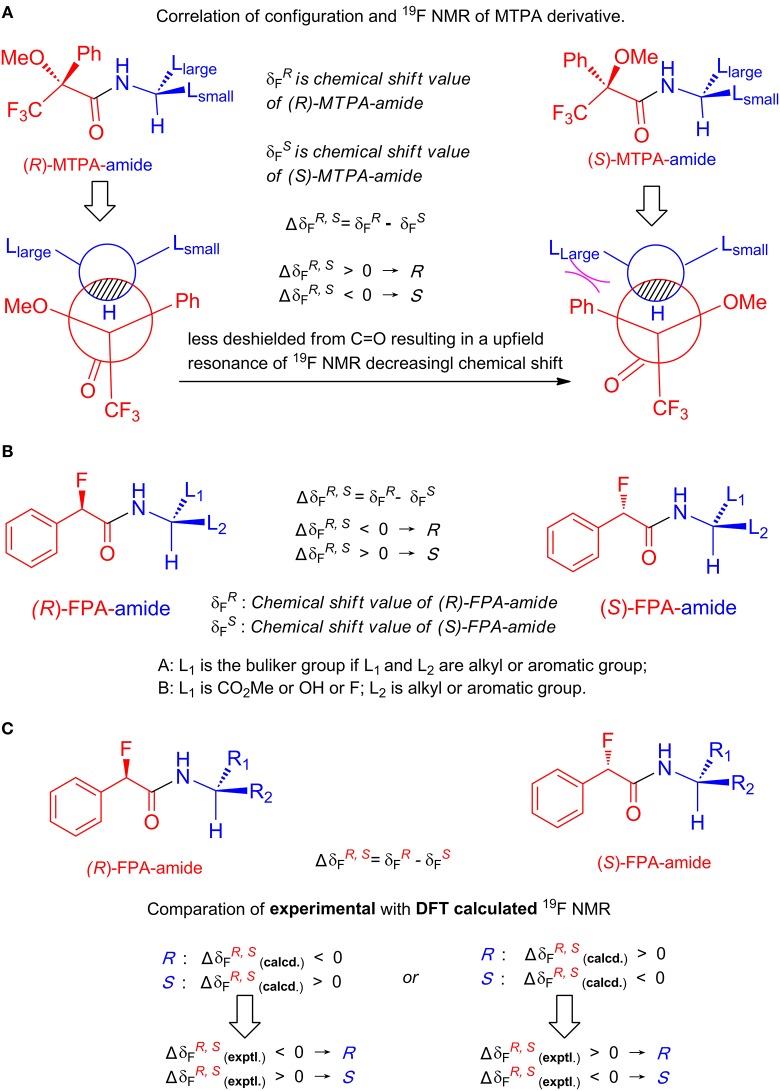
**(A)** Mosher's models. **(B)** Hamman's models. **(C)** Our method.

Chiral amines have been ubiquitous in natural products and widely used in the field of medicine, so it is very important to determine their absolute configurations. Up to now, the published research concerning the determination of absolute configuration by NMR is mostly based on ^1^H NMR. It is very rare to use ^19^F NMR for this purpose and all are based on empirical models. Determining the absolute configuration of amines in ^19^F NMR was firstly reported by Mosher using triflurophenyl acetic acid as the chiral derivatizing agent (CDA) who empirically assigned the absolute configurations by comparing the ^19^F NMR experimental chemical shift differences of the formed amides between a chiral amine and two enantiomers of a CDA based on Mosher's models (Dale and Mosher, 1973; Sullivan et al., 1973) as shown in Figure 1A in which L_large_ is the large group of amine and L_small_ is the small group of amine by comparing their inherent stereochemistry. Successful assignment heavily depends on correct judgment of inherent stereochemistry of the groups. However, it is difficult to judge the intrinsic size of groups in many cases, which could result in wrong conclusions. In the 1990s, using alpha-fluorinated phenyl acetic acid (FPA) as a CDA, Hamman observed that correctly constructing the correlation between the absolute configuration of amines and chemical shifts of ^19^F NMR of formed alpha-fluorinated phenyl acetic amide depends on the property of the L_1_ and L_2_ groups of amines. If both groups are alkyl or aryl groups, L_1_ should be the bulkier group. However, if the formed amide having a CO_2_Me group attached to the chiral alpha carbon, then the L_1_ group is always a CO_2_Me group, no matter how large the other group is (Figure 1B) (Hamman, 1990, 1993; Temperini et al., 2017). In addition to the CO_2_Me group, our group also observed that if another functional group is attached (such as a hydroxyl group or fluorine atom), this group should be the L_1_ group in order to give correct configurations (Figure 1B). The interactions between the alpha chiral F atom and the functional groups may cause the judgment complexity of the L_1_ group. These observations indicate that proper judgment of the L_1_ group is critical for the correct assignment of absolute configuration. Thus, an empirical model based on inherent stereochemistry simply could not be established for correlating the absolute configurations of amines containing functional groups with the chemical shift values of their corresponding alpha-fluorinated phenyl acetic amides. Interestingly, the international union of pure and applied chemistry (IUPAC) rule for the assignment of the L_1_ group is more appropriate than inherent stereochemistry with the fluorinated phenyl acetic acid derived amides, but such kind of assignment is arbitrary. How does one establish the correlation between the absolute configuration of varied amines and ^19^F NMR signal of their corresponding alpha-fluorinated phenyl acetic amides excluding arbitrary assignment? Considering that the assignment of absolute configuration of chiral molecules using a circular dichroism spectrometer by comparing the experimental and calculated CD spectra has been well-established (Dickins et al., 1999; Aamouche et al., 2000; Huang et al., 2000; Pescitelli and Bruhn, 2016), we wonder if we can do the same thing with NMR.

Here, we report a novel method for the assignment of absolute configuration of a chiral amine by comparing the experimental and DFT-calculated ^19^F NMR chemical shift differences of its corresponding (*R*)-FPA-amide and (*S*)-FPA-amide derived from the chiral amine by reacting with two enantiomers of a CDA FPP (α-fluorinated phenylacetic phenylselenoester, Table 1) separately. By comparison, the experimental Δδ_α-F^*R,S*^_ (Δδ_α-F^R,S^_ = Δδ_α-F^*R*^_ – Δδ_α-F^*S*^_, where Δδ_α-F^*R*^_ and Δδ_α-F^*S*^_ are ^19^F-{1H} NMR values of (*R*)-FPA-amide and (*S*)-FPA-amide, respectively) has the same symbol (positive or negative) as one of the theoretical Δδ_α-F^*R,S*^_; the assigned configuration of the amine is considered to be consistent with the theoretical one (Figure 1C). The advantages of FPP are as follows: it can react with amines directly in NMR tubes to form amides immediately without the addition of other chemical reagents and give very clean solution avoiding any further handling step. Chiral FPP is stable in varied solvents over weeks and can be stored in a sealed bottle covered with foil for months in a refrigerator.

**Table 1:** Constructing the correlation between the absolute configuration of amines with the experimental measurements and DFT calculations of Δδ_α-F^*R,S*^_ of their corresponding (*R*)-FPA-amide and (*S*)-FPA-amide^a^.

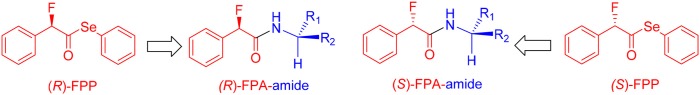									
**Entry**	**Amine**	**Contrasting spectra ^b^**	**Δδ_α-F^*R,S*^_(ppm)**		**Entry**	**Amine**	**Contrasting spectra ^b^**	**Δδ_α-F^*R,S*^_ (ppm)**	
			**Exptl. ^c^**	**Calcd**.				**Exptl. ^c^**	**Calcd**.
1	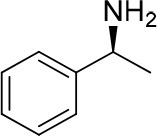	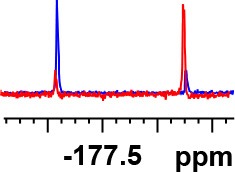	1.18 (*S*)	0.92 (*S*)	13	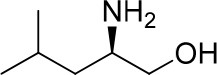	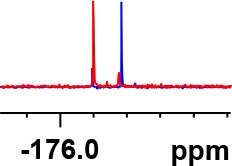	−0.30 (*R*)	−0.66 (*R*)
2	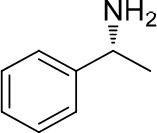	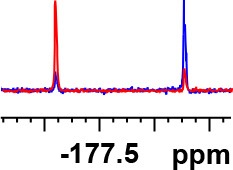	−1.19 (*R*)	−0.92 (*R*)	14	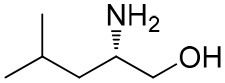	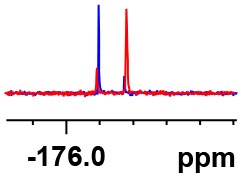	0.30 (*S*)	0.66 (*S*)
3	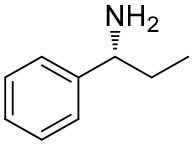	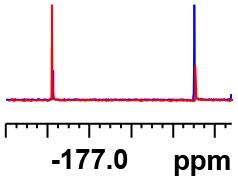	−1.73 (*R*)	−0.30 (*R*)	15	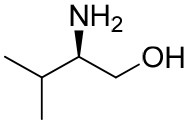	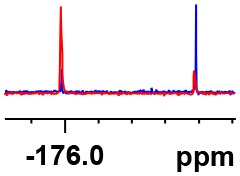	−1.34 (*R*)	−0.89
4	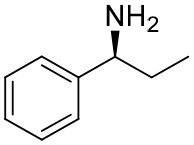	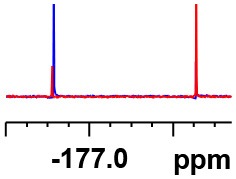	1.65 (*S*)	0.30 (*S*)	16	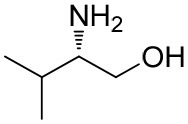	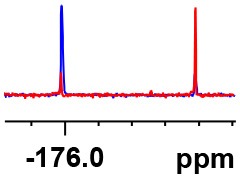	1.34 (*S*)	0.89
5	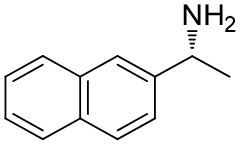	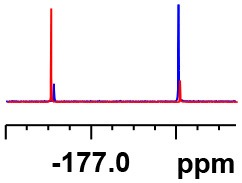	−1.50 (*R*)	−1.35 (*R*)	17	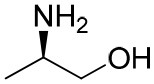	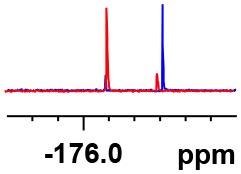	−0.68 (*R*)	−1.26 (*R*)
6	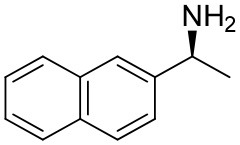	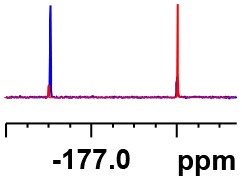	1.51 (*S*)	1.35 (*S*)	18	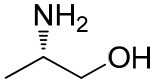	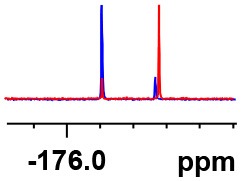	0.68 (*S*)	1.26 (*S*)
7	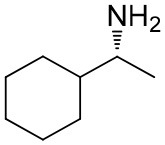	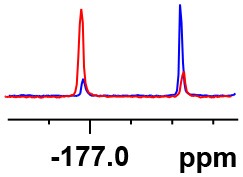	−0.23 (*R*)	−0.58 (*R*)	19	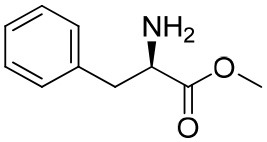	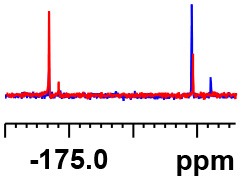	−2.23 (*R*)	−0.77 (*R*)
8	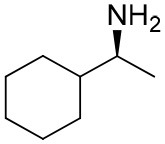	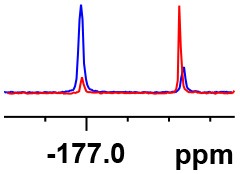	0.23 (*S*)	0.58 (*S*)	20	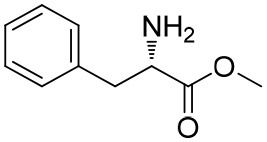	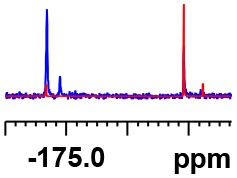	2.25 (*S*)	0.77 (*S*)
9	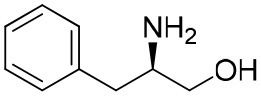	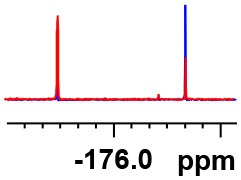	−2.33 (*R*)	−3.23 (*R*)	21	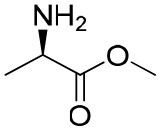	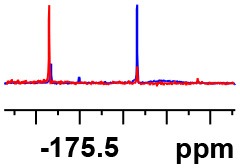	−1.01 (*R*)	−0.50
10	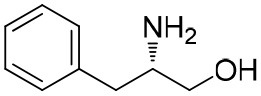	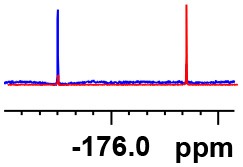	2.33 (*S*)	3.23 (*S*)	22	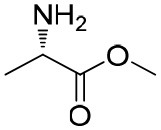	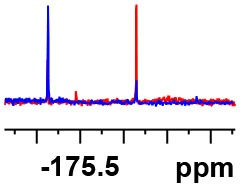	1.01 (*S*)	0.50
11	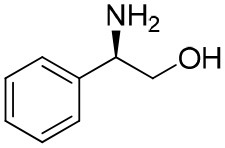	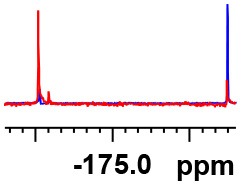	−2.44 (*R*)	−1.55 (*R*)	23	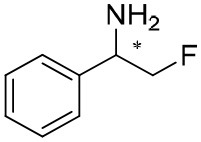	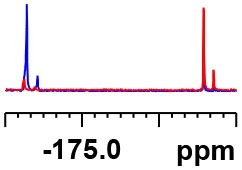	2.29 (*S*)^d^	−0.68 (*R*)
12	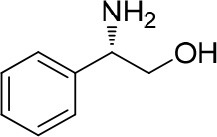	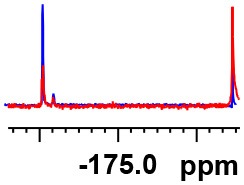	2.44 (*S*)	1.55 (*S*)					0.68 (*S*)

a (R)-FPP or (S)-FPP (0.017 mmol) and amine (0.085 mmol) in 0.5 ml CDCl_3_, the proton-decoupled ^19^F NMR spectra were collected on a Bruker Avance 400-MHz spectrometer at 25°C (the solvent was DMSO-d_6_ in entries 17–20), ^19^F NMR experiment (15-s delay time, 64 scans each) and trifluorotoluene as internal standard (−63.9 ppm).

b The blue spectra were obtained from (R)-FPA-amide and the red spectra were obtained from (S)-FPA-amide.

c The assigned configurations were labeled in parentheses.

d *Assigned configuration was confirmed by comparison the optical rotation data with reference (Thvedt et al., 2010)*.

## Materials and Methods

### General Information and Materials

All commercial reagents were used as received without further purification unless otherwise stated. All reactions were run under N_2_ unless otherwise indicated. NMR spectra were recorded using a 400-MHz spectrometer. Chemical shifts were reported in parts per million (ppm), using CDCl_3_ (δ_H_ = 7.26 ppm, δ_C_ = 77.16 ppm) and trifluorotoluene (−63.9 ppm) as internal standards. Multiplicities are indicated as s (singlet), d (doublet), t (triplet), q (quartet), and m (multiplet). High-resolution mass spectra (HRMS) were obtained by the ESI ionization sources using the TOF MS technique. The calculated ^19^F NMR shifts were referenced to trifluorotoluene (σ_ref_ = 257.58 ppm, δ_ref_ = −63.9 ppm).

### General Synthesis Procedure for Chiral FPP

**Scheme 1:**
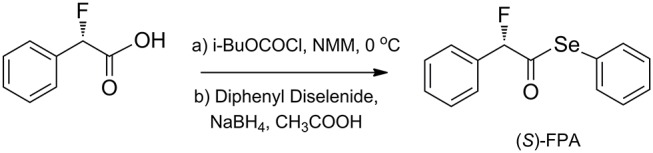
General synthesis procedure for chiral FPP.

Chiral FPP was readily prepared by a one-pot procedure reported by Temperini's group (Temperini et al., 2017) using chiral α-fluorinated phenylacetic acid and diphenyl diselenide as the starting materials (the procedure is illustrated in Scheme 1).

To a mixture of (*S*)-α-fluorobenzeneacetic acid (1.0 g, 6.49 mmol) and *N*-methylmorpholine (NMM) (722 mg, 7.14 mmol) in 20 ml of dried ethyl acetate, *i*-BuOCOCl (975 mg, 7.14 mmol) was added dropwise in 30 min under N_2_ at 0°C. After addition, the mixture was stirred for another 30 min at 0°C. Then, a fresh and white solution of nucleophilic selenium species, prepared by reacting diphenyl diselenide (1.48 g, 3.245 mmol), sodium borohydride (245 mg, 6.49 mmol), and CH_3_COOH (973.5 mg, 16.225 mmol) in 10 ml of dried ethyl acetate at 40°C for 30 min, was added to the above mixture, and the stirring was continued at room temperature until completion of reaction as detected by TLC. The mixture was quenched by 5 ml of 1 M HCl and extracted by ethyl acetate (20 ml × 2). The organic phase was washed by brine (20 ml), dried over anhydrous sodium sulfate, and concentrated *in vacuo*. The crude product was purified *via* flash chromatography on silica gel (petroleum ether/ethyl acetate 10:1 to 5:1), affording the corresponding product (S)-FPP (little yellow oil, total yield up to 69%) (Supplementary Figures S1–S4). [α]${}_{\text{D}}^{22}$ = 13.4° (c = 1.0 in CHCl_3_); ^1^H NMR (400 MHz, CDCl_3_) δ 7.54–7.47 (m, 4H), 7.46–7.43 (m, 2H), 7.42–7.37 (m, 4H), 5.81 (d, *J* = 47.6 Hz, 1H) ppm; ^13^C NMR (CDCl_3_, 100 MHz) δ 198.8, 136.2, 135.8, 132.7, 130.1, 129.5, 129.4, 129.0, 128.9, 128.8, 127.9, 126.7, 95.7 (d, *J* = 189 Hz) ppm; ^19^F-{1H} NMR (376 MHz, CDCl_3_) δ −180.8 ppm; HRMS (ESI) Calcd for [C_14_H_11_FOSeNa, M+Na]^+^: 316.9851, found: 316.9849.

Following the same procedure, (*R*)-FPP was synthesized from (*R*)-α-fluorobenzeneacetic acid (little yellow oil, total yield up to 67%). [α]${}_{\text{D}}^{22}$ = −13.2° (c = 1.0 in CHCl_3_).

### Configurational Assignment of Amines by FPP

FPP [0.017 mmol, (*R*)-FPP or (*S*)-FPP] and amine (0.085 mmol) were mixed in 0.5 ml of CDCl_3_, and the proton-decoupled ^19^F NMR spectra were collected on a Bruker Avance 400-MHz spectrometer at 25°C (the solvent was DMSO-d_6_ for guests 17–20) (delay time: 15 s, 64 scans each for ^19^F-{1H} NMR experiment). The internal standard of fluorine spectrum is 4-trifluorotoluene (−63.9 ppm).

## Results and Discussion

First, to examine FPP as in-tube CDA for chiral amines, 1 equiv. of (*S*)-FPP was added to an NMR tube with 1 equiv. of (*S*)-α-phenylethanamine in CDCl_3_ under air at room temperature giving clean solution, and the proton-decoupled ^19^F(^19^F-{1H}) NMR spectra were recorded immediately. The corresponding amide was generated within 5 min and the whole derivatizing process was finished within 20 min. Also, the fluorine chemical shift values of the derivatives do not vary with the reaction time and the different ratios of chiral FPP/amine (Supplementary 1), which is crucial to determine the absolute configuration. It is unfortunate that slightly partial racemization happened during the derivatization, which leads to the failure of measuring the enantiomeric purity of chiral amines, but it does not affect the assignment of absolute configuration by using the main enantiomers' signals.

Then, we recorded the ^19^F-{1H} NMR spectra of a series of amines with known configurations after FPP derivatization (Supplementary Figures S5–S50). The results are listed in Table 1 (entries 1–22). It can be seen that the obtained Δδ_α-F^*R,S*^_ values are positive for all tested (*S*)-amines and are negative for all tested (*R*)-amines, whether the amine is aromatic amine, fatty amine, amino alcohol, or amino acid ester.

How to correlate configurations with Δδ_α-F^*R,S*^_ reasonably is the key to establishing the method of determining absolute configuration by ^19^F NMR with FPP. For the amines of entries 1–8, correct configurations could be given by comparing the inherent stereochemistry of groups based on Hamman's model. However, for several amines (Table 1, entries 9–16, 19, and 20), the comparison of the inherent stereochemistry of groups based on Hamman's model gave incorrect assignment of the absolute configuration, which is likely to be caused by the electron effect of the heteroatom (Hamman, 1990, 1993; Apparu et al., 2000). Thus, we need to explore a new strategy to overcome these problems. We propose that δ_α−F_ in FPA-amide is mainly influenced by the composite factors of electronic and steric hindrance effects of groups. It is unrealistic to correlate interactions of electronic and steric hindrance effects by a simple empirical model. Nevertheless, in today's highly developed computational chemistry, the combined effects of electronic effect and steric hindrance can be achieved by strict theoretical calculation. By theoretically calculating the chemical shift differences of the amide formed from a chiral amine with two enantiomers of FPP separately and comparing the experiment and calculated values, a new method for assigning the absolute configuration could be well-established.

Our theoretical calculation is as follows. Conformation screening was applied using molecular mechanics methods in our calculations in order to obtain more reliable results. Nine energy favorable conformations of every molecule were selected and geometry optimization was performed based on B3LYP/6-111 G(d,p) level. Vibrational frequency analyses at the same basis sets were used on all optimized structures in order to characterize stationary points as local minima. Then, the Gibbs free energy with zero-point energy (ZPE) corrections was obtained for every conformer. The lowest Gibbs free energy conformers were selected and the ^19^F NMR parameters for FPA-amines were calculated at the level of B972/cc-PVQZ with the GIAO method. All the calculations were in chloroform solvent and the solvent effects were evaluated by the IEFPCM model. The Gaussian 09 package (Frisch et al., 2010) was used for all of our calculations. All the conformers were local minimum and verified by frequency calculation. Also, two molecules were selected to perform ^19^F NMR calculation for all conformers according to Boltzmann equations based on free energy data by frequency calculation. It was found that there is a trivial difference in Δδ_α-F^*R,S*^_ between the data of the most stable conformer and the statistic values (Supplementary 3). Hence, the most stable conformer of each molecule was used to calculate ^19^F NMR and Δδ_α-F^*R,S*^_ data. The calculated ^19^F chemical shifts for the lowest-energy conformers were in agreement with the experimental values (Table 1, entries 1–22). Then, the method is applied to determine the absolute configuration of an amine with unknown configuration (2-fluoro-1-phenylethanamine in Table 1, entry 23). The calculation-predicted Δδ_α-F^*R,S*^_ of (*R*)-amide of amine 23 was negative (−0.68), and Δδ_α-F^*R,S*^_ of (*S*)-amide of amine 23 was positive (0.68). The experimental measured Δδ_α-F^*R,S*^_ of the derived amide from amine 23 is positive (2.29), so the assigned configuration of amine 23 was *S*, which was confirmed by comparison of the optical rotation data with reference (Thvedt et al., 2010) (Supplementary 2). In addition, it should be noted that the present method was limited to be chiral amines, including a chiral tertiary carbon center and sterically more differentiated groups (L_1_/L_2_). As to chiral amines containing similar size in space, the obtained chemical shift difference is too small to be suitable for judging absolute configurations (Hamman, 1990).

## Conclusions

A novel method for assigning the absolute configuration of chiral primary amines based on experimental and calculated ^19^F NMR has been developed. The method employs a new type of CDA, chiral α-fluorinated phenylacetic phenylselenoester, which can derivatize a primary amine directly in an NMR tube. Calculating the Δδ_α-F^*R,S*^_ value of (*R*)-FPA-(*R*)-amide and (*S*)-FPA-(*R*)-amide and the Δδ_α-F^*R,S*^_ value of (*R*)-FPA-(*S*)-amide and (*S*)-FPA-(*S*)-amide, the experimental chemical shift difference Δδ_α-F^*R,S*^_ of (*R*)-FPA-amide and (*S*)-FPA-amide is compared with the calculated values. If the experimental Δδ_α-F^*R,S*^_ has the same symbol (positive or negative) as one of the theoretical Δδ_α-F^*R,S*^_, the assigned configuration of the amine is considered to be consistent with the theoretical one. Since both electronic and steric hindrance effects for the absolute configuration are considered, our method is widely valid for a broad substrate scope and avoids incorrect assignment of absolute configuration by empirical judgment.

## Data Availability

All datasets generated for this study are included in the manuscript and the Supplementary Files.

## Author Contributions

All authors listed have made a substantial, direct and intellectual contribution to the work, and approved it for publication.

### Conflict of Interest Statement

The authors declare that the research was conducted in the absence of any commercial or financial relationships that could be construed as a potential conflict of interest.
