# Supplementary material for: Assigning the Absolute Configurations of Chiral Primary Amines Based on Experimental and DFT-Calculated 19F Nuclear Magnetic Resonance
Source: Front Chem. 2019 May 20;7:318. doi: 10.3389/fchem.2019.00318 (PMC6536038; doi:10.3389/fchem.2019.00318)
Supplement: Supplementary file 1 [file Table_1.docx]

Supplementary Information

**Assigning the Absolute Configurations of Chiral Primary Amines Based on Experimental and DFT Calculated ^19^F NMR**

Shiwei Yang^1,2^, Guangling Bian^1,2*^, Rongjian Sa^3*^, Ling Song^1,2*^

^1^University of Chinese Academy of Sciences, Beijing, P. R. China.

^2^The Key Laboratory of Coal to Ethylene Glycol and Its Related Technology, Fujian Institute of Research on the Structure of Matter, Chinese Academy of Sciences, Fuzhou, P. R. China.

^3^Institute of Oceanography, Ocean college, Fujian Provincial Key Laboratory of Information Processing and Intelligent Control, Minjiang University, Fuzhou, P. R. China.

*** Correspondence:**Dr. Bian Guangling, [glb@fjirsm.ac.cn](mailto:glb@fjirsm.ac.cn);

Dr. Sa Rongjian, [rjsa@mju.edu.cn](mailto:rjsa@mju.edu.cn);

Dr. Song Ling, [songling@fjirsm.ac.cn](mailto:songling@fjirsm.ac.cn).

**Table of Contents**

[1. Effects of reaction time and reagent ratio on the derivatization process. 4](#_Toc6653870)

[Figure S1. ^1^H NMR of (*S*)-FPP (400 MHz, TMS) 4](#_Toc6653871)

[Figure S2. ^13^C NMR of (*S*)-FPP (100 MHz, CDCl_3_) 5](#_Toc6653872)

[Figure S3. ^19^ F-{1H} NMR of (*S*)-FPP (376 MHz, Trifluorotoluene) 5](#_Toc6653873)

[Figure S4. HRMS of (*S*)-FPP 6](#_Toc6653874)

[Figure S5. ^19^F-{1H} NMR of (*R*)-FPA-amide 1 6](#_Toc6653875)

[Figure S6. ^19^F-{1H} NMR of (*S*)-FPA-amide 1 7](#_Toc6653876)

[Figure S7. ^19^F-{1H} NMR of (*R*)-FPA-amide 2 7](#_Toc6653877)

[Figure S8. ^19^F-{1H} NMR of (*S*)-FPA-amide 2 8](#_Toc6653878)

[Figure S9. ^19^F-{1H} NMR of (*R*)-FPA-amide 3 8](#_Toc6653879)

[Figure S10. ^19^F-{1H} NMR of (*S*)-FPA-amide 3 9](#_Toc6653880)

[Figure S11. ^19^F-{1H} NMR of (*R*)-FPA-amide 4 9](#_Toc6653881)

[Figure S12. ^19^F-{1H} NMR of (*S*)-FPA-amide 4 10](#_Toc6653882)

[Figure S13. ^19^F-{1H} NMR of (*R*)-FPA-amide 5 10](#_Toc6653883)

[Figure S14. ^19^F-{1H} NMR of (*S*)-FPA-amide 5 11](#_Toc6653884)

[Figure S15. ^19^F-{1H} NMR of (*R*)-FPA-amide 6 11](#_Toc6653885)

[Figure S16. ^19^F-{1H} NMR of (*S*)-FPA-amide 6 12](#_Toc6653886)

[Figure S17. ^19^F-{1H} NMR of (*R*)-FPA-amide 7 12](#_Toc6653887)

[Figure S18. ^19^F-{1H} NMR of (*S*)-FPA-amide 7 13](#_Toc6653888)

[Figure S19. ^19^F-{1H} NMR of (*R*)-FPA-amide 8 13](#_Toc6653889)

[Figure S20. ^19^F-{1H} NMR of (*S*)-FPA-amide 8 14](#_Toc6653890)

[Figure S21. ^19^F-{1H} NMR of (*R*)-FPA-amide 9 14](#_Toc6653891)

[Figure S22. ^19^F-{1H} NMR of (*S*)-FPA-amide 9 15](#_Toc6653892)

[Figure S23. ^19^F-{1H} NMR of (*R*)-FPA-amide 10 15](#_Toc6653893)

[Figure S24. ^19^F-{1H} NMR of (*S*)-FPA-amide 10 16](#_Toc6653894)

[Figure S25. ^19^F-{1H} NMR of (*R*)-FPA-amide 11 16](#_Toc6653895)

[Figure S26. ^19^F-{1H} NMR of (*S*)-FPA-amide 11 17](#_Toc6653896)

[Figure S27. ^19^F-{1H} NMR of (*R*)-FPA-amide 12 17](#_Toc6653897)

[Figure S28. ^19^F-{1H} NMR of (*S*)-FPA-amide 12 18](#_Toc6653898)

[Figure S29. ^19^F-{1H} NMR of (*R*)-FPA-amide 13 18](#_Toc6653899)

[Figure S30. ^19^F-{1H} NMR of (*S*)-FPA-amide 13 19](#_Toc6653900)

[Figure S31. ^19^F-{1H} NMR of (*R*)-FPA-amide 14 19](#_Toc6653901)

[Figure S32. ^19^F-{1H} NMR of (*S*)-FPA-amide 14 20](#_Toc6653902)

[Figure S33. ^19^F-{1H} NMR of (*R*)-FPA-amide 15 20](#_Toc6653903)

[Figure S34. ^19^F-{1H} NMR of (*S*)-FPA-amide 15 21](#_Toc6653904)

[Figure S35. ^19^F-{1H} NMR of (*R*)-FPA-amide 16 21](#_Toc6653905)

[Figure S36. ^19^F-{1H} NMR of (*S*)-FPA-amide 16 22](#_Toc6653906)

[Figure S37. ^19^F-{1H} NMR of (*R*)-FPA-amide 17 22](#_Toc6653907)

[Figure S38. ^19^F-{1H} NMR of (*S*)-FPA-amide 17 23](#_Toc6653908)

[Figure S39. ^19^F-{1H} NMR of (*R*)-FPA-amide 18 23](#_Toc6653909)

[Figure S40. ^19^F-{1H} NMR of (*S*)-FPA-amide 18 24](#_Toc6653910)

[Figure S41. ^19^F-{1H} NMR of (*R*)-FPA-amide 19 24](#_Toc6653911)

[Figure S42. ^19^F-{1H} NMR of (*S*)-FPA-amide 19 25](#_Toc6653912)

[Figure S43. ^19^F-{1H} NMR of (*R*)-FPA-amide 20 25](#_Toc6653913)

[Figure S44. ^19^F-{1H} NMR of (*S*)-FPA-amide 20 26](#_Toc6653914)

[Figure S45. ^19^F-{1H} NMR of (*R*)-FPA-amide 21 26](#_Toc6653915)

[Figure S46. ^19^F-{1H} NMR of (*S*)-FPA-amide 21 27](#_Toc6653916)

[Figure S47. ^19^F-{1H} NMR of (*R*)-FPA-amide 22 27](#_Toc6653917)

[Figure S48. ^19^F-{1H} NMR of (*S*)-FPA-amide 22 28](#_Toc6653918)

[Figure S49. ^19^F-{1H} NMR of (*R*)-FPA-amide 23 28](#_Toc6653919)

[Figure S50. ^19^F-{1H} NMR of (*S*)-FPA-amide 23 29](#_Toc6653920)

[2. Computational models of complexes. 29](#_Toc6653921)

[3. ^19^F NMR calculation for all conformers according to Boltzmann equations based on free energies data by frequency calculation. 83](#_Toc6653922)

1. Effects of reaction time and reagent ratio on the derivatization process.

**A**: (*S*)-FPP

**B**: Amine **1** + 1.0 eqviv. (*S*)-FPP, 20 mins

**C**: Amine **1** + 0.5 eqviv. (*S*)-FPP, 20 mins

**D**: Amine **1** + 0.2 eqviv. (*S*)-FPP, 20 mins

**E**: Amine **1** + 0.2 eqviv. (*S*)-FPP, 5 mins

**F**: Amine **1** + 0.2 eqviv. (*S*)-FPP, 12 hours

# Figure S1. ^1^H NMR of (*S*)-FPP (400 MHz, TMS)

# Figure S2. ^13^C NMR of (*S*)-FPP (100 MHz, CDCl_3_)

# Figure S3. ^19^ F-{1H} NMR of (*S*)-FPP (376 MHz, Trifluorotoluene)

# Figure S4. HRMS of (*S*)-FPP


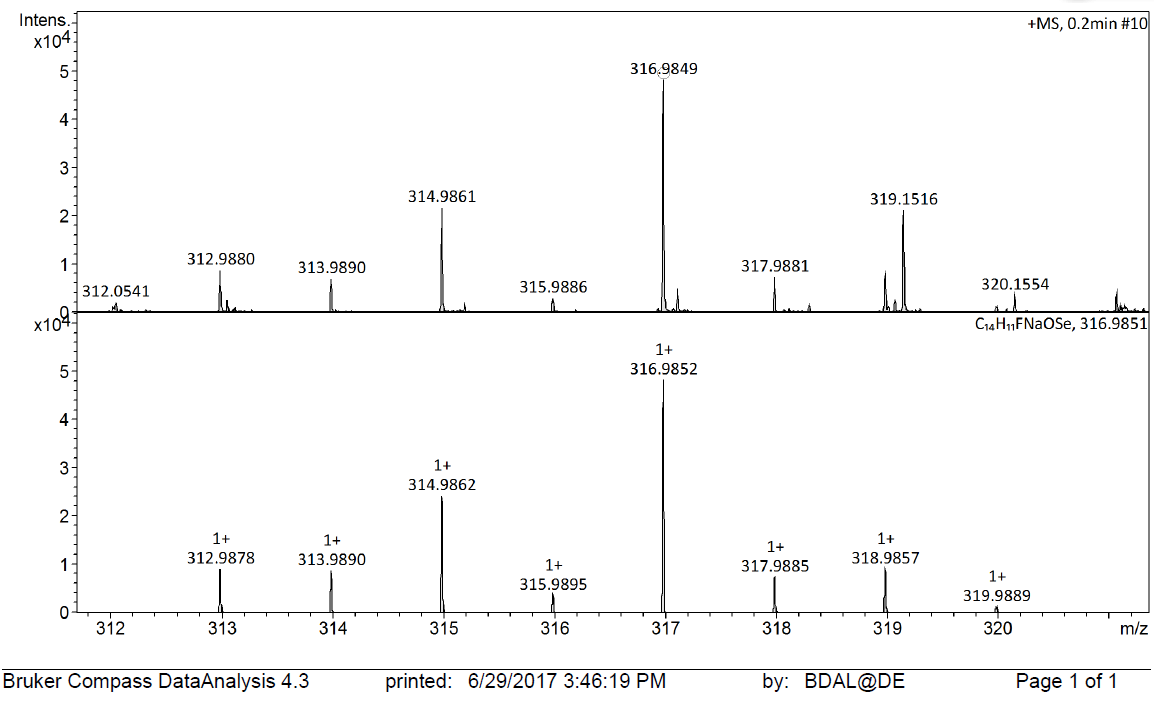

HRMS (ESI) Calcd For [C_14_H_11_FNaOSe, M+Na] ^+^: 316.9851, found: 316.9849.

# Figure S5. ^19^F-{1H} NMR of (*R*)-FPA-amide 1

# Figure S6. ^19^F-{1H} NMR of (*S*)-FPA-amide 1

# Figure S7. ^19^F-{1H} NMR of (*R*)-FPA-amide 2

# Figure S8. ^19^F-{1H} NMR of (*S*)-FPA-amide 2

# Figure S9. ^19^F-{1H} NMR of (*R*)-FPA-amide 3

# Figure S10. ^19^F-{1H} NMR of (*S*)-FPA-amide 3

# Figure S11. ^19^F-{1H} NMR of (*R*)-FPA-amide 4

# Figure S12. ^19^F-{1H} NMR of (*S*)-FPA-amide 4

# Figure S13. ^19^F-{1H} NMR of (*R*)-FPA-amide 5

# Figure S14. ^19^F-{1H} NMR of (*S*)-FPA-amide 5

# Figure S15. ^19^F-{1H} NMR of (*R*)-FPA-amide 6

# Figure S16. ^19^F-{1H} NMR of (*S*)-FPA-amide 6

# Figure S17. ^19^F-{1H} NMR of (*R*)-FPA-amide 7

# Figure S18. ^19^F-{1H} NMR of (*S*)-FPA-amide 7

# Figure S19. ^19^F-{1H} NMR of (*R*)-FPA-amide 8

# Figure S20. ^19^F-{1H} NMR of (*S*)-FPA-amide 8

# Figure S21. ^19^F-{1H} NMR of (*R*)-FPA-amide 9

# Figure S22. ^19^F-{1H} NMR of (*S*)-FPA-amide 9

# Figure S23. ^19^F-{1H} NMR of (*R*)-FPA-amide 10

# Figure S24. ^19^F-{1H} NMR of (*S*)-FPA-amide 10

# Figure S25. ^19^F-{1H} NMR of (*R*)-FPA-amide 11

# Figure S26. ^19^F-{1H} NMR of (*S*)-FPA-amide 11

# Figure S27. ^19^F-{1H} NMR of (*R*)-FPA-amide 12

# Figure S28. ^19^F-{1H} NMR of (*S*)-FPA-amide 12

# Figure S29. ^19^F-{1H} NMR of (*R*)-FPA-amide 13

# Figure S30. ^19^F-{1H} NMR of (*S*)-FPA-amide 13

# Figure S31. ^19^F-{1H} NMR of (*R*)-FPA-amide 14

# Figure S32. ^19^F-{1H} NMR of (*S*)-FPA-amide 14

# Figure S33. ^19^F-{1H} NMR of (*R*)-FPA-amide 15

# Figure S34. ^19^F-{1H} NMR of (*S*)-FPA-amide 15

# Figure S35. ^19^F-{1H} NMR of (*R*)-FPA-amide 16

# Figure S36. ^19^F-{1H} NMR of (*S*)-FPA-amide 16

# Figure S37. ^19^F-{1H} NMR of (*R*)-FPA-amide 17

# Figure S38. ^19^F-{1H} NMR of (*S*)-FPA-amide 17

# Figure S39. ^19^F-{1H} NMR of (*R*)-FPA-amide 18

# Figure S40. ^19^F-{1H} NMR of (*S*)-FPA-amide 18

# Figure S41. ^19^F-{1H} NMR of (*R*)-FPA-amide 19

# Figure S42. ^19^F-{1H} NMR of (*S*)-FPA-amide 19

# Figure S43. ^19^F-{1H} NMR of (*R*)-FPA-amide 20

# Figure S44. ^19^F-{1H} NMR of (*S*)-FPA-amide 20

# Figure S45. ^19^F-{1H} NMR of (*R*)-FPA-amide 21

# Figure S46. ^19^F-{1H} NMR of (*S*)-FPA-amide 21

# Figure S47. ^19^F-{1H} NMR of (*R*)-FPA-amide 22

# Figure S48. ^19^F-{1H} NMR of (*S*)-FPA-amide 22

# Figure S49. ^19^F-{1H} NMR of (*R*)-FPA-amide 23

# Figure S50. ^19^F-{1H} NMR of (*S*)-FPA-amide 23

# 2. Computational models of complexes.

**Kelly spheres, F atoms; Blue spheres, N atoms; red spheres, O atoms; gray spheres, C atoms; and white spheres, H atoms.**

**Ball-cylinder model for (*R*)-FPA-amide 1 and cartesian coordinates (Gaussian 09).**


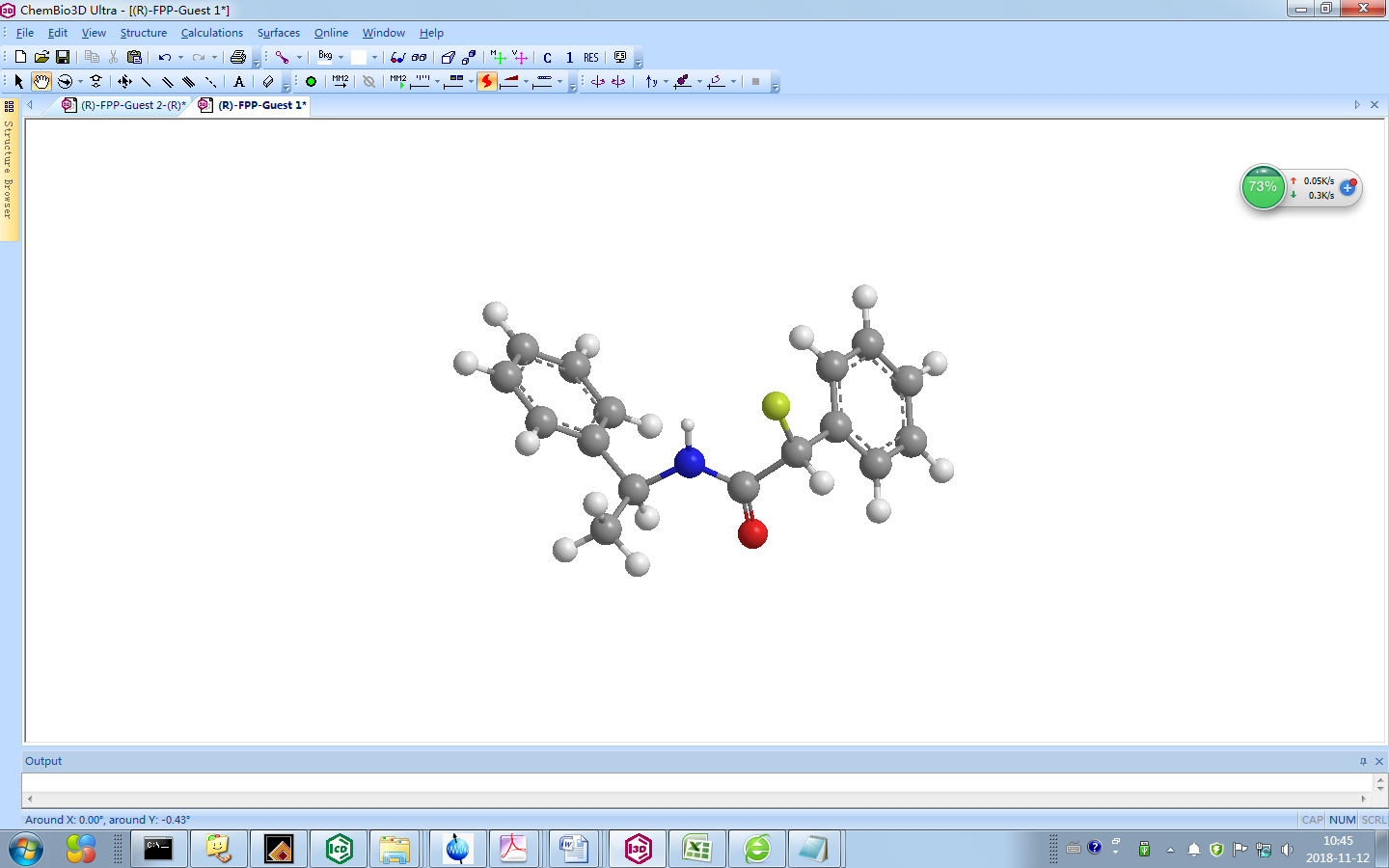
 δ_α-F_*^R^* _(calcd.)_ = -192.32 ppm

C 2.259980 1.015190 4.069010

C 3.585760 1.047510 3.641970

C 3.955460 0.364660 2.483510

C 3.005310 -0.348710 1.757540

C 1.674230 -0.378510 2.182950

C 1.304540 0.307450 3.341530

C 0.643070 -1.132680 1.373530

C 0.214040 -0.348540 0.118430

F -0.482580 -1.414440 2.170450

N -1.059380 0.090710 0.120910

O 1.025860 -0.161600 -0.778450

C -1.645020 0.861240 -0.987910

C -2.351620 -0.066810 -1.980990

C -2.502760 1.984590 -0.414380

C -1.865890 3.054660 0.228890

C -2.601500 4.098520 0.779710

C -3.994910 4.091330 0.697460

C -4.638830 3.034420 0.061410

C -3.897510 1.987900 -0.490920

H 1.965800 1.540740 4.970500

H 4.327540 1.597710 4.209590

H 4.985830 0.382040 2.147030

H 3.292920 -0.876080 0.856040

H 0.276180 0.277140 3.677910

H 1.038160 -2.092300 1.033380

H -1.646480 -0.162710 0.902440

H -0.791400 1.313640 -1.497750

H -2.766810 0.502340 -2.816120

H -1.632050 -0.783950 -2.379820

H -3.160210 -0.627220 -1.505180

H -0.782840 3.066300 0.295040

H -2.089590 4.920000 1.268380

H -4.570830 4.905120 1.123320

H -5.720720 3.020320 -0.009690

H -4.419370 1.178580 -0.986440

**Ball-cylinder model for (*S*)-FPA-amide 1 and cartesian coordinates (Gaussian 09).**


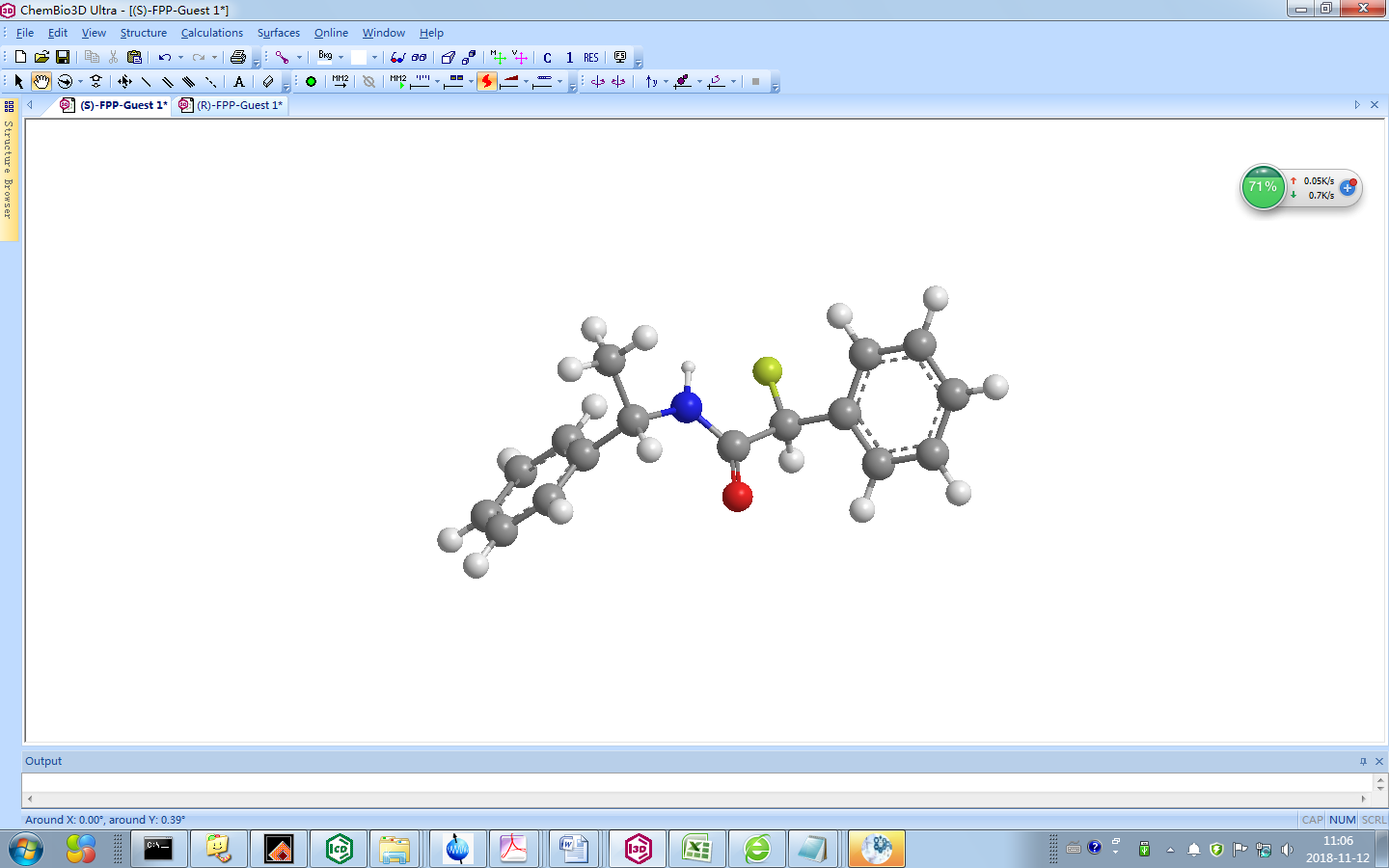
 δ_α-F_*^S^* _(calcd.)_ = -193.24 ppm

C 4.608406 1.015414 1.823902

C 5.431356 0.036844 1.266572

C 4.875236 -1.160446 0.822422

C 3.502736 -1.380976 0.930422

C 2.678586 -0.402606 1.489132

C 3.238236 0.797424 1.938442

C 1.185766 -0.621716 1.602772

C 0.393596 0.223044 0.586752

F 0.883466 -1.985796 1.427842

N -0.253374 -0.478516 -0.365738

O 0.394946 1.441554 0.685322

C -1.059064 0.157424 -1.409768

C -0.970094 -0.679736 -2.692728

C -2.506144 0.385054 -0.982828

C -3.171474 1.545164 -1.388498

C -4.509034 1.753494 -1.056818

C -5.199954 0.801434 -0.309458

C -4.543474 -0.356936 0.103842

C -3.206744 -0.562806 -0.231458

H 5.033926 1.948654 2.174992

H 6.498846 0.206134 1.183012

H 5.508316 -1.927746 0.391262

H 3.073916 -2.315476 0.592482

H 2.598936 1.560474 2.365022

H 0.825916 -0.345326 2.596322

H -0.190414 -1.485276 -0.332558

H -0.606704 1.135234 -1.585728

H -1.542134 -0.205566 -3.491958

H 0.069116 -0.777166 -3.015708

H -1.384064 -1.680036 -2.533368

H -2.636134 2.295444 -1.961868

H -5.007614 2.662454 -1.374638

H -6.238934 0.963444 -0.045698

H -5.071414 -1.100346 0.690822

H -2.704534 -1.462216 0.107472

**Ball-cylinder model for (*R*)-FPA-amide 2 and cartesian coordinates (Gaussian 09).**

**
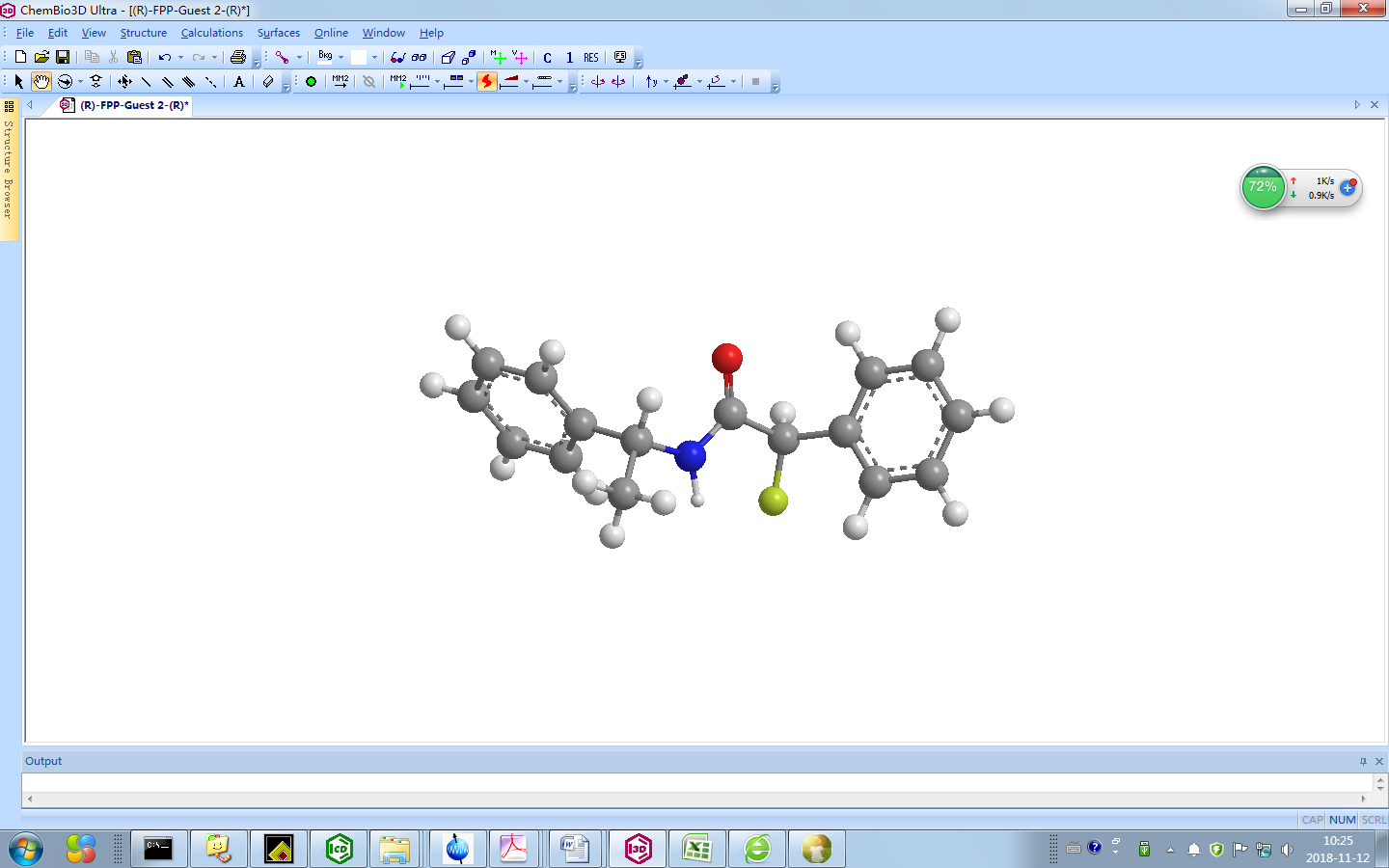
** δ_α-F_*^R^* _(calcd.)_ = -193.24 ppm

C 3.49186 0.71693 -2.58614

C 4.31481 -0.26164 -2.02881

C 3.75869 -1.45893 -1.58466

C 2.38619 -1.67946 -1.69266

C 1.56204 -0.70109 -2.25137

C 2.12169 0.49894 -2.70068

C 0.06922 -0.9202 -2.36501

C -0.72295 -0.07544 -1.34899

F -0.23308 -2.28428 -2.19008

N -1.36992 -0.777 -0.3965

O -0.7216 1.14307 -1.44756

C -2.17561 -0.14106 0.64753

C -2.08664 -0.97822 1.93049

C -3.62269 0.08657 0.22059

C -4.28802 1.24668 0.62626

C -5.62558 1.45501 0.29458

C -6.3165 0.50295 -0.45278

C -5.66002 -0.65542 -0.86608

C -4.32329 -0.86129 -0.53078

H 3.91738 1.65017 -2.93723

H 5.3823 -0.09235 -1.94525

H 4.39177 -2.22623 -1.1535

H 1.95737 -2.61396 -1.35472

H 1.48239 1.26199 -3.12726

H -0.29063 -0.64381 -3.35856

H -1.30696 -1.78376 -0.42968

H -1.72325 0.83675 0.82349

H -2.65868 -0.50405 2.72972

H -1.04743 -1.07565 2.25347

H -2.50061 -1.97852 1.77113

H -3.75268 1.99696 1.19963

H -6.12416 2.36397 0.6124

H -7.35548 0.66496 -0.71654

H -6.18796 -1.39883 -1.45306

H -3.82108 -1.7607 -0.86971

**Ball-cylinder model for (*S*)-FPA-amide 2 and cartesian coordinates (Gaussian 09).**


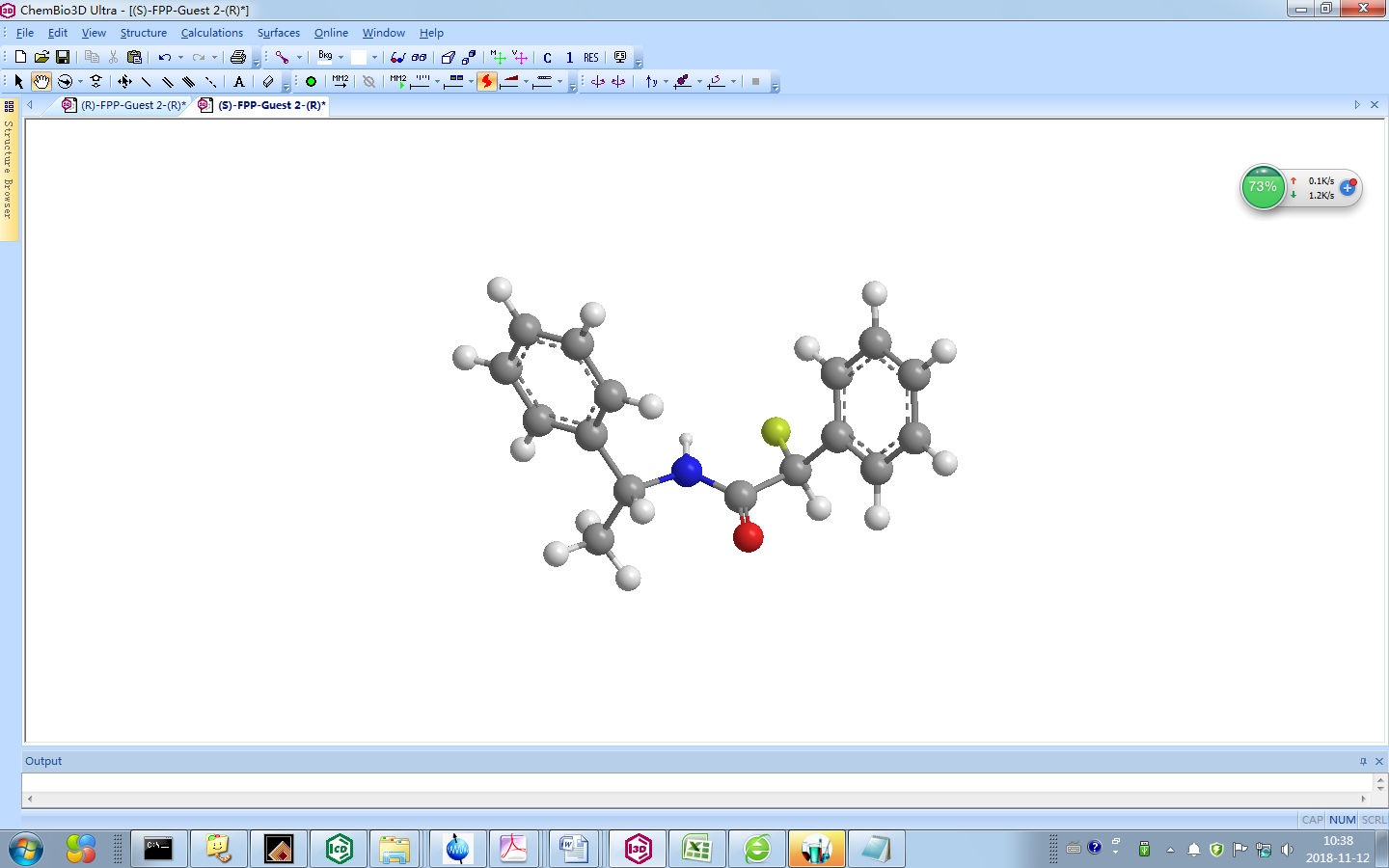
 δ_α-F_*^S^* _(calcd.)_ = -192.32 ppm

C 2.25998 1.01519 -4.06901

C 3.58576 1.04751 -3.64197

C 3.95546 0.36466 -2.48351

C 3.00531 -0.34871 -1.75754

C 1.67423 -0.37851 -2.18295

C 1.30454 0.30745 -3.34153

C 0.64307 -1.13268 -1.37353

C 0.21404 -0.34854 -0.11843

F -0.48258 -1.41444 -2.17045

N -1.05938 0.09071 -0.12091

O 1.02586 -0.1616 0.77845

C -1.64502 0.86124 0.98791

C -2.35162 -0.06681 1.98099

C -2.50276 1.98459 0.41438

C -1.86589 3.05466 -0.22889

C -2.6015 4.09852 -0.77971

C -3.99491 4.09133 -0.69746

C -4.63883 3.03442 -0.06141

C -3.89751 1.9879 0.49092

H 1.9658 1.54074 -4.9705

H 4.32754 1.59771 -4.20959

H 4.98583 0.38204 -2.14703

H 3.29292 -0.87608 -0.85604

H 0.27618 0.27714 -3.67791

H 1.03816 -2.0923 -1.03338

H -1.64648 -0.16271 -0.90244

H -0.7914 1.31364 1.49775

H -2.76681 0.50234 2.81612

H -1.63205 -0.78395 2.37982

H -3.16021 -0.62722 1.50518

H -0.78284 3.0663 -0.29504

H -2.08959 4.92 -1.26838

H -4.57083 4.90512 -1.12332

H -5.72072 3.02032 0.00969

H -4.41937 1.17858 0.98644

**Ball-cylinder model for (*R*)-FPA-amide 3 and cartesian coordinates (Gaussian 09).**


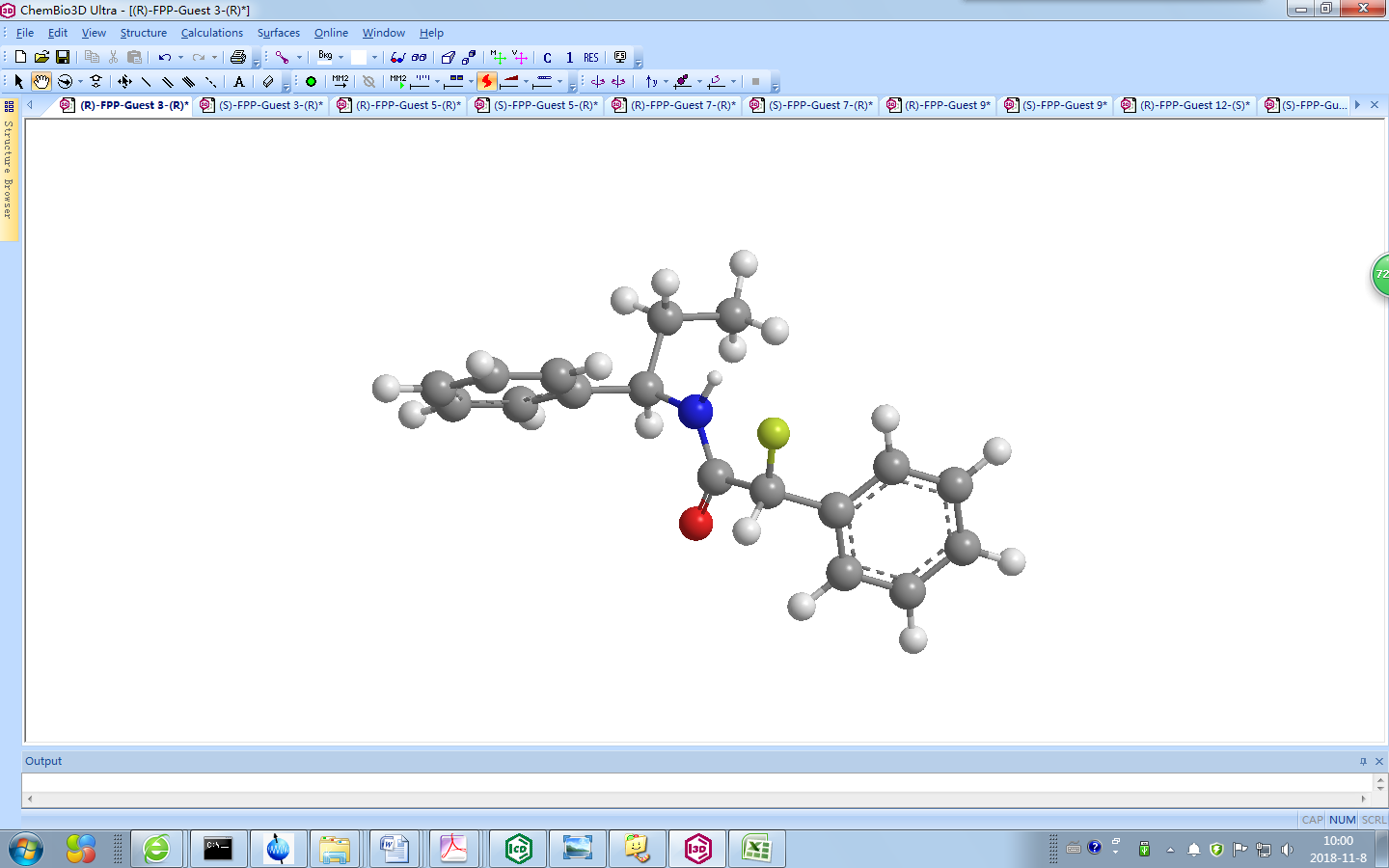
 δ_α-F_*^R^* _(calcd.)_ = -193.84 ppm

C 3.62342 -1.87796 -2.0011

C 3.07441 -2.94481 -2.71249

C 1.7966 -2.82712 -3.25353

C 1.06625 -1.6512 -3.08396

C 1.61488 -0.58333 -2.37176

C 2.9002 -0.70059 -1.83303

C 0.83335 0.69808 -2.17287

C 0.40166 0.89897 -0.70764

F -0.29942 0.7072 -3.00921

N -0.9253 0.81154 -0.48298

O 1.25023 1.11275 0.1461

C -1.5263 0.98997 0.83893

C -2.72465 0.03274 1.00123

C -1.92591 2.43633 1.11602

C -1.86179 2.92526 2.4247

C -2.26837 4.22393 2.72283

C -2.7449 5.05656 1.71135

C -2.80842 4.58055 0.40322

C -2.40174 3.27997 0.10863

C -2.34769 -1.44993 0.94852

H 4.61873 -1.96023 -1.57936

H 3.64101 -3.85924 -2.84641

H 1.3637 -3.64984 -3.81137

H 0.07724 -1.55862 -3.51329

H 3.32355 0.12439 -1.27379

H 1.43325 1.56783 -2.44979

H -1.52296 0.65438 -1.2811

H -0.75216 0.71335 1.55842

H -3.20146 0.26044 1.95849

H -3.46838 0.26649 0.22945

H -1.48243 2.28636 3.21612

H -2.20466 4.58779 3.74233

H -3.05737 6.06923 1.93968

H -3.17137 5.22276 -0.3916

H -2.44298 2.93038 -0.91686

H -3.23269 -2.07657 1.08408

H -1.89169 -1.71841 -0.00733

H -1.63508 -1.70101 1.73959

**Ball-cylinder model for (*S*)-FPA-amide 3 and cartesian coordinates (Gaussian 09).**


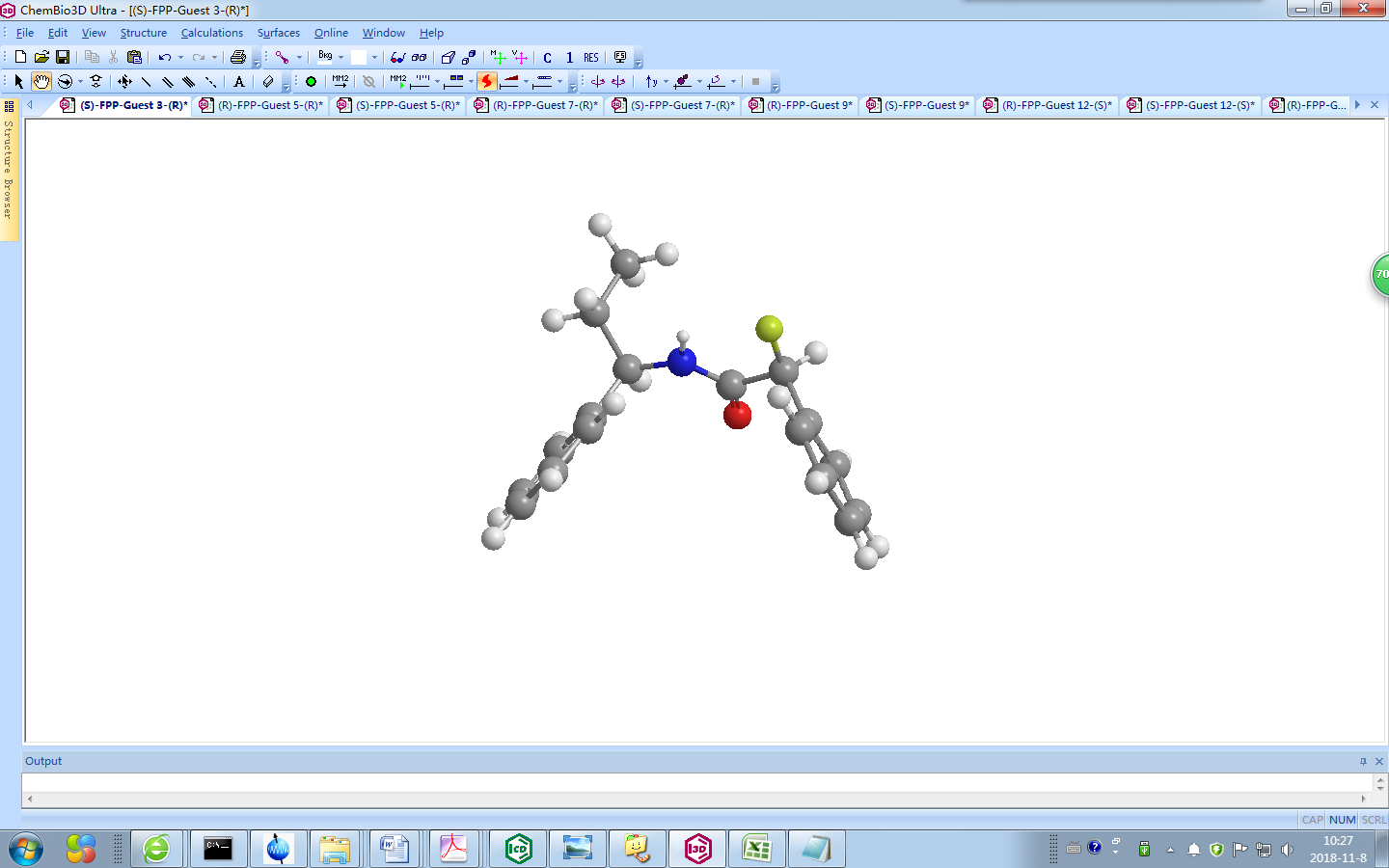
 δ_α-F_*^S^* _(calcd.)_ = -193.54 ppm

C 1.44212 -0.15758 -4.66439

C 2.63229 0.56511 -4.63912

C 3.34786 0.67497 -3.44687

C 2.87776 0.06437 -2.28771

C 1.68081 -0.65859 -2.31139

C 0.96475 -0.76693 -3.50464

C 1.17248 -1.31076 -1.04339

C 0.66595 -0.28063 -0.01579

F 0.14549 -2.22448 -1.34985

N -0.6598 -0.31712 0.22975

O 1.46259 0.48193 0.5121

C -1.31931 0.56192 1.19669

C -2.3757 -0.23247 1.99172

C -1.92259 1.80396 0.54944

C -1.90609 3.01709 1.24434

C -2.49567 4.1581 0.704

C -3.11048 4.10181 -0.5456

C -3.1287 2.89834 -1.24838

C -2.53951 1.75863 -0.70395

C -1.79138 -1.36936 2.83371

H 0.88102 -0.25025 -5.58744

H 3.00249 1.03771 -5.54177

H 4.27635 1.23388 -3.41929

H 3.42974 0.15763 -1.36062

H 0.0438 -1.33472 -3.52852

H 1.96498 -1.8825 -0.55526

H -1.20167 -1.02083 -0.25009

H -0.53385 0.88967 1.88146

H -2.90455 0.4746 2.63672

H -3.12423 -0.62208 1.29103

H -1.42051 3.07154 2.21365

H -2.46643 5.09179 1.25462

H -3.56513 4.98926 -0.97103

H -3.59877 2.84625 -2.22421

H -2.55145 0.83358 -1.26942

H -2.5819 -1.88996 3.3798

H -1.2735 -2.10559 2.21409

H -1.07481 -0.98612 3.56606

**Ball-cylinder model for (*R*)-FPA-amide 4 and cartesian coordinates (Gaussian 09).**

**
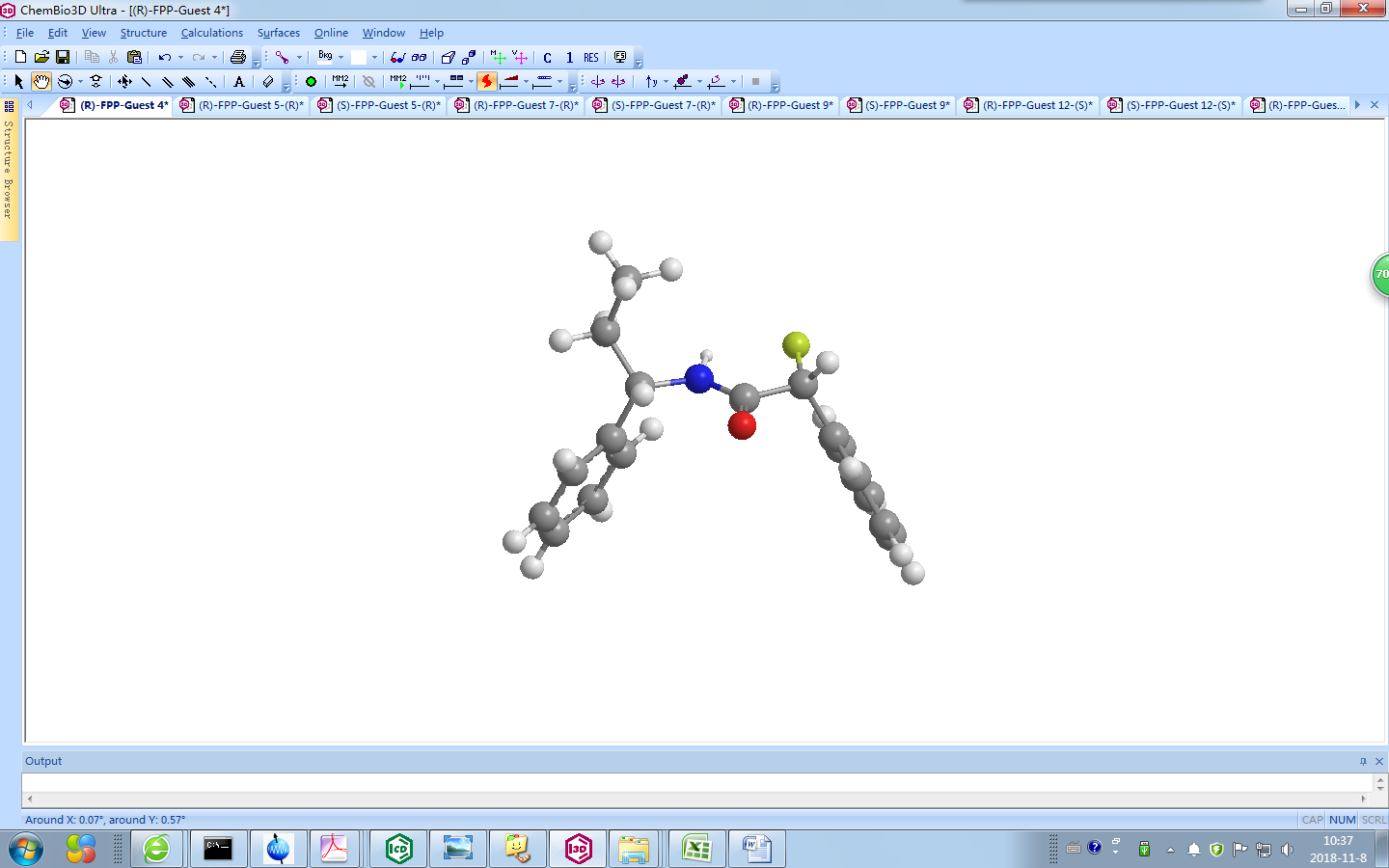
** δ_α-F_*^R^* _(calcd.)_ = -193.54 ppm

C 1.904108 -0.770795 4.065191

C 3.094278 -0.048105 4.039921

C 3.809848 0.061755 2.847671

C 3.339748 -0.548845 1.688511

C 2.142798 -1.271805 1.712191

C 1.426738 -1.380145 2.905441

C 1.634468 -1.923975 0.444191

C 1.127938 -0.893845 -0.583409

F 0.607478 -2.837695 0.750651

N -0.197812 -0.930335 -0.828949

O 1.924578 -0.131285 -1.111299

C -0.857322 -0.051295 -1.795889

C -1.913712 -0.845685 -2.590919

C -1.460602 1.190745 -1.148639

C -1.444102 2.403875 -1.843539

C -2.033682 3.544885 -1.303199

C -2.648492 3.488595 -0.053599

C -2.666712 2.285125 0.649181

C -2.077522 1.145415 0.104751

C -1.329392 -1.982575 -3.432909

H 1.343008 -0.863465 4.988241

H 3.464478 0.424495 4.942571

H 4.738338 0.620665 2.820091

H 3.891728 -0.455585 0.761421

H 0.505788 -1.947935 2.929321

H 2.426968 -2.495715 -0.043939

H -0.739682 -1.634045 -0.349109

H -0.071862 0.276455 -2.480659

H -2.442562 -0.138615 -3.235919

H -2.662242 -1.235295 -1.890229

H -0.958522 2.458325 -2.812849

H -2.004442 4.478575 -1.853819

H -3.103142 4.376045 0.371831

H -3.136782 2.233035 1.625011

H -2.089462 0.220365 0.670221

H -2.119912 -2.503175 -3.978999

H -0.811512 -2.718805 -2.813289

H -0.612822 -1.599335 -4.165259

**Ball-cylinder model for (*S*)-FPA-amide 4 and cartesian coordinates (Gaussian 09).**


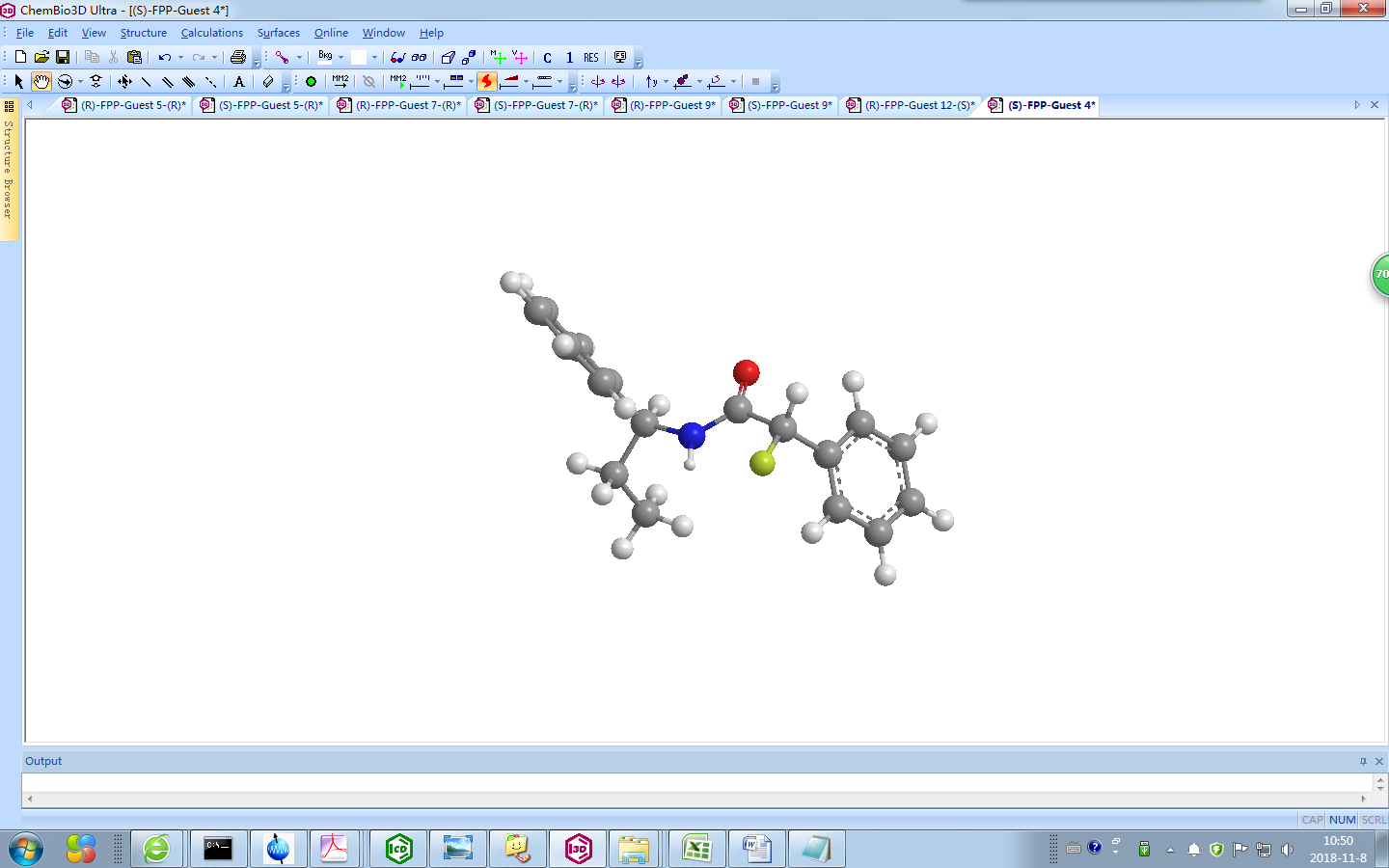
 δ_α-F_*^S^* _(calcd.)_ = -193.84 ppm

C 4.120242 -2.506339 1.664001

C 3.571232 -3.573189 2.375391

C 2.293422 -3.455499 2.916431

C 1.563072 -2.279579 2.746861

C 2.111702 -1.211709 2.034661

C 3.397022 -1.328969 1.495931

C 1.330172 0.069701 1.835771

C 0.898482 0.270591 0.370541

F 0.197402 0.078821 2.672111

N -0.428478 0.183161 0.145881

O 1.747052 0.484371 -0.483199

C -1.029478 0.361591 -1.176029

C -2.227828 -0.595639 -1.338329

C -1.429088 1.807951 -1.453119

C -1.364968 2.296881 -2.761799

C -1.771548 3.595551 -3.059929

C -2.248078 4.428181 -2.048449

C -2.311598 3.952171 -0.740319

C -1.904918 2.651591 -0.445729

C -1.850868 -2.078309 -1.285619

H 5.115552 -2.588609 1.242261

H 4.137832 -4.487619 2.509311

H 1.860522 -4.278219 3.474271

H 0.574062 -2.186999 3.176191

H 3.820372 -0.503989 0.936691

H 1.930072 0.939451 2.112691

H -1.026138 0.026001 0.944001

H -0.255338 0.084971 -1.895519

H -2.704638 -0.367939 -2.295589

H -2.971558 -0.361889 -0.566549

H -0.985608 1.657981 -3.553219

H -1.707838 3.959411 -4.079429

H -2.560548 5.440851 -2.276779

H -2.674548 4.594381 0.054501

H -1.946158 2.302001 0.579761

H -2.735868 -2.704949 -1.421179

H -1.394868 -2.346789 -0.329769

H -1.138258 -2.329389 -2.076689

**Ball-cylinder model for (*R*)-FPA-amide 5 and cartesian coordinates (Gaussian 09).**

**
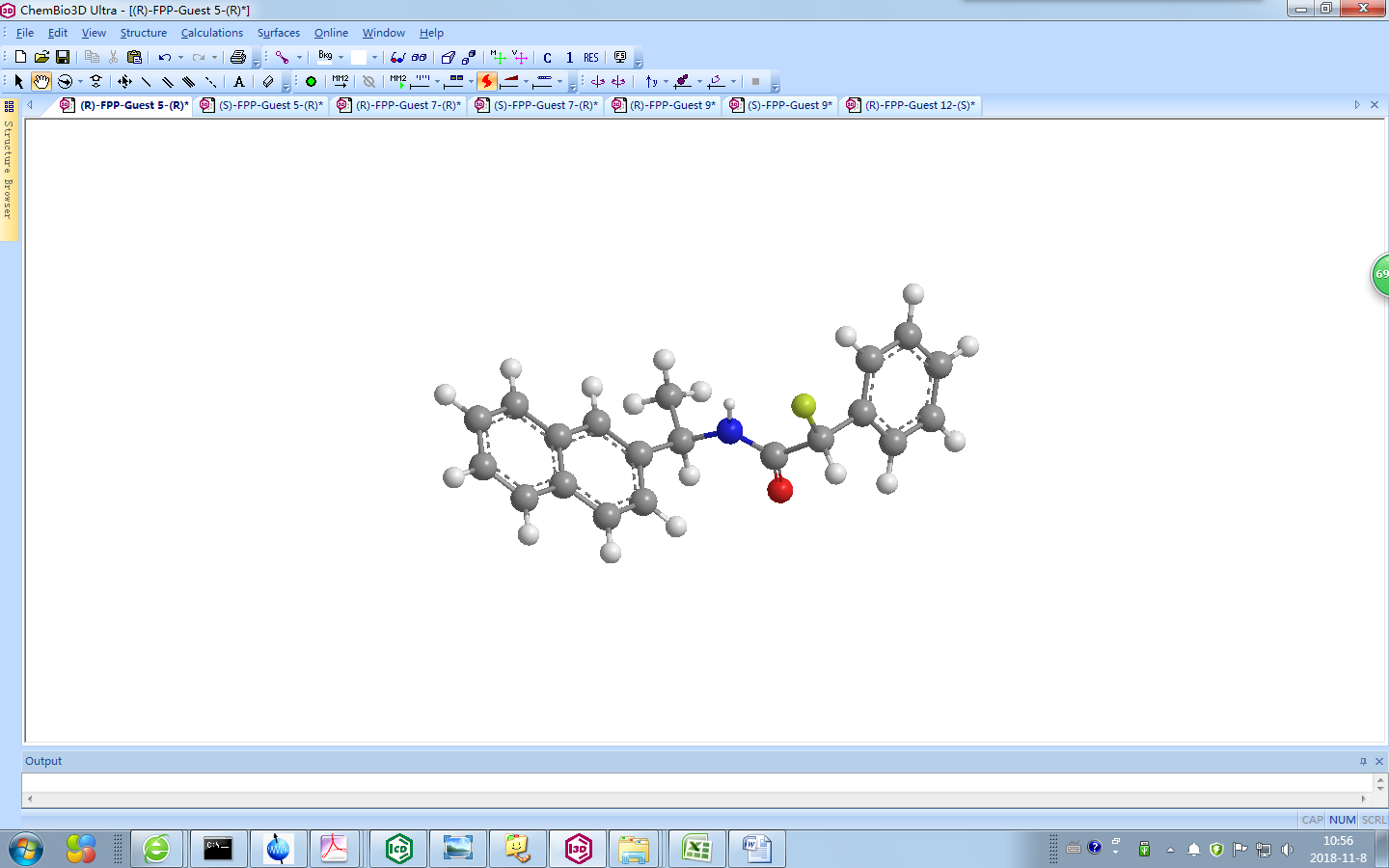
** δ_α-F_*^R^* _(calcd.)_ = -193.96 ppm

C -0.17122 -4.94277 3.2431

C -1.39177 -5.61132 3.18583

C -2.54985 -4.90717 2.85644

C -2.4882 -3.5426 2.58785

C -1.26262 -2.87145 2.64092

C -0.10399 -3.57718 2.96974

C -1.20293 -1.39112 2.33112

C -1.38713 -1.10616 0.82839

F 0.01965 -0.85504 2.77501

N -0.30475 -0.60542 0.20082

O -2.4683 -1.33948 0.30386

C -0.27584 -0.2822 -1.23374

C 0.57143 -1.30488 -1.99525

C 0.13245 1.17561 -1.41472

C 1.32712 1.56033 -1.97816

C 1.67611 2.9319 -2.11786

C 0.76263 3.93187 -1.66176

C -0.46676 3.51244 -1.08522

C -0.76928 2.18126 -0.96491

C 2.90558 3.34086 -2.69727

C 3.21664 4.67473 -2.81959

C 2.31156 5.66362 -2.36804

C 1.11202 5.29956 -1.80279

H 0.73304 -5.48204 3.50183

H -1.44239 -6.67288 3.39961

H -3.50422 -5.41954 2.81317

H -3.38694 -2.99777 2.32634

H 0.84401 -3.05783 3.02325

H -1.99334 -0.85061 2.85677

H 0.52021 -0.4252 0.75523

H -1.31323 -0.38459 -1.56003

H 0.57189 -1.0874 -3.06572

H 0.15578 -2.30316 -1.84767

H 1.60634 -1.31498 -1.64333

H 2.03295 0.81996 -2.33633

H -1.16912 4.26544 -0.74326

H -1.71382 1.87949 -0.52472

H 3.59929 2.58191 -3.0439

H 4.15894 4.97443 -3.26414

H 2.56875 6.71179 -2.47065

H 0.41575 6.05615 -1.45632

**Ball-cylinder model for (*S*)-FPA-amide 5 and cartesian coordinates (Gaussian 09).**


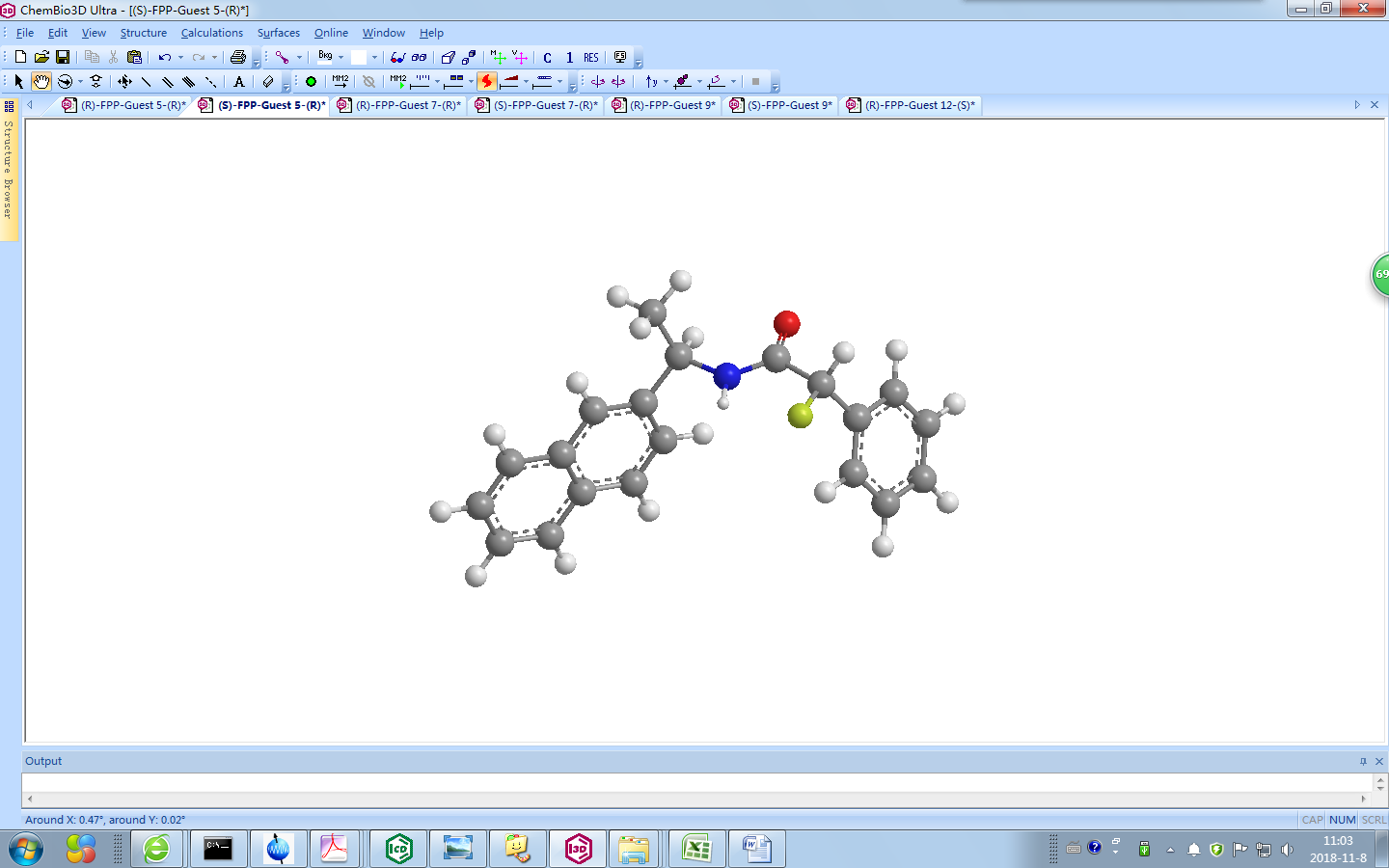
 δ_α-F_*^S^* _(calcd.)_ = -192.60 ppm

C -3.85098 -3.75286 2.85166

C -3.69023 -4.94564 2.15015

C -2.53137 -5.15725 1.40353

C -1.5378 -4.18291 1.36029

C -1.70001 -2.98383 2.06068

C -2.86128 -2.7721 2.80627

C -0.62684 -1.91976 1.98831

C -0.65287 -1.16811 0.64365

F -0.78024 -1.00794 3.04929

N -1.02706 0.12482 0.71365

O -0.35563 -1.76458 -0.38279

C -1.10249 1.00512 -0.46197

C 0.18636 1.81768 -0.61552

C -2.38985 1.8174 -0.39114

C -2.41083 3.18832 -0.27815

C -3.63418 3.9112 -0.21808

C -4.86914 3.19456 -0.27565

C -4.82445 1.77888 -0.39244

C -3.62694 1.11477 -0.44696

C -3.66802 5.32552 -0.10352

C -4.86593 5.99861 -0.04938

C -6.08775 5.28823 -0.10711

C -6.08835 3.91754 -0.21769

H -4.74726 -3.58249 3.43737

H -4.46039 -5.70764 2.18729

H -2.39747 -6.08453 0.85815

H -0.64025 -4.34719 0.77655

H -2.98379 -1.84899 3.3579

H 0.36767 -2.35937 2.09065

H -1.20109 0.51857 1.62728

H -1.1784 0.3264 -1.3154

H 0.14967 2.441 -1.51194

H 1.03353 1.13607 -0.7092

H 0.36263 2.46165 0.24994

H -1.48717 3.75362 -0.23729

H -5.75757 1.22752 -0.44241

H -3.61268 0.03391 -0.53663

H -2.73044 5.86991 -0.05987

H -4.87857 7.0792 0.03733

H -7.02527 5.83092 -0.06403

H -7.02416 3.37026 -0.26263

**Ball-cylinder model for (*R*)-FPA-amide 6 and cartesian coordinates (Gaussian 09).**

**
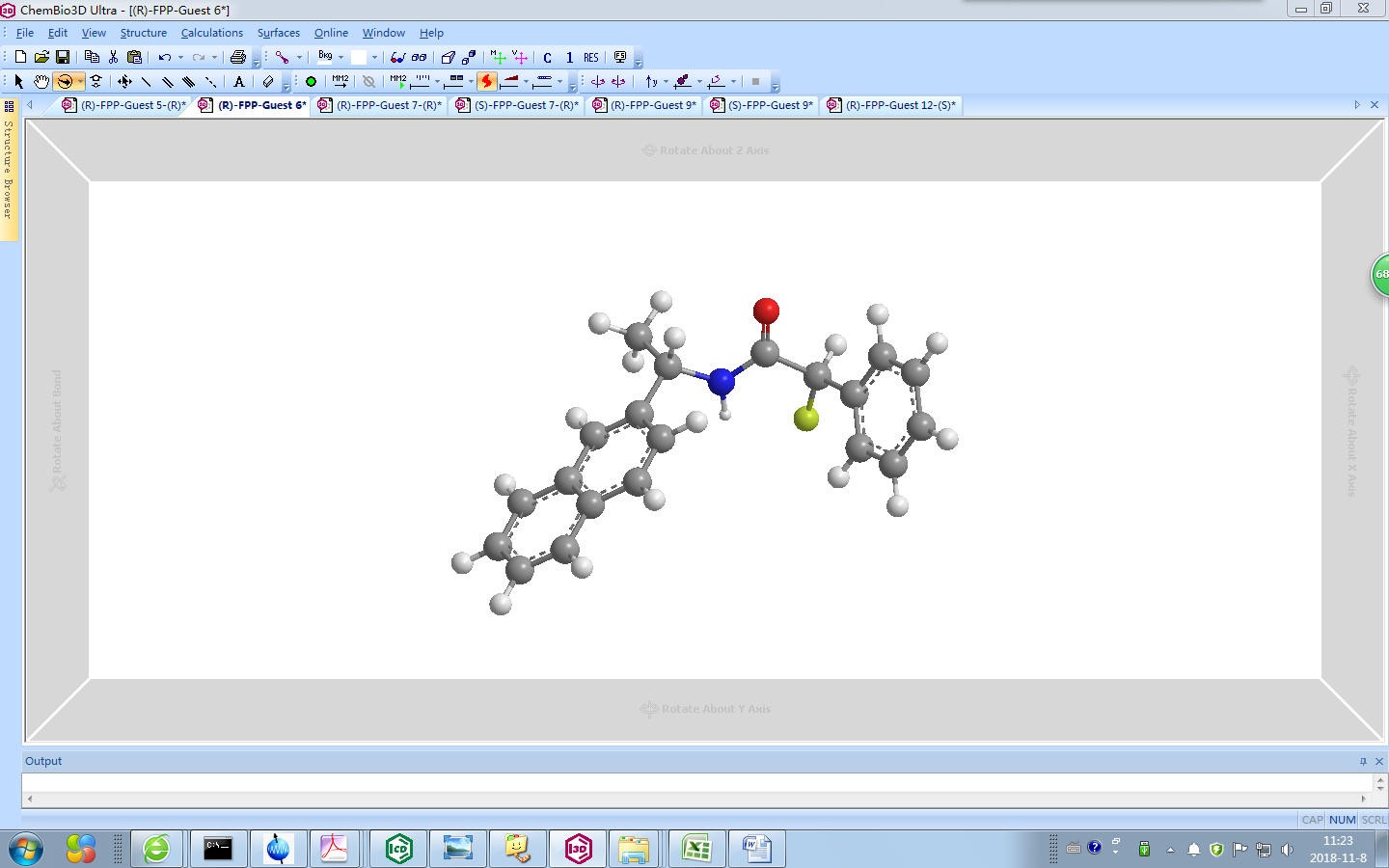
** δ_α-F_*^R^* _(calcd.)_ = -192.60 ppm

C -1.138617 -4.214970 -2.252388

C -0.977867 -5.407750 -1.550878

C 0.180993 -5.619360 -0.804258

C 1.174563 -4.645020 -0.761018

C 1.012353 -3.445940 -1.461408

C -0.148917 -3.234210 -2.206998

C 2.085523 -2.381870 -1.389038

C 2.059493 -1.630220 -0.044378

F 1.932123 -1.470050 -2.450018

N 1.685303 -0.337290 -0.114378

O 2.356733 -2.226690 0.982062

C 1.609873 0.543010 1.061242

C 2.898723 1.355570 1.214792

C 0.322513 1.355290 0.990412

C 0.301533 2.726210 0.877422

C -0.921817 3.449090 0.817352

C -2.156777 2.732450 0.874922

C -2.112087 1.316770 0.991712

C -0.914577 0.652660 1.046232

C -0.955657 4.863410 0.702792

C -2.153567 5.536500 0.648652

C -3.375387 4.826120 0.706382

C -3.375987 3.455430 0.816962

H -2.034897 -4.044600 -2.838098

H -1.748027 -6.169750 -1.588018

H 0.314893 -6.546640 -0.258878

H 2.072113 -4.809300 -0.177278

H -0.271427 -2.311100 -2.758628

H 3.080033 -2.821480 -1.491378

H 1.511273 0.056460 -1.028008

H 1.533963 -0.135710 1.914672

H 2.862033 1.978890 2.111212

H 3.745893 0.673960 1.308472

H 3.074993 1.999540 0.349332

H 1.225193 3.291510 0.836562

H -3.045207 0.765410 1.041682

H -0.900317 -0.428200 1.135902

H -0.018077 5.407800 0.659142

H -2.166207 6.617090 0.561942

H -4.312907 5.368810 0.663302

H -4.311797 2.908150 0.861902

**Ball-cylinder model for (*S*)-FPA-amide 6 and cartesian coordinates (Gaussian 09).**


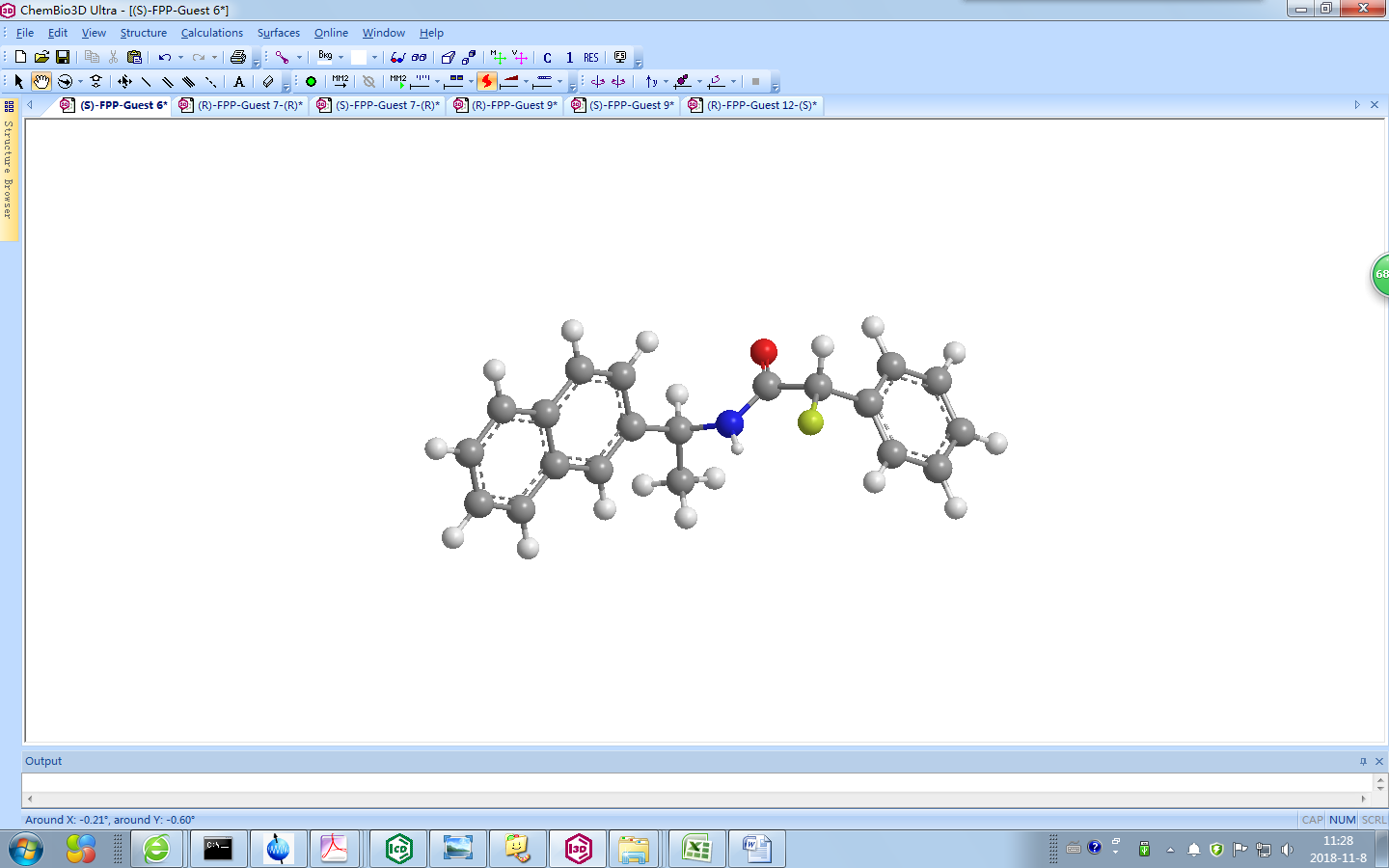
 δ_α-F_*^S^* _(calcd.)_ = -193.96 ppm

C -0.216987 -4.923954 -3.279590

C -1.437537 -5.592504 -3.222320

C -2.595617 -4.888354 -2.892930

C -2.533967 -3.523784 -2.624340

C -1.308387 -2.852634 -2.677410

C -0.149757 -3.558364 -3.006230

C -1.248697 -1.372304 -2.367610

C -1.432897 -1.087344 -0.864880

F -0.026117 -0.836224 -2.811500

N -0.350517 -0.586604 -0.237310

O -2.514067 -1.320664 -0.340350

C -0.321607 -0.263384 1.197250

C 0.525663 -1.286064 1.958760

C 0.086683 1.194426 1.378230

C 1.281353 1.579146 1.941670

C 1.630343 2.950716 2.081370

C 0.716863 3.950686 1.625270

C -0.512527 3.531256 1.048730

C -0.815047 2.200076 0.928420

C 2.859813 3.359676 2.660780

C 3.170873 4.693546 2.783100

C 2.265793 5.682436 2.331550

C 1.066253 5.318376 1.766300

H 0.687273 -5.463224 -3.538320

H -1.488157 -6.654064 -3.436100

H -3.549987 -5.400724 -2.849660

H -3.432707 -2.978954 -2.362830

H 0.798243 -3.039014 -3.059740

H -2.039107 -0.831794 -2.893260

H 0.474443 -0.406384 -0.791720

H -1.358997 -0.365774 1.523540

H 0.526123 -1.068584 3.029230

H 0.110013 -2.284344 1.811180

H 1.560573 -1.296164 1.606840

H 1.987183 0.838776 2.299840

H -1.214887 4.284256 0.706770

H -1.759587 1.898306 0.488230

H 3.553523 2.600726 3.007410

H 4.113173 4.993246 3.227650

H 2.522983 6.730606 2.434160

H 0.369983 6.074966 1.419830

**Ball-cylinder model for (*R*)-FPA-amide 7 and cartesian coordinates (Gaussian 09).**

**
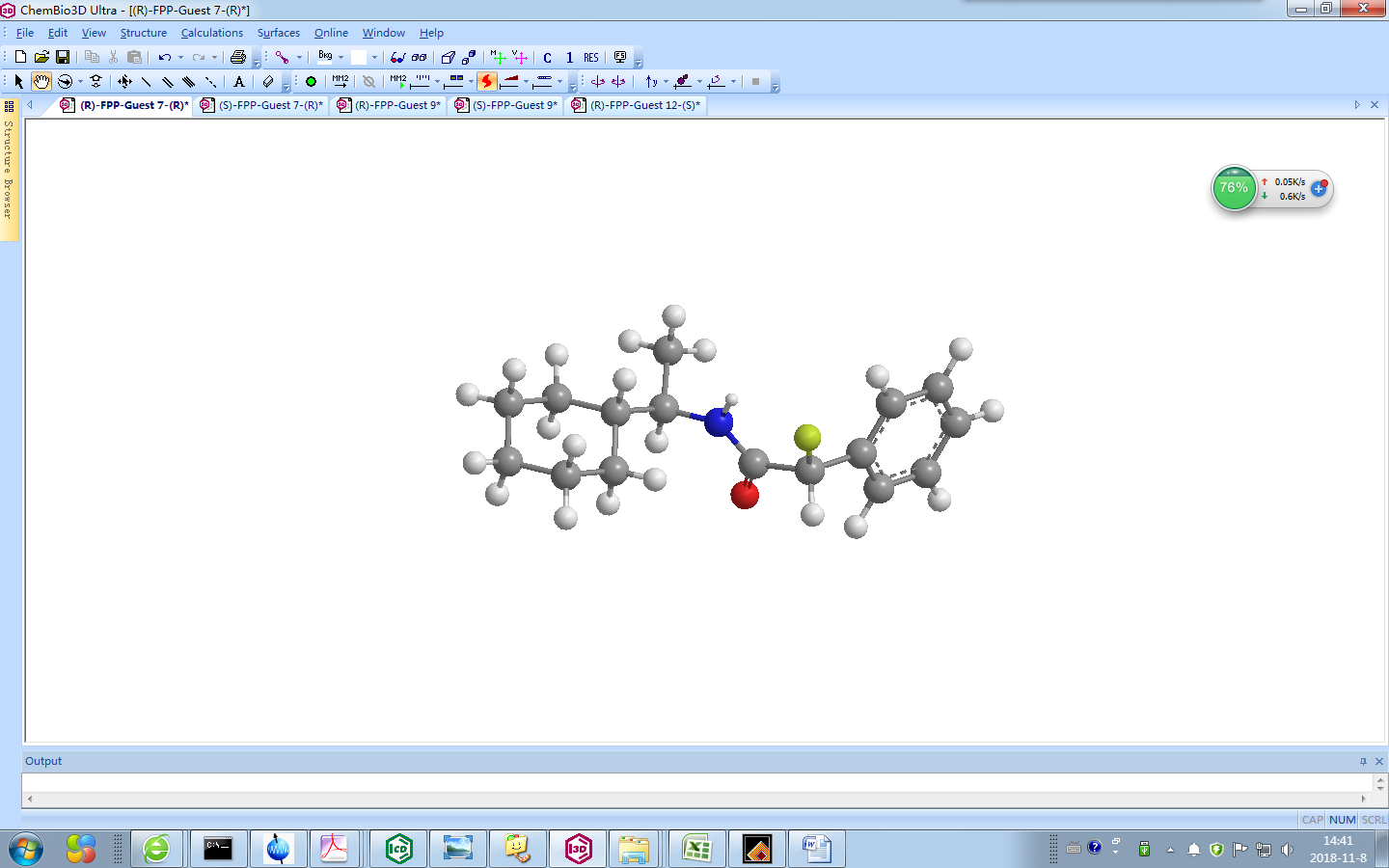
** δ_α-F_*^R^* _(calcd.)_ = -194.04 ppm

C 4.85374 0.49895 -2.9655

C 5.23287 -0.84322 -2.94606

C 4.26182 -1.8317 -2.80563

C 2.91716 -1.48422 -2.68142

C 2.53666 -0.14131 -2.70094

C 3.51199 0.85026 -2.84698

C 1.08266 0.2528 -2.55353

C 0.79032 0.89923 -1.18513

F 0.25769 -0.87314 -2.7398

N 0.00311 0.17733 -0.36634

O 1.27324 1.99582 -0.93178

C -0.38541 0.59943 0.98539

C 0.3324 -0.28283 2.01568

C -1.92706 0.60296 1.12236

C -2.59091 1.48559 0.04689

C -4.11966 1.51997 0.17817

C -4.55644 1.9697 1.5776

C -3.9107 1.0956 2.65898

C -2.38061 1.05185 2.52625

H 5.603 1.27442 -3.07745

H 6.27767 -1.11542 -3.04338

H 4.54774 -2.87745 -2.7933

H 2.16357 -2.25432 -2.58017

H 3.21855 1.89265 -2.85343

H 0.79675 0.98051 -3.31621

H -0.32683 -0.71435 -0.70803

H -0.02268 1.62561 1.08059

H 0.17686 0.08202 3.03203

H 1.40665 -0.28549 1.81998

H -0.02728 -1.31576 1.96511

H -2.27307 -0.43207 0.97399

H -2.19707 2.50671 0.13822

H -2.31254 1.13306 -0.94952

H -4.54176 2.18252 -0.58435

H -4.52302 0.51863 -0.02153

H -4.25873 3.01503 1.73072

H -5.6473 1.93857 1.66564

H -4.18283 1.4579 3.65558

H -4.30637 0.07469 2.58029

H -1.97009 2.05088 2.72676

H -1.97369 0.38673 3.29198

**Ball-cylinder model for (*S*)-FPA-amide 7 and cartesian coordinates (Gaussian 09).**


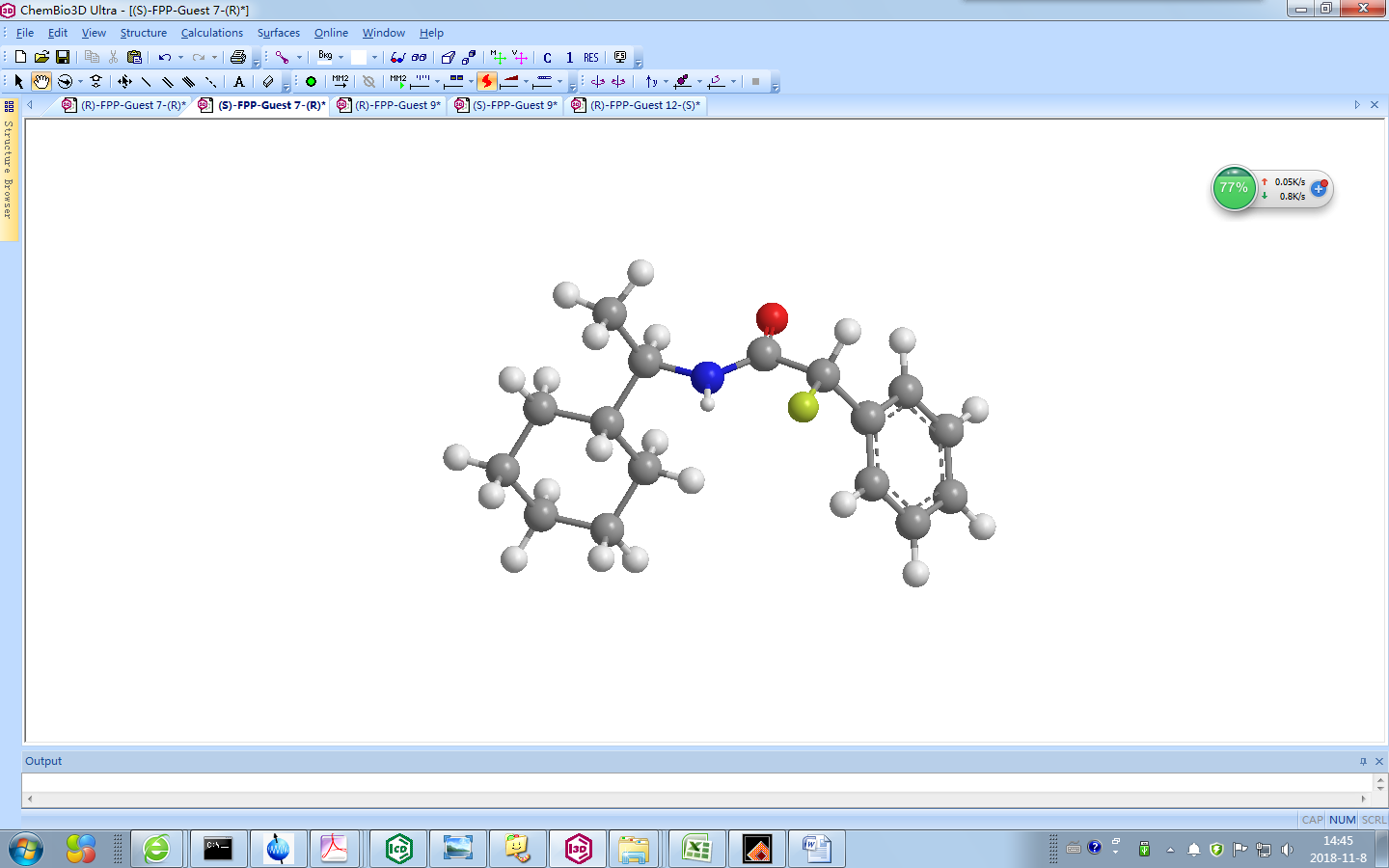
 δ_α-F_*^S^* _(calcd.)_ = -193.46 ppm

C 4.48838 -1.05131 -2.65307

C 3.95518 -2.08186 -3.42357

C 2.67328 -1.95207 -3.95782

C 1.92969 -0.79857 -3.72461

C 2.46243 0.23496 -2.94796

C 3.74548 0.10405 -2.41341

C 1.63972 1.47764 -2.68557

C 0.48344 1.21485 -1.70041

F 2.46677 2.50648 -2.19497

N 0.58948 1.83635 -0.51094

O -0.43167 0.47674 -2.04278

C -0.43105 1.76742 0.54345

C -1.21884 3.08456 0.57205

C 0.22613 1.39227 1.89269

C 0.97369 0.04695 1.80551

C 1.62421 -0.34911 3.13826

C 0.59886 -0.38551 4.27816

C -0.15185 0.94712 4.38053

C -0.79686 1.34978 3.04549

H 5.48543 -1.14249 -2.23708

H 4.53471 -2.97875 -3.61036

H 2.25247 -2.74824 -4.5614

H 0.93139 -0.70201 -4.13308

H 4.16433 0.90772 -1.82192

H 1.19164 1.84966 -3.60978

H 1.36666 2.47024 -0.38817

H -1.10187 0.95946 0.24188

H -2.07662 3.024 1.24344

H -1.59211 3.31654 -0.42765

H -0.58475 3.9141 0.90172

H 0.96233 2.17657 2.12943

H 0.25974 -0.73223 1.50664

H 1.73169 0.08999 1.01955

H 2.11553 -1.322 3.03552

H 2.41266 0.37385 3.38544

H -0.12046 -1.19338 4.09142

H 1.0914 -0.61792 5.22796

H -0.92029 0.89307 5.15845

H 0.54959 1.73275 4.69034

H -1.58556 0.62871 2.79068

H -1.28514 2.3203 3.16323

**Ball-cylinder model for (*R*)-FPA-amide 8 and cartesian coordinates (Gaussian 09).**

**
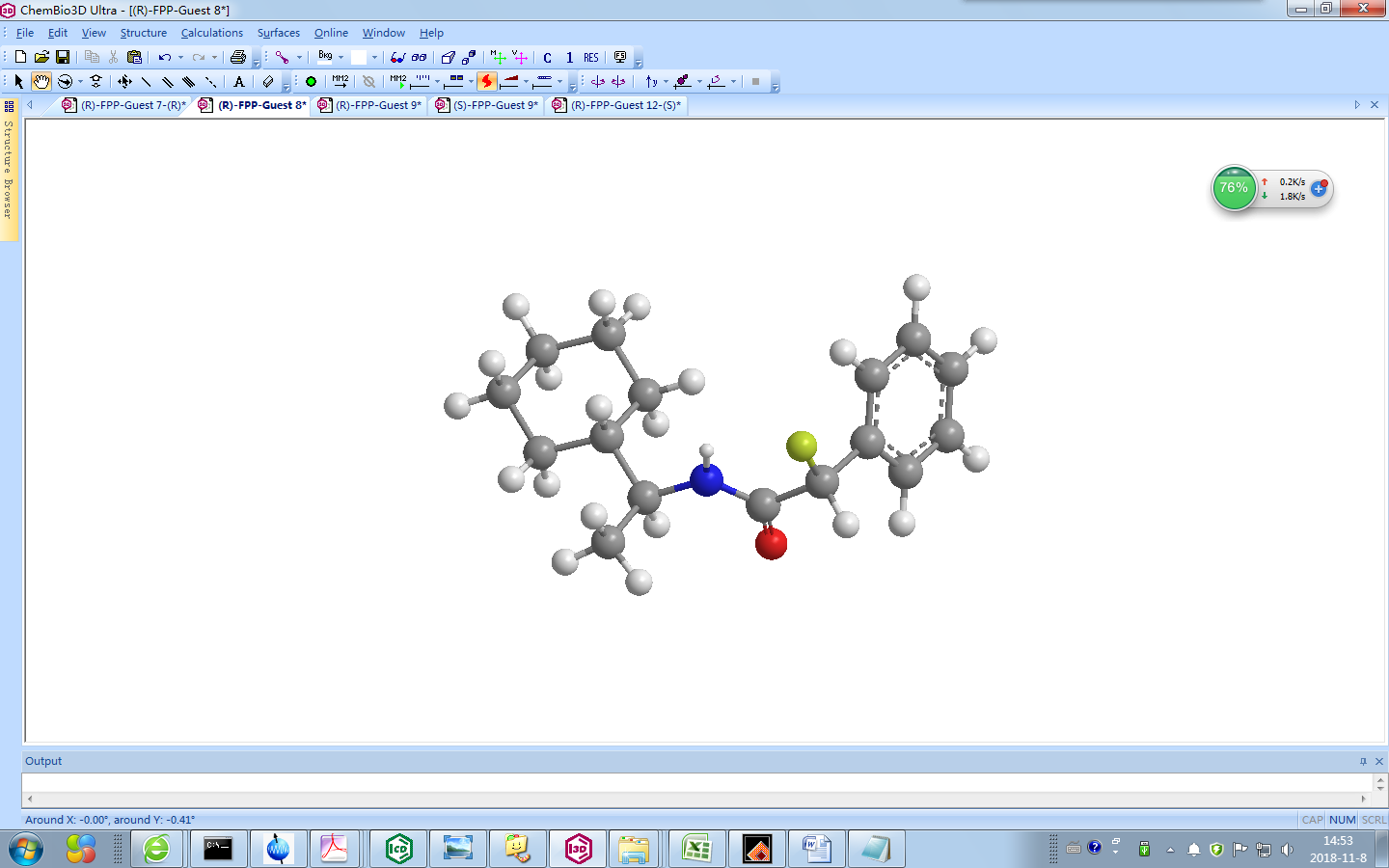
** δ_α-F_*^R^* _(calcd.)_ = -193.46 ppm

C 3.400350 -1.613278 2.877394

C 2.867150 -2.643828 3.647894

C 1.585250 -2.514038 4.182144

C 0.841660 -1.360538 3.948934

C 1.374400 -0.327008 3.172284

C 2.657450 -0.457918 2.637734

C 0.551690 0.915672 2.909894

C -0.604590 0.652882 1.924734

F 1.378740 1.944512 2.419294

N -0.498550 1.274382 0.735264

O -1.519700 -0.085228 2.267104

C -1.519080 1.205452 -0.319126

C -2.306870 2.522592 -0.347726

C -0.861900 0.830302 -1.668366

C -0.114340 -0.515018 -1.581186

C 0.536180 -0.911078 -2.913936

C -0.489170 -0.947478 -4.053836

C -1.239880 0.385152 -4.156206

C -1.884890 0.787812 -2.821166

H 4.397400 -1.704458 2.461404

H 3.446680 -3.540718 3.834684

H 1.164440 -3.310208 4.785724

H -0.156640 -1.263978 4.357404

H 3.076300 0.345752 2.046244

H 0.103610 1.287692 3.834104

H 0.278630 1.908272 0.612494

H -2.189900 0.397492 -0.017556

H -3.164650 2.462032 -1.019116

H -2.680140 2.754572 0.651974

H -1.672780 3.352132 -0.677396

H -0.125700 1.614602 -1.905106

H -0.828290 -1.294198 -1.282316

H 0.643660 -0.471978 -0.795226

H 1.027500 -1.883968 -2.811196

H 1.324630 -0.188118 -3.161116

H -1.208490 -1.755348 -3.867096

H 0.003370 -1.179888 -5.003636

H -2.008320 0.331102 -4.934126

H -0.538440 1.170782 -4.466016

H -2.673590 0.066742 -2.566356

H -2.373170 1.758332 -2.938906

**Ball-cylinder model for (*S*)-FPA-amide 8 and cartesian coordinates (Gaussian 09).**


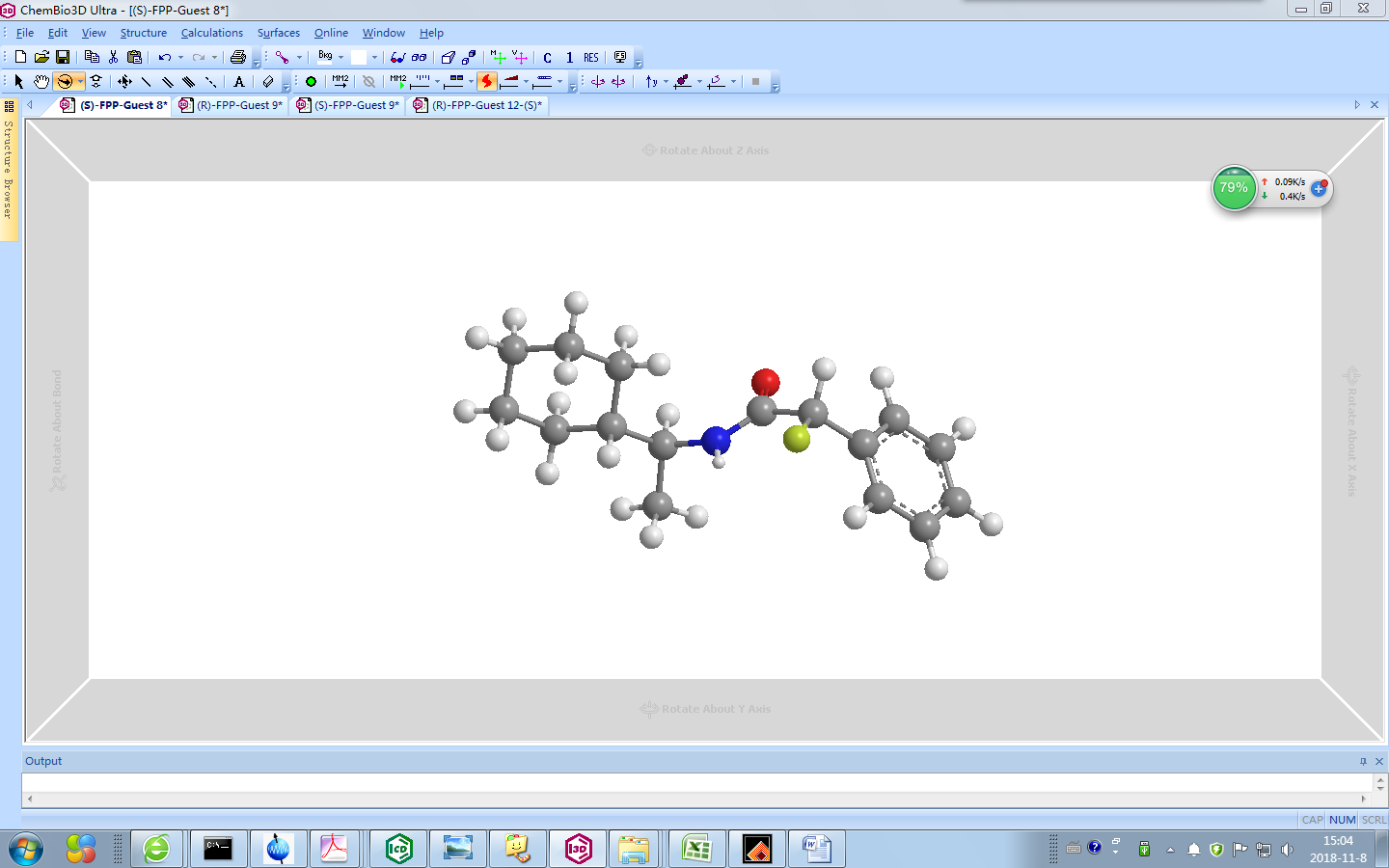
 δ_α-F_*^S^* _(calcd.)_ = -194.04 ppm

C 5.029096 0.019239 2.748957

C 5.408226 -1.322931 2.729517

C 4.437176 -2.311411 2.589087

C 3.092516 -1.963931 2.464877

C 2.712016 -0.621021 2.484397

C 3.687346 0.370549 2.630437

C 1.258016 -0.226911 2.336987

C 0.965676 0.419519 0.968587

F 0.433046 -1.352851 2.523257

N 0.178466 -0.302381 0.149797

O 1.448596 1.516109 0.715237

C -0.210054 0.119719 -1.201933

C 0.507756 -0.762541 -2.232223

C -1.751704 0.123249 -1.338903

C -2.415554 1.005879 -0.263433

C -3.944304 1.040259 -0.394713

C -4.381084 1.489989 -1.794143

C -3.735344 0.615889 -2.875523

C -2.205254 0.572139 -2.742793

H 5.778356 0.794709 2.860907

H 6.453026 -1.595131 2.826837

H 4.723096 -3.357161 2.576757

H 2.338926 -2.734031 2.363627

H 3.393906 1.412939 2.636887

H 0.972106 0.500799 3.099667

H -0.151474 -1.194061 0.491487

H 0.152676 1.145899 -1.297133

H 0.352216 -0.397691 -3.248573

H 1.582006 -0.765201 -2.036523

H 0.148076 -1.795471 -2.181653

H -2.097714 -0.911781 -1.190533

H -2.021714 2.026999 -0.354763

H -2.137184 0.653349 0.732977

H -4.366404 1.702809 0.367807

H -4.347664 0.038919 -0.195013

H -4.083374 2.535319 -1.947263

H -5.471944 1.458859 -1.882183

H -4.007474 0.978189 -3.872123

H -4.131014 -0.405021 -2.796833

H -1.794734 1.571169 -2.943303

H -1.798334 -0.092981 -3.508523

**Ball-cylinder model for (*R*)-FPA-amide 9 and cartesian coordinates (Gaussian 09).**

**
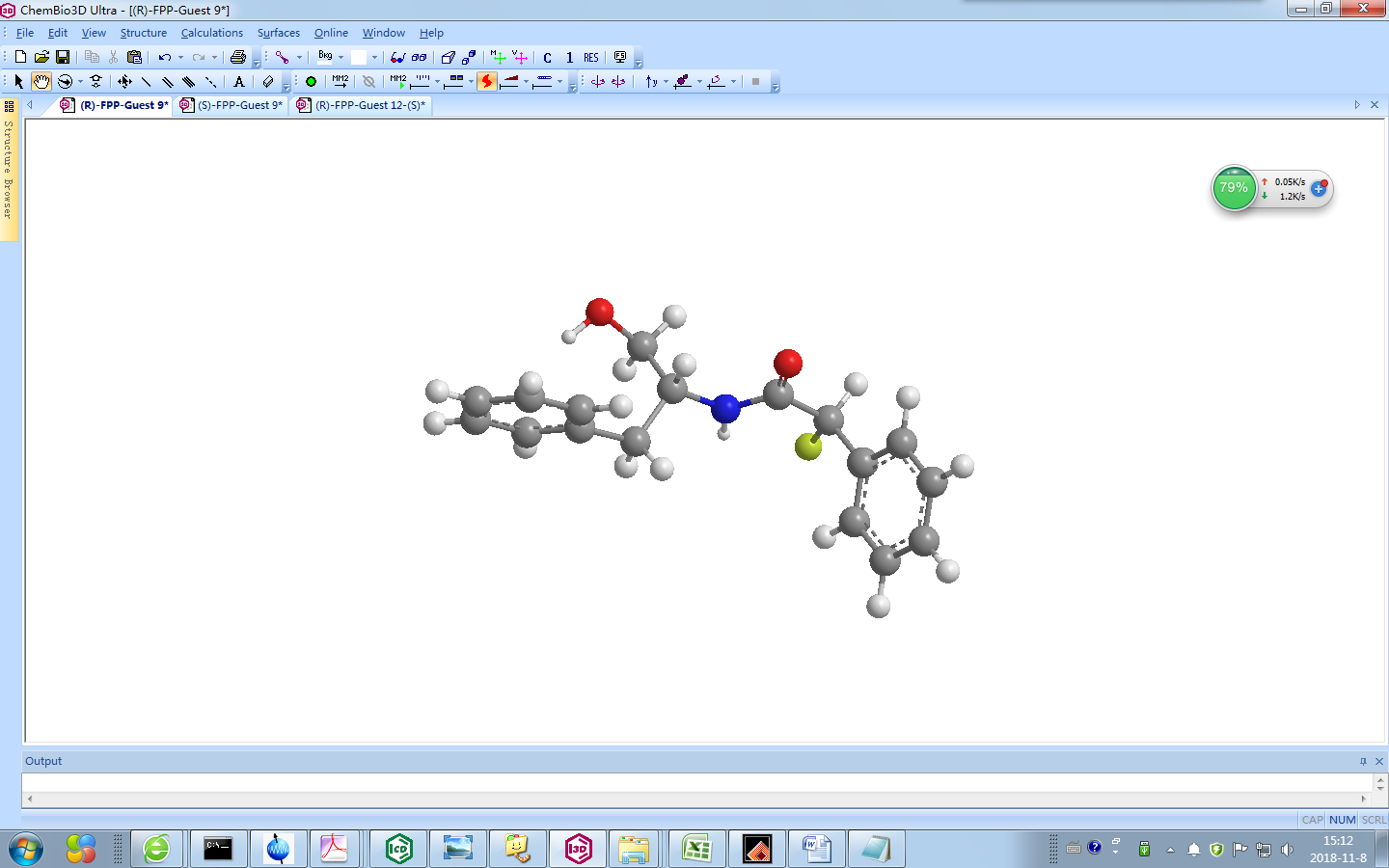
** δ_α-F_*^R^* _(calcd.)_ = -191.64 ppm

C 3.17344 -0.88465 -1.57998

C 2.92668 -1.75619 -2.64027

C 3.79235 -1.77838 -3.73171

C 4.89832 -0.93103 -3.76805

C 5.14741 -0.05935 -2.70606

C 4.28099 -0.0413 -1.60953

C 6.33234 0.87935 -2.74387

C 5.92052 2.31185 -3.13484

F 7.29987 0.39621 -3.64638

N 6.38989 2.73856 -4.32674

O 5.21207 2.95819 -2.37704

C 6.11647 4.06572 -4.87553

C 7.41151 4.90354 -4.86659

C 5.46852 3.90508 -6.27182

C 5.05171 5.19742 -6.94297

C 5.7823 5.72574 -8.01464

C 5.39895 6.92224 -8.62369

C 4.27376 7.60648 -8.17175

C 3.53274 7.08631 -7.10974

C 3.91821 5.89461 -6.50315

O 7.18143 6.29846 -5.00681

H 2.50677 -0.86504 -0.72541

H 2.06735 -2.41647 -2.61319

H 3.60922 -2.45636 -4.55765

H 5.57487 -0.95519 -4.6127

H 4.4702 0.63618 -0.78564

H 6.81464 0.94046 -1.76605

H 6.99181 2.11013 -4.83948

H 5.41144 4.53993 -4.19111

H 7.8946 4.77362 -3.89662

H 8.10277 4.52966 -5.63679

H 6.17036 3.36859 -6.9201

H 4.59264 3.26087 -6.15178

H 6.6538 5.19304 -8.38207

H 5.97772 7.31292 -9.4531

H 3.97255 8.53436 -8.64419

H 2.65188 7.60973 -6.75521

H 3.33298 5.49774 -5.68004

H 6.82382 6.45956 -5.88717

**Ball-cylinder model for (*S*)-FPA-amide 9 and cartesian coordinates (Gaussian 09).**


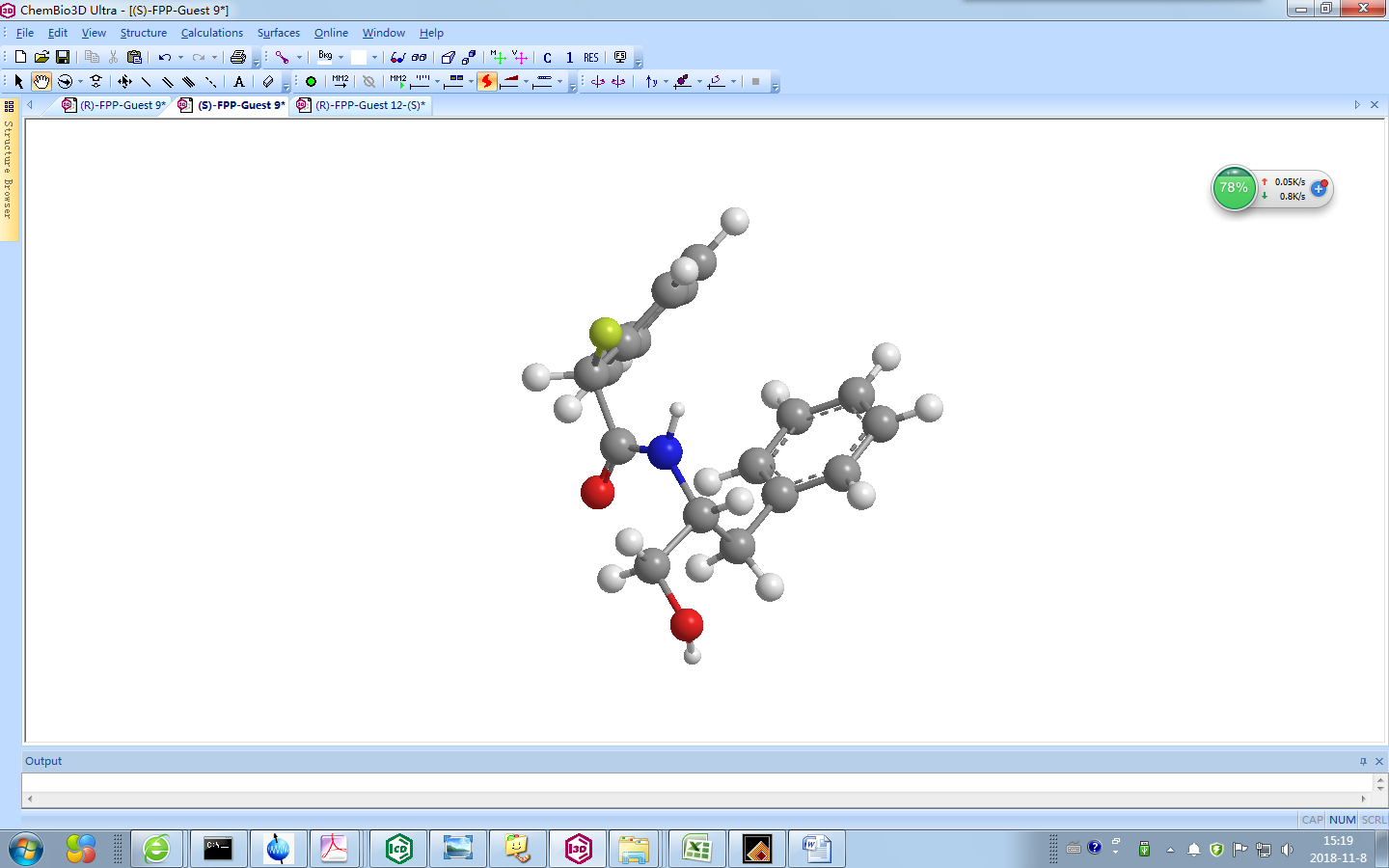
 δ_α-F_*^S^* _(calcd.)_ = -188.41 ppm

C -3.58168 0.40961 0.86484

C -4.08055 0.4231 -0.42898

C -3.50732 -0.39271 -1.3981

C -2.44927 -1.22644 -1.06998

C -1.95403 -1.244 0.23103

C -2.51694 -0.41965 1.19646

C -0.79907 -2.15296 0.57415

C 0.4955 -1.65865 -0.07605

F -0.64145 -2.22322 1.94856

N 1.35267 -1.04645 0.75083

O 0.65938 -1.82014 -1.27418

C 2.59413 -0.40742 0.32577

C 3.61256 -1.47317 -0.08362

C 2.38062 0.63712 -0.78097

C 1.27838 1.60612 -0.45097

C 0.01995 1.47318 -1.03135

C -1.01305 2.33724 -0.69202

C -0.80002 3.34718 0.2363

C 0.45322 3.4907 0.81992

C 1.48314 2.62567 0.47716

O 4.87618 -0.90474 -0.36855

H -4.0197 1.04622 1.62269

H -4.91151 1.06781 -0.68475

H -3.8912 -0.38586 -2.41005

H -2.00176 -1.86563 -1.82071

H -2.13161 -0.43108 2.20677

H -0.97711 -3.16518 0.2085

H 1.08666 -0.98017 1.72021

H 2.97977 0.10617 1.20858

H 3.7556 -2.16353 0.74817

H 3.22441 -2.03615 -0.93601

H 3.3231 1.17529 -0.89893

H 2.15831 0.12563 -1.71781

H -0.15214 0.6788 -1.74882

H -1.98632 2.21208 -1.1487

H -1.60429 4.02102 0.5026

H 0.62957 4.28037 1.53937

H 2.46141 2.74545 0.93037

H 4.87656 -0.57533 -1.27205

**Ball-cylinder model for (*R*)-FPA-amide 10 and cartesian coordinates (Gaussian 09).**

**
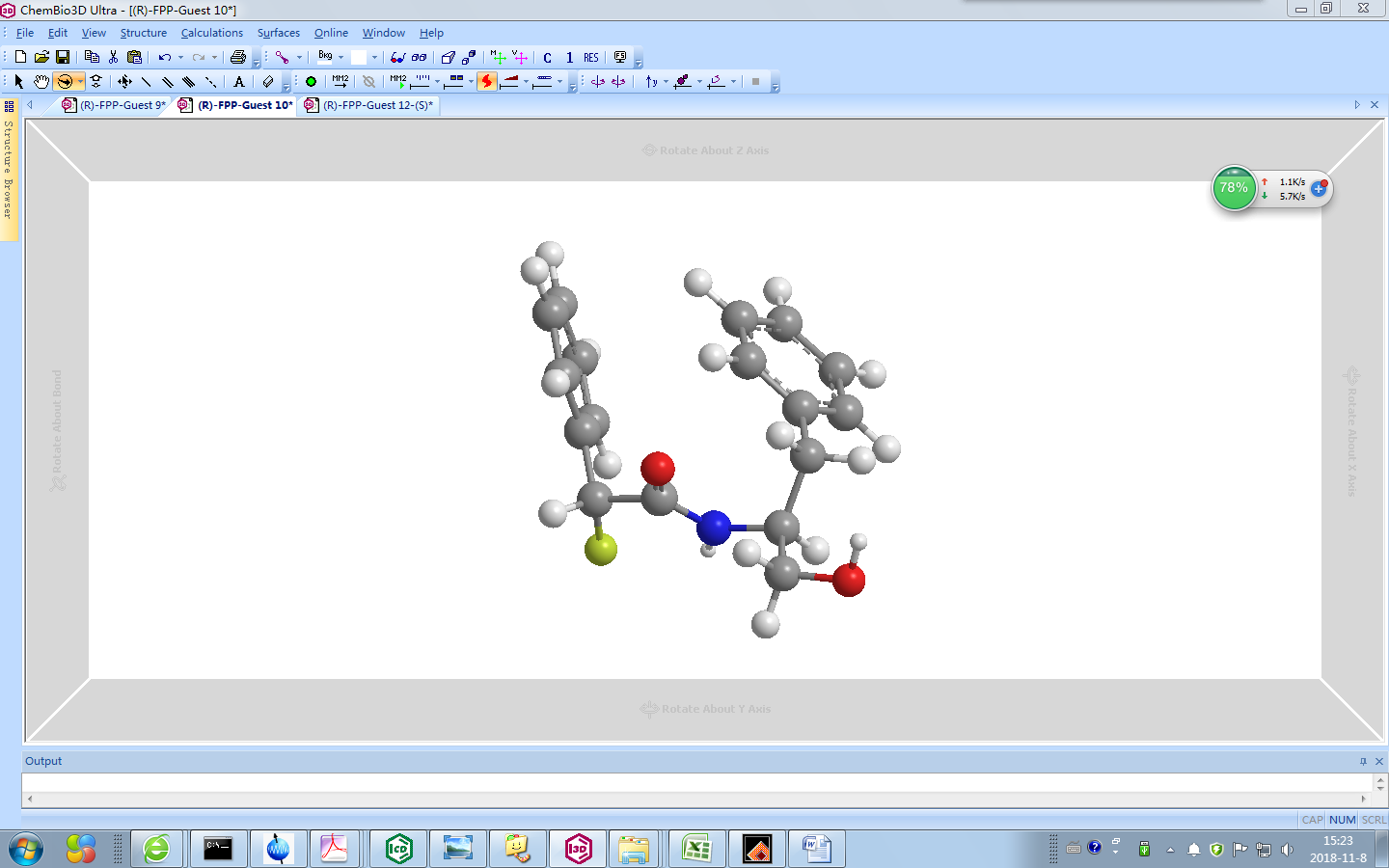
** δ_α-F_*^R^* _(calcd.)_ = -188.41 ppm

C -3.581680 0.409610 -0.864840

C -4.080550 0.423100 0.428980

C -3.507320 -0.392710 1.398100

C -2.449270 -1.226440 1.069980

C -1.954030 -1.244000 -0.231030

C -2.516940 -0.419650 -1.196460

C -0.799070 -2.152960 -0.574150

C 0.495500 -1.658650 0.076050

F -0.641450 -2.223220 -1.948560

N 1.352670 -1.046450 -0.750830

O 0.659380 -1.820140 1.274180

C 2.594130 -0.407420 -0.325770

C 3.612560 -1.473170 0.083620

C 2.380620 0.637120 0.780970

C 1.278380 1.606120 0.450970

C 0.019950 1.473180 1.031350

C -1.013050 2.337240 0.692020

C -0.800020 3.347180 -0.236300

C 0.453220 3.490700 -0.819920

C 1.483140 2.625670 -0.477160

O 4.876180 -0.904740 0.368550

H -4.019700 1.046220 -1.622690

H -4.911510 1.067810 0.684750

H -3.891200 -0.385860 2.410050

H -2.001760 -1.865630 1.820710

H -2.131610 -0.431080 -2.206770

H -0.977110 -3.165180 -0.208500

H 1.086660 -0.980170 -1.720210

H 2.979770 0.106170 -1.208580

H 3.755600 -2.163530 -0.748170

H 3.224410 -2.036150 0.936010

H 3.323100 1.175290 0.898930

H 2.158310 0.125630 1.717810

H -0.152140 0.678800 1.748820

H -1.986320 2.212080 1.148700

H -1.604290 4.021020 -0.502600

H 0.629570 4.280370 -1.539370

H 2.461410 2.745450 -0.930370

H 4.876560 -0.575330 1.272050

**Ball-cylinder model for (*S*)-FPA-amide 10 and cartesian coordinates (Gaussian 09).**


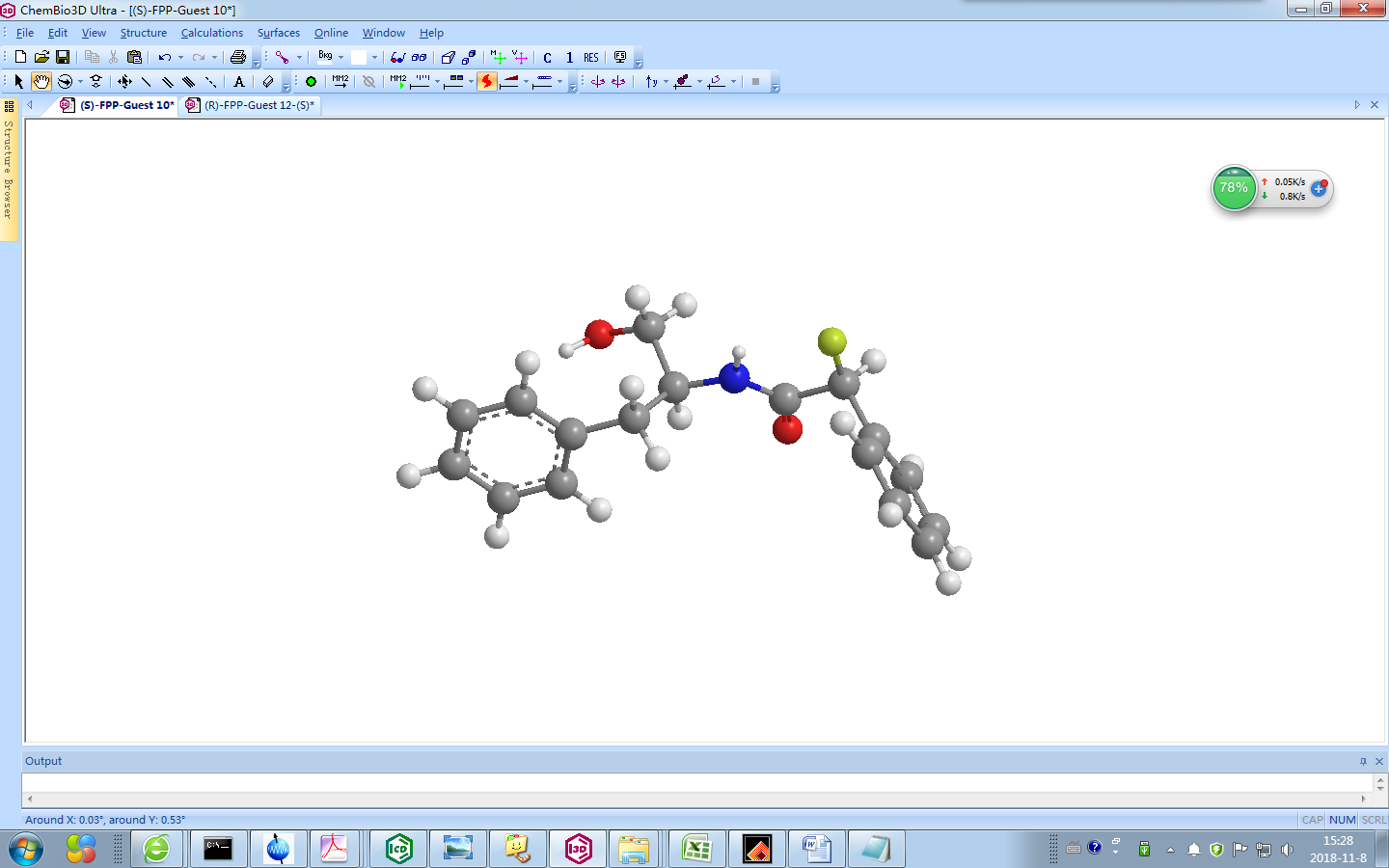
 δ_α-F_*^S^* _(calcd.)_ = -191.64 ppm

C -2.034993 -3.949075 -3.295647

C -2.281753 -4.820615 -2.235357

C -1.416083 -4.842805 -1.143917

C -0.310113 -3.995455 -1.107577

C -0.061023 -3.123775 -2.169567

C -0.927443 -3.105725 -3.266097

C 1.123907 -2.185075 -2.131757

C 0.712087 -0.752575 -1.740787

F 2.091437 -2.668215 -1.229247

N 1.181457 -0.325865 -0.548887

O 0.003637 -0.106235 -2.498587

C 0.908037 1.001295 -0.000097

C 2.203077 1.839115 -0.009037

C 0.260087 0.840655 1.396193

C -0.156723 2.132995 2.067343

C 0.573867 2.661315 3.139013

C 0.190517 3.857815 3.748063

C -0.934673 4.542055 3.296123

C -1.675693 4.021885 2.234113

C -1.290223 2.830185 1.627523

O 1.972997 3.234035 0.131183

H -2.701663 -3.929465 -4.150217

H -3.141083 -5.480895 -2.262437

H -1.599213 -5.520785 -0.317977

H 0.366437 -4.019615 -0.262927

H -0.738233 -2.428245 -4.089987

H 1.606207 -2.123965 -3.109577

H 1.783377 -0.954295 -0.036147

H 0.203007 1.475505 -0.684517

H 2.686167 1.709195 -0.979007

H 2.894337 1.465235 0.761163

H 0.961927 0.304165 2.044473

H -0.615793 0.196445 1.276153

H 1.445367 2.128615 3.506443

H 0.769287 4.248495 4.577473

H -1.235883 5.469935 3.768563

H -2.556553 4.545305 1.879583

H -1.875453 2.433315 0.804413

H 1.615387 3.395135 1.011543

**Ball-cylinder model for (*R*)-FPA-amide 11 and cartesian coordinates (Gaussian 09).**

**
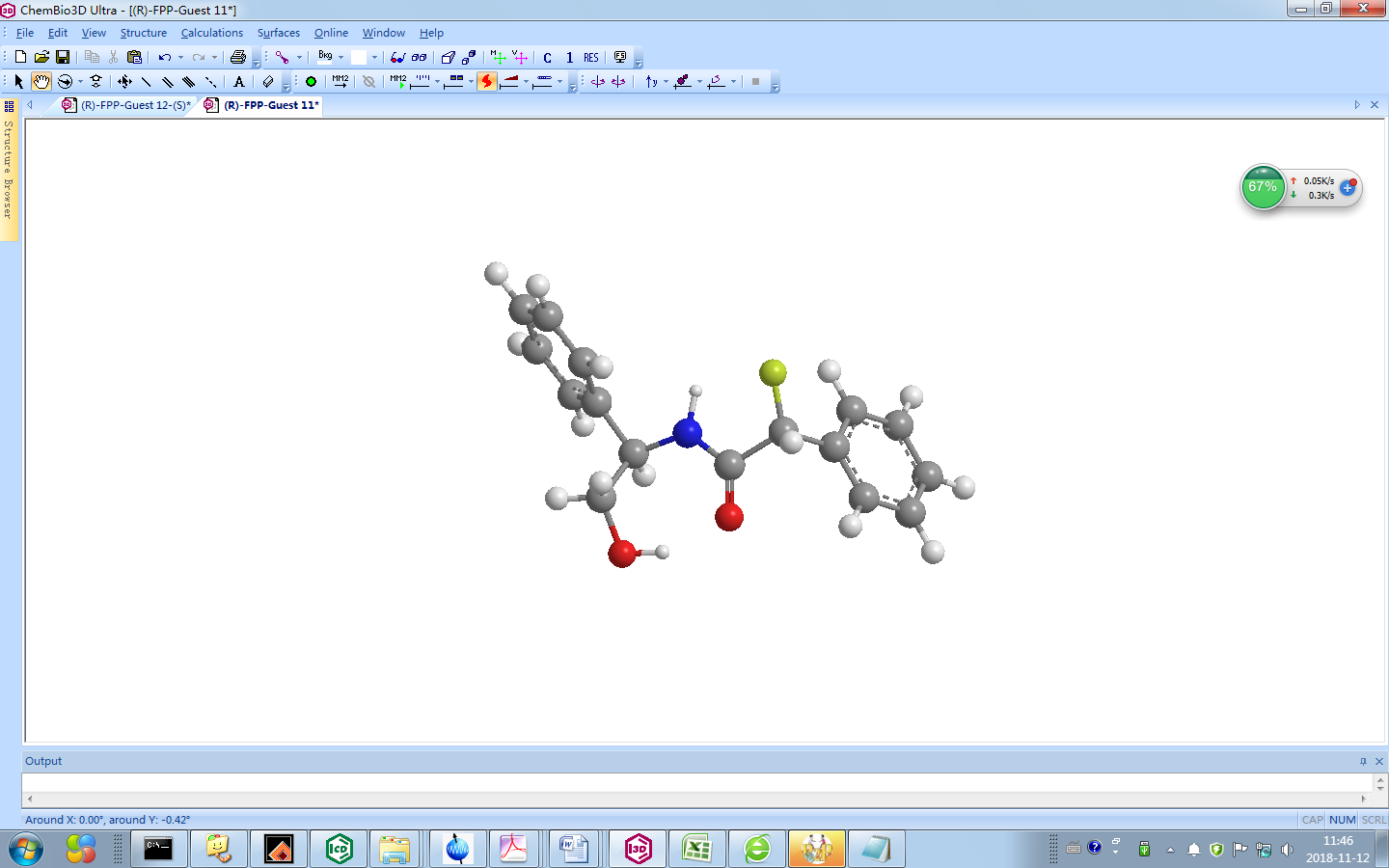
** δ_α-F_*^R^* _(calcd.)_ = -187.20 ppm

C 4.102762 -1.924683 0.977068

C 5.109066 -0.984596 1.187988

C 5.027108 0.265458 0.575401

C 3.945411 0.573577 -0.245262

C 2.932668 -0.366906 -0.452850

C 3.014852 -1.618015 0.161526

C 1.747248 -0.015345 -1.324960

C 0.731021 0.868294 -0.578721

F 1.117153 -1.187435 -1.779278

N -0.460496 0.300535 -0.341861

O 1.075062 2.007877 -0.265211

C -1.573146 0.849344 0.465340

C -1.955777 2.304901 0.092848

C -2.766639 -0.076889 0.324316

O -1.122744 3.279985 0.682656

C -3.427265 -0.548660 1.462225

C -4.554703 -1.359054 1.344094

C -5.034771 -1.710409 0.083812

C -4.381873 -1.246740 -1.057077

C -3.256314 -0.434107 -0.938348

H 4.162244 -2.900063 1.446412

H 5.954548 -1.225137 1.822283

H 5.808805 1.000089 0.731143

H 3.882340 1.546054 -0.718427

H 2.238579 -2.353039 -0.008455

H 2.062952 0.539539 -2.210870

H -0.561099 -0.655576 -0.653237

H -1.262583 0.873222 1.514813

H -1.992507 2.394564 -1.002765

H -2.962310 2.484312 0.475338

H -0.214132 3.059263 0.407142

H -3.055489 -0.281777 2.445827

H -5.053663 -1.719797 2.236359

H -5.909863 -2.343340 -0.008953

H -4.749121 -1.515581 -2.041012

H -2.756036 -0.079474 -1.833017

**Ball-cylinder model for (*S*)-FPA-amide 11 and cartesian coordinates (Gaussian 09).**


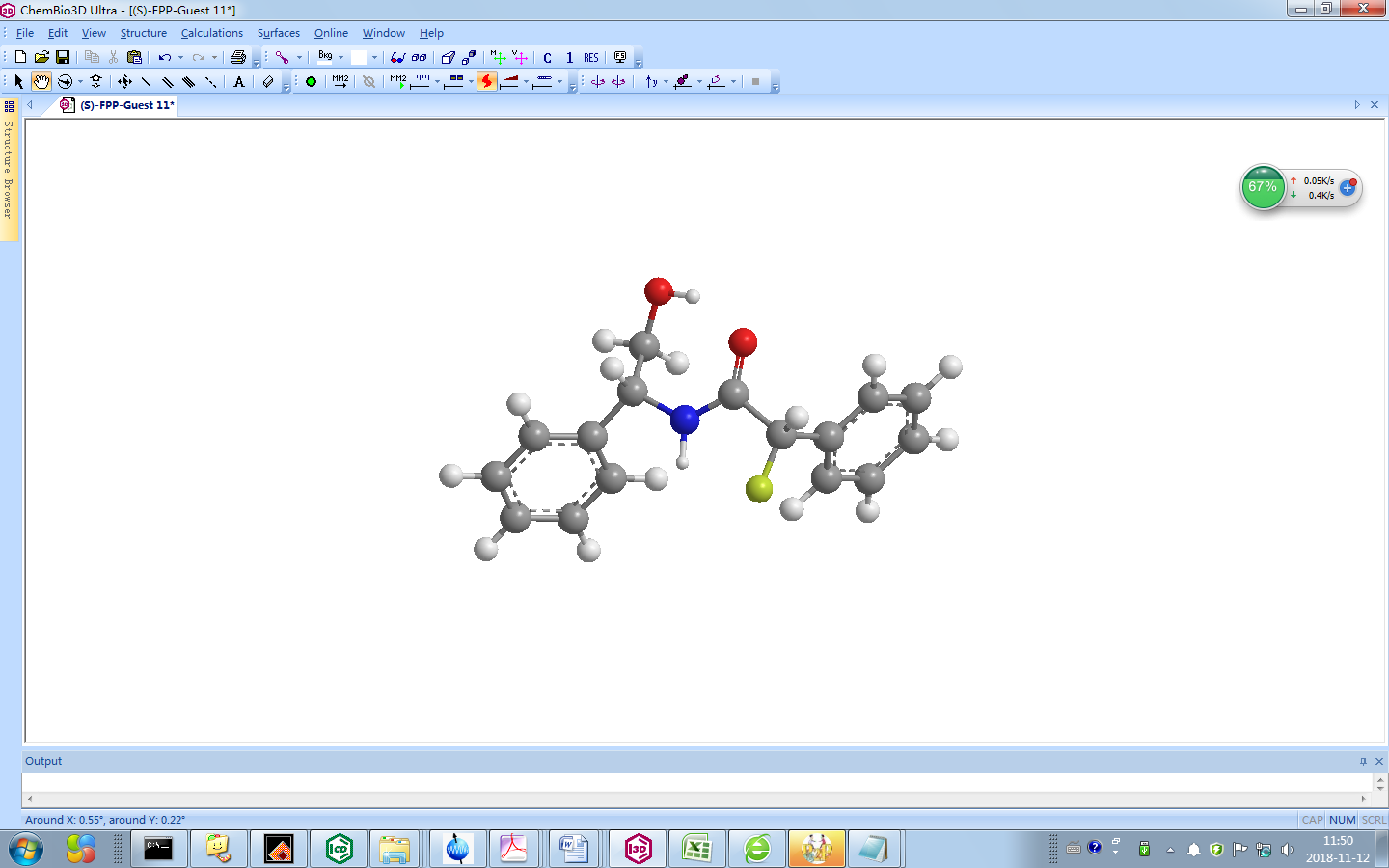
 δ_α-F_*^S^* _(calcd.)_ = -185.64 ppm

C 2.646382 1.670208 3.831396

C 3.618272 0.821258 3.318366

C 3.245132 -0.277462 2.555256

C 1.903672 -0.525112 2.299656

C 0.931372 0.324118 2.816406

C 1.303942 1.421118 3.583476

C -0.521658 0.087538 2.499636

C -0.904188 0.833108 1.220646

F -0.762318 -1.268972 2.346486

N -1.096358 0.069328 0.144416

O -0.970368 2.055528 1.258906

C -1.489698 0.534098 -1.191154

C -0.628268 1.703598 -1.708564

C -1.390328 -0.633592 -2.144014

O -1.069928 2.967658 -1.288784

C -2.426628 -0.903942 -3.030944

C -2.315798 -1.940092 -3.948324

C -1.165598 -2.716502 -3.986264

C -0.125878 -2.450982 -3.105054

C -0.236628 -1.413542 -2.189414

H 2.932872 2.524858 4.430236

H 4.664872 1.012778 3.516636

H 4.000032 -0.943422 2.157656

H 1.611102 -1.383862 1.708486

H 0.543382 2.081218 3.982016

H -1.166698 0.456868 3.297676

H -1.083148 -0.929062 0.293556

H -2.524078 0.887648 -1.155524

H 0.416832 1.516558 -1.428424

H -0.689668 1.698448 -2.797024

H -1.070548 2.949078 -0.317354

H -3.326308 -0.300542 -3.000324

H -3.131108 -2.143182 -4.630634

H -1.078828 -3.525702 -4.699684

H 0.774802 -3.050762 -3.131984

H 0.581372 -1.208282 -1.507454

**Ball-cylinder model for (*R*)-FPA-amide 12 and cartesian coordinates (Gaussian 09).**

**
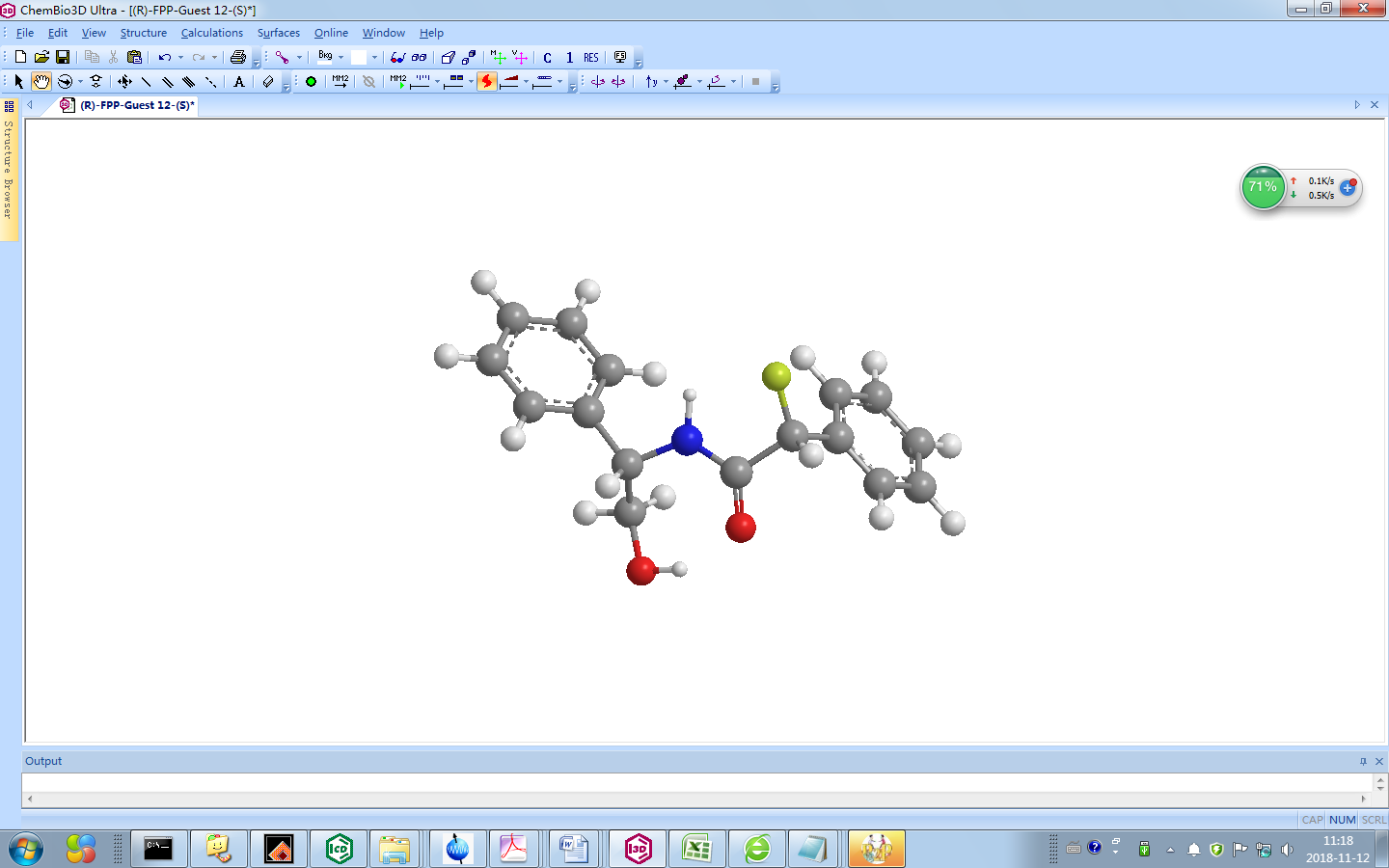
** δ_α-F_*^R^* _(calcd.)_ = -185.64 ppm

C 3.48922 1.09621 -3.33836

C 4.46111 0.24726 -2.82533

C 4.08797 -0.85146 -2.06222

C 2.74651 -1.09911 -1.80662

C 1.77421 -0.24988 -2.32337

C 2.14678 0.84712 -3.09044

C 0.32118 -0.48646 -2.0066

C -0.06135 0.25911 -0.72761

F 0.08052 -1.84297 -1.85345

N -0.25352 -0.50467 0.34862

O -0.12753 1.48153 -0.76587

C -0.64686 -0.0399 1.68419

C 0.21457 1.1296 2.2016

C -0.54749 -1.20759 2.63705

O -0.22709 2.39366 1.78182

C -1.58379 -1.47794 3.52398

C -1.47296 -2.51409 4.44136

C -0.32276 -3.2905 4.4793

C 0.71696 -3.02498 3.59809

C 0.60621 -1.98754 2.68245

H 3.77571 1.95086 -3.9372

H 5.50771 0.43878 -3.0236

H 4.84287 -1.51742 -1.66462

H 2.45394 -1.95786 -1.21545

H 1.38622 1.50722 -3.48898

H -0.32386 -0.11713 -2.80464

H -0.24031 -1.50306 0.19948

H -1.68124 0.31365 1.64856

H 1.25967 0.94256 1.92146

H 0.15317 1.12445 3.29006

H -0.22771 2.37508 0.81039

H -2.48347 -0.87454 3.49336

H -2.28827 -2.71718 5.12367

H -0.23599 -4.0997 5.19272

H 1.61764 -3.62476 3.62502

H 1.42421 -1.78228 2.00049

**Ball-cylinder model for (*S*)-FPA-amide 12 and cartesian coordinates (Gaussian 09).**


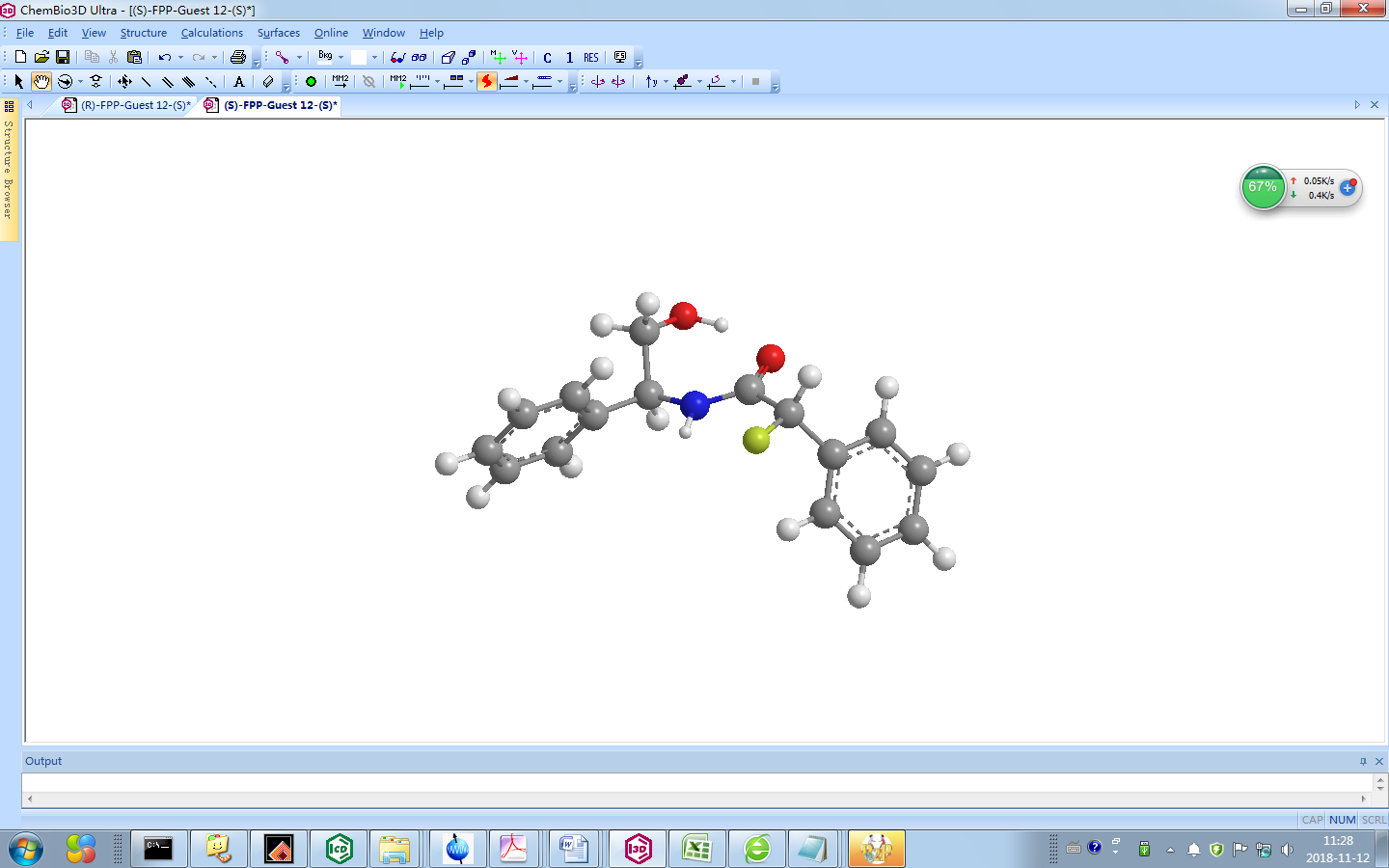
 δ_α-F_*^S^* _(calcd.)_ = -187.20 ppm

C 4.10276200 -1.92468300 -0.97706800

C 5.10906600 -0.98459600 -1.18798800

C 5.02710800 0.26545800 -0.57540100

C 3.94541100 0.57357700 0.24526200

C 2.93266800 -0.36690600 0.45285000

C 3.01485200 -1.61801500 -0.16152600

C 1.74724800 -0.01534500 1.32496000

C 0.73102100 0.86829400 0.57872100

F 1.11715300 -1.18743500 1.77927800

N -0.46049600 0.30053500 0.34186100

O 1.07506200 2.00787700 0.26521100

C -1.57314600 0.84934400 -0.46534000

C -1.95577700 2.30490100 -0.09284800

C -2.76663900 -0.07688900 -0.32431600

O -1.12274400 3.27998500 -0.68265600

C -3.42726500 -0.54866000 -1.46222500

C -4.55470300 -1.35905400 -1.34409400

C -5.03477100 -1.71040900 -0.08381200

C -4.38187300 -1.24674000 1.05707700

C -3.25631400 -0.43410700 0.93834800

H 4.16224400 -2.90006300 -1.44641200

H 5.95454800 -1.22513700 -1.82228300

H 5.80880500 1.00008900 -0.73114300

H 3.88234000 1.54605400 0.71842700

H 2.23857900 -2.35303900 0.00845500

H 2.06295200 0.53953900 2.21087000

H -0.56109900 -0.65557600 0.65323700

H -1.26258300 0.87322200 -1.51481300

H -1.99250700 2.39456400 1.00276500

H -2.96231000 2.48431200 -0.47533800

H -0.21413200 3.05926300 -0.40714200

H -3.05548900 -0.28177700 -2.44582700

H -5.05366300 -1.71979700 -2.23635900

H -5.90986300 -2.34334000 0.00895300

H -4.74912100 -1.51558100 2.04101200

H -2.75603600 -0.07947400 1.83301700

**Ball-cylinder model for (*R*)-FPA-amide 13 and cartesian coordinates (Gaussian 09).**

**
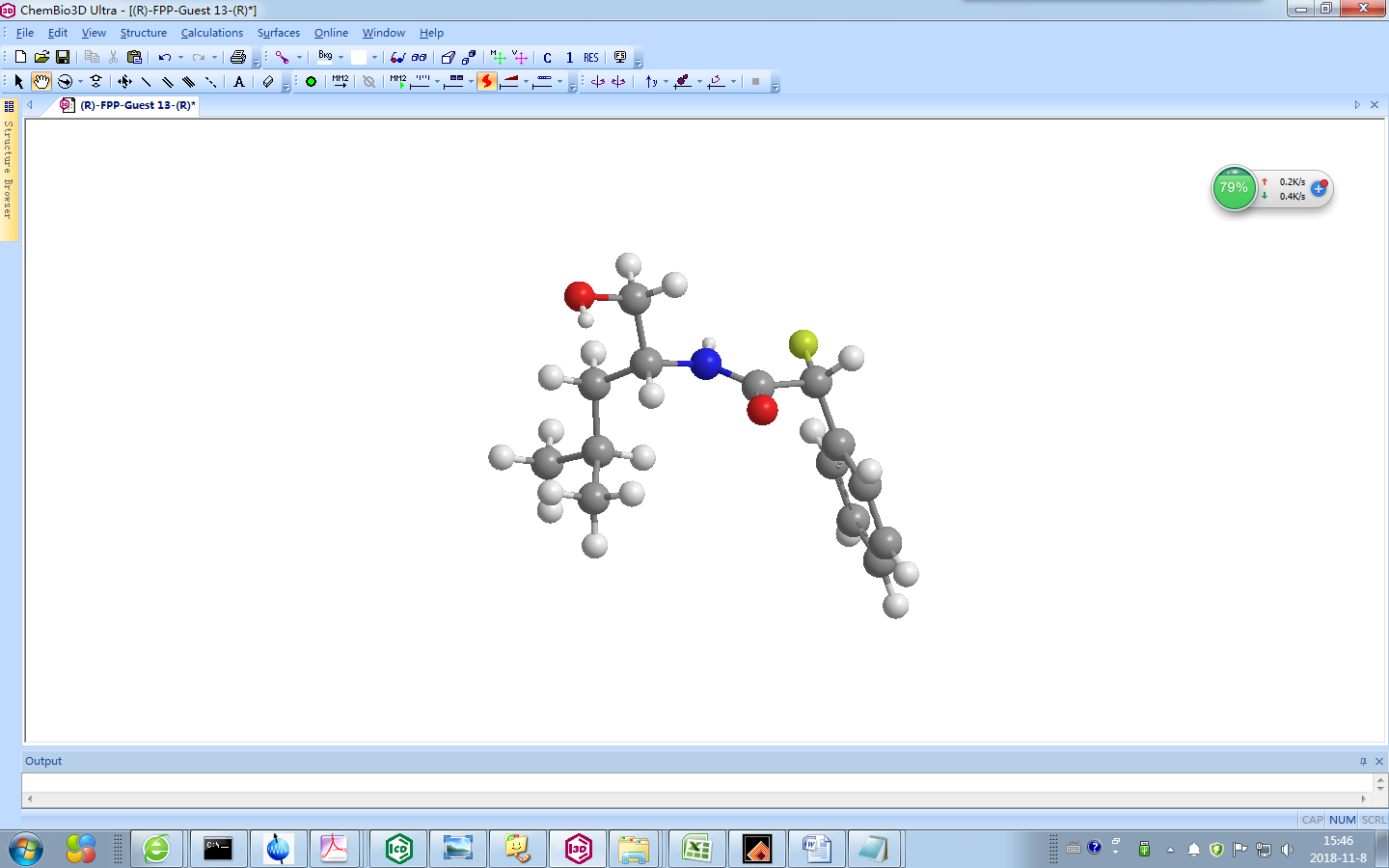
** δ_α-F_*^R^* _(calcd.)_ = -194.67 ppm

C 2.91589 0.82756 -3.74538

C 4.24512 1.01582 -3.36669

C 4.77421 0.27036 -2.31608

C 3.98077 -0.65794 -1.64273

C 2.65053 -0.84717 -2.02138

C 2.1212 -0.10112 -3.07932

C 1.77539 -1.84821 -1.29685

C 0.68758 -1.16448 -0.4474

F 2.56669 -2.67825 -0.48112

N 0.83175 -1.29434 0.88673

O -0.21385 -0.55374 -1.00673

C -0.1033 -0.72816 1.8596

C -1.06265 -1.82923 2.35258

C 0.64483 -0.05469 3.01496

C 1.49354 1.17517 2.64349

O -2.06324 -1.32819 3.23268

C 0.64309 2.32628 2.08703

C 2.30357 1.63957 3.8621

H 2.49726 1.40149 -4.56429

H 4.86362 1.73619 -3.88981

H 5.80727 0.40758 -2.01713

H 4.39691 -1.24234 -0.83258

H 1.08726 -0.24074 -3.3695

H 1.26272 -2.50205 -2.00605

H 1.60171 -1.8574 1.21967

H -0.69571 0.00652 1.30964

H -1.5093 -2.32746 1.48397

H -0.50576 -2.57873 2.92201

H -0.10077 0.22834 3.76492

H 1.28931 -0.80608 3.49099

H 2.20424 0.87407 1.86397

H -2.65839 -0.77186 2.71849

H 1.26853 3.19547 1.86467

H 0.12878 2.0504 1.16326

H -0.11369 2.63818 2.81567

H 2.94037 2.49226 3.60999

H 2.94721 0.84045 4.24193

H 1.63956 1.94843 4.67693

**Ball-cylinder model for (*S*)-FPA-amide 13 and cartesian coordinates (Gaussian 09).**


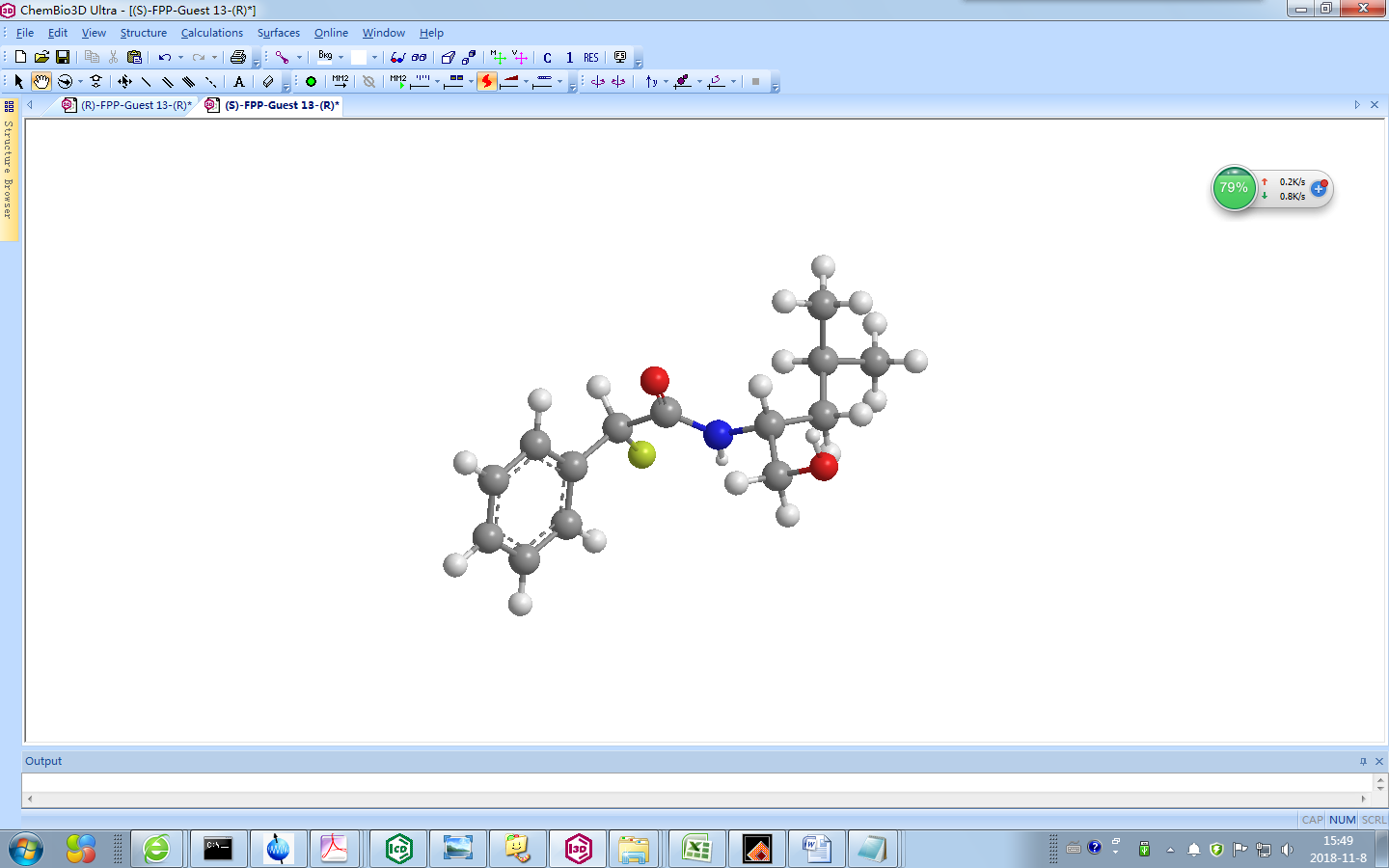
 δ_α-F_*^S^* _(calcd.)_ = -194.01 ppm

C 5.165 -1.15718 -2.69791

C 4.74651 -2.47942 -2.82382

C 3.39947 -2.79632 -2.64815

C 2.47679 -1.7977 -2.34987

C 2.89715 -0.47011 -2.2184

C 4.24516 -0.1538 -2.39474

C 1.88729 0.60788 -1.88321

C 1.34012 0.46938 -0.45044

F 2.46625 1.87947 -2.05189

N 1.70445 1.44683 0.40301

O 0.62523 -0.48414 -0.17021

C 1.28538 1.49235 1.80447

C 2.38691 0.88428 2.69488

C 0.9316 2.92329 2.2214

C -0.28547 3.5498 1.51578

O 1.99614 0.80282 4.06185

C -1.59312 2.81432 1.84289

C -0.396 5.037 1.88083

H 6.20938 -0.90121 -2.83649

H 5.46277 -3.25796 -3.06043

H 3.06467 -3.82262 -2.74756

H 1.43277 -2.04573 -2.20488

H 4.57172 0.87397 -2.30562

H 1.02928 0.56134 -2.55771

H 2.28734 2.18898 0.04266

H 0.40863 0.84457 1.87783

H 2.66194 -0.1006 2.29998

H 3.27704 1.51895 2.66944

H 0.76533 2.91645 3.30321

H 1.8121 3.55745 2.0506

H -0.12138 3.48293 0.4329

H 1.30797 0.13235 4.13328

H -2.44104 3.29443 1.34622

H -1.57386 1.77087 1.51897

H -1.78679 2.82915 2.92121

H -1.23652 5.50855 1.36369

H 0.51294 5.58295 1.61108

H -0.55381 5.16348 2.95752

**Ball-cylinder model for (*R*)-FPA-amide 14 and cartesian coordinates (Gaussian 09).**

**
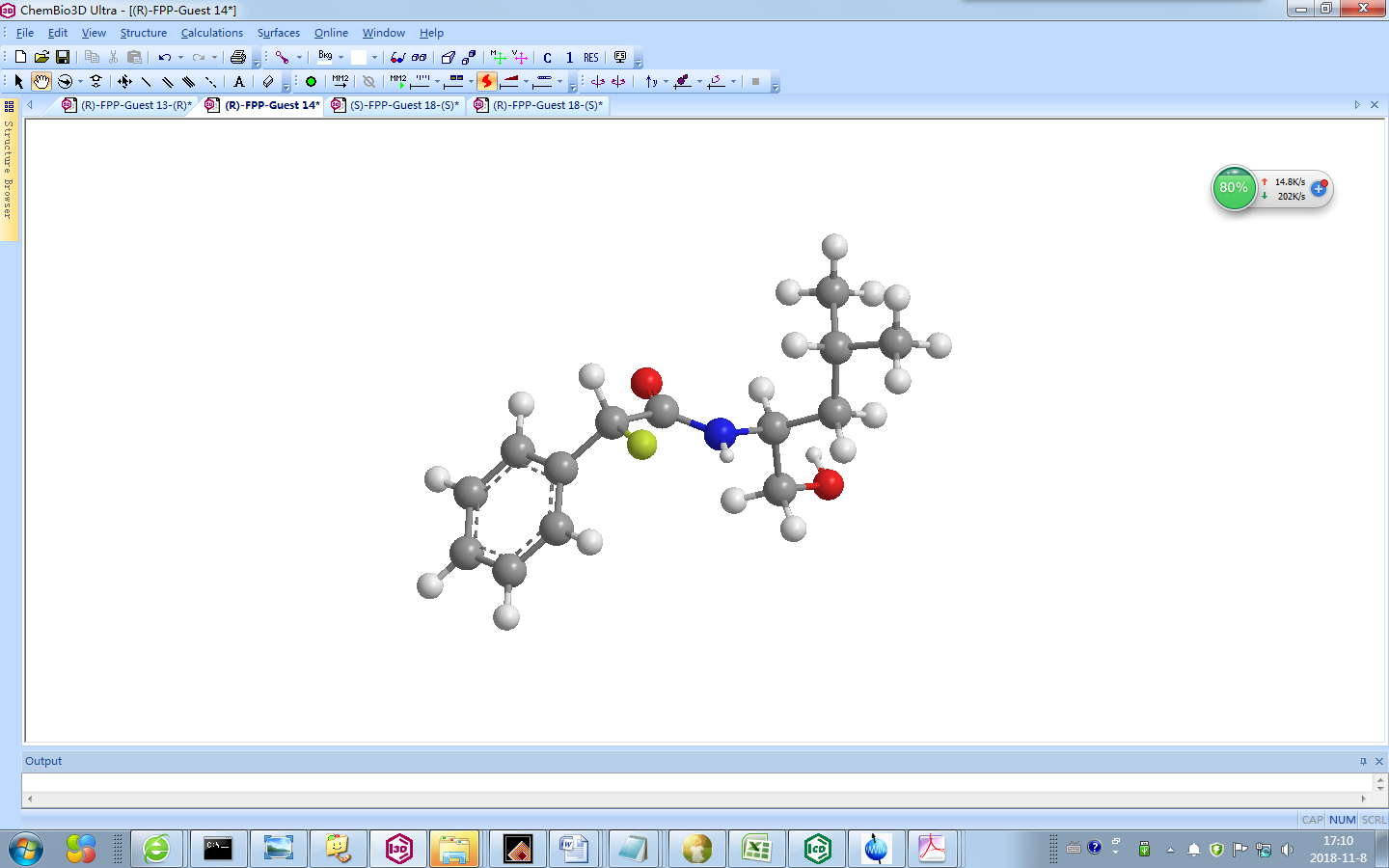
** δ_α-F_*^R^* _(calcd.)_ = -194.01 ppm

C 3.523702 -2.279997 2.949288

C 3.105212 -3.602237 3.075198

C 1.758172 -3.919137 2.899528

C 0.835492 -2.920517 2.601248

C 1.255852 -1.592927 2.469778

C 2.603862 -1.276617 2.646118

C 0.245992 -0.514937 2.134588

C -0.301178 -0.653437 0.701818

F 0.824952 0.756653 2.303268

N 0.063152 0.324013 -0.151632

O -1.016068 -1.606957 0.421588

C -0.355918 0.369533 -1.553092

C 0.745612 -0.238537 -2.443502

C -0.709698 1.800473 -1.970022

C -1.926768 2.426983 -1.264402

O 0.354842 -0.319997 -3.810472

C -3.234418 1.691503 -1.591512

C -2.037298 3.914183 -1.629452

H 4.568082 -2.024027 3.087868

H 3.821472 -4.380777 3.311808

H 1.423372 -4.945437 2.998938

H -0.208528 -3.168547 2.456258

H 2.930422 -0.248847 2.556998

H -0.612018 -0.561477 2.809088

H 0.646042 1.066163 0.208718

H -1.232668 -0.278247 -1.626452

H 1.020642 -1.223417 -2.048602

H 1.635742 0.396133 -2.418062

H -0.875968 1.793633 -3.051832

H 0.170802 2.434633 -1.799222

H -1.762678 2.360113 -0.181522

H -0.333328 -0.990467 -3.881902

H -4.082338 2.171613 -1.094842

H -3.215158 0.648053 -1.267592

H -3.428088 1.706333 -2.669832

H -2.877818 4.385733 -1.112312

H -1.128358 4.460133 -1.359702

H -2.195108 4.040663 -2.706142

**Ball-cylinder model for (*S*)-FPA-amide 14 and cartesian coordinates (Gaussian 09).**


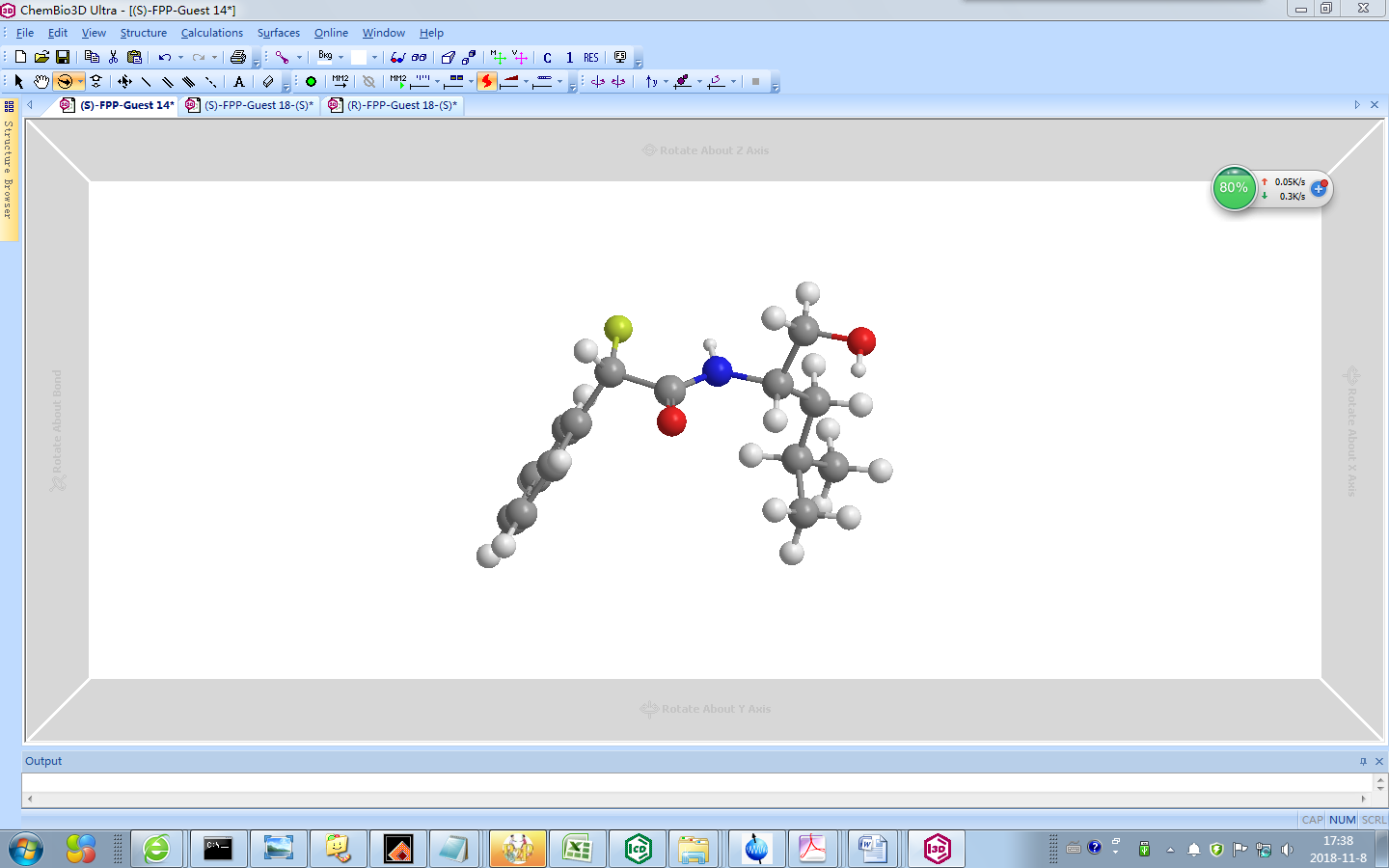
 δ_α-F_*^S^* _(calcd.)_ = -194.67 ppm

C 1.427936 0.836456 4.298071

C 2.757166 1.024716 3.919381

C 3.286256 0.279256 2.868771

C 2.492816 -0.649044 2.195421

C 1.162576 -0.838274 2.574071

C 0.633246 -0.092224 3.632011

C 0.287436 -1.839314 1.849541

C -0.800374 -1.155584 1.000091

F 1.078736 -2.669354 1.033811

N -0.656204 -1.285444 -0.334039

O -1.701804 -0.544844 1.559421

C -1.591254 -0.719264 -1.306909

C -2.550604 -1.820334 -1.799889

C -0.843124 -0.045794 -2.462269

C 0.005586 1.184066 -2.090799

O -3.551194 -1.319294 -2.679989

C -0.844864 2.335176 -1.534339

C 0.815616 1.648466 -3.309409

H 1.009306 1.410386 5.116981

H 3.375666 1.745086 4.442501

H 4.319316 0.416476 2.569821

H 2.908956 -1.233444 1.385271

H -0.400694 -0.231844 3.922191

H -0.225234 -2.493154 2.558741

H 0.113756 -1.848504 -0.666979

H -2.183664 0.015416 -0.756949

H -2.997254 -2.318564 -0.931279

H -1.993714 -2.569834 -2.369319

H -1.588724 0.237236 -3.212229

H -0.198644 -0.797184 -2.938299

H 0.716286 0.882966 -1.311279

H -4.146344 -0.762964 -2.165799

H -0.219424 3.204366 -1.311979

H -1.359174 2.059296 -0.610569

H -1.601644 2.647076 -2.262979

H 1.452416 2.501156 -3.057299

H 1.459256 0.849346 -3.689239

H 0.151606 1.957326 -4.124239

**Ball-cylinder model for (*R*)-FPA-amide 15 and cartesian coordinates (Gaussian 09).**

**
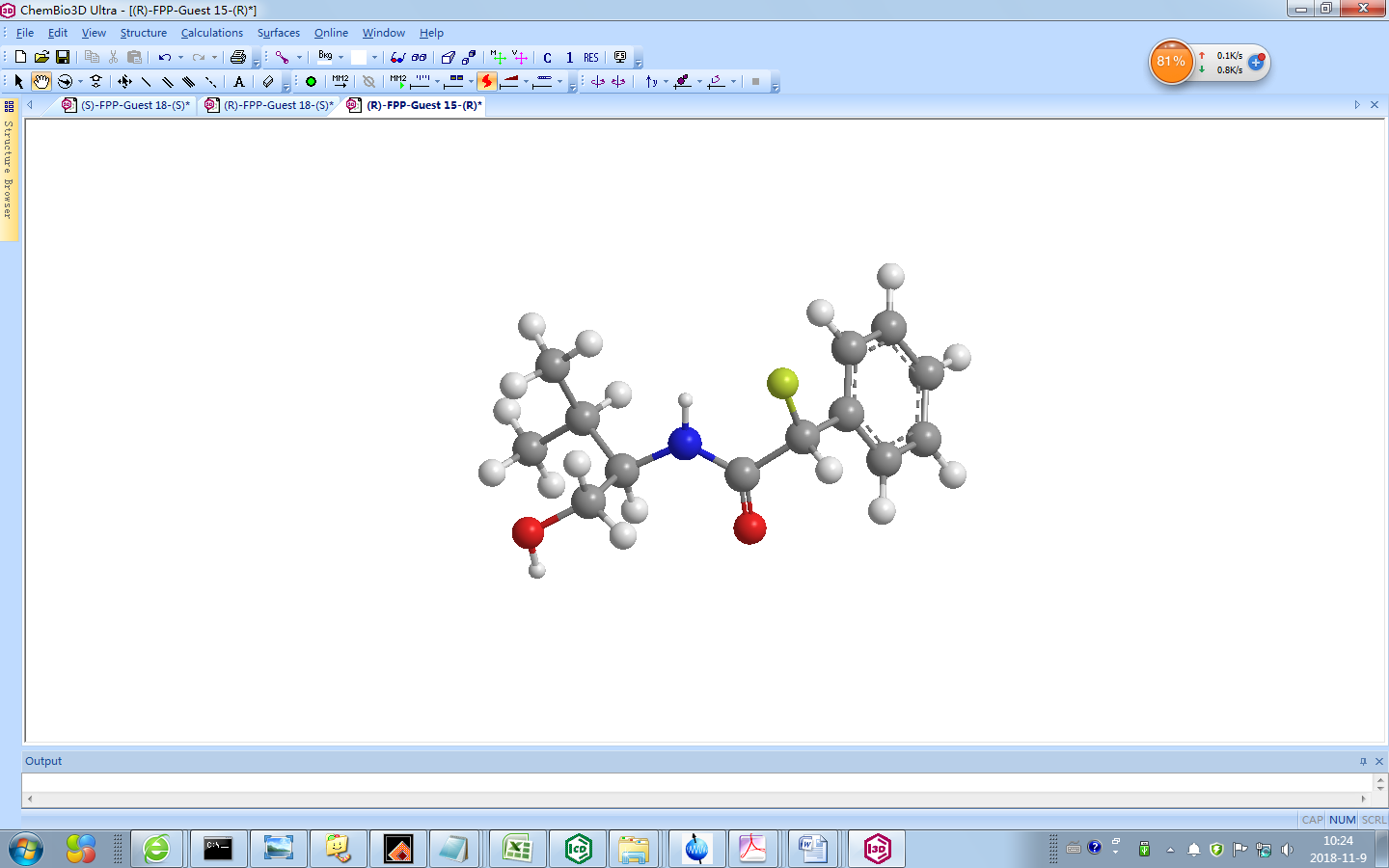
** δ_α-F_*^R^* _(calcd.)_ = -194.34 ppm

C 4.67178 -0.16082 -2.34577

C 4.90725 -1.5355 -2.32888

C 3.83524 -2.41725 -2.2142

C 2.53229 -1.93106 -2.11281

C 2.29576 -0.55536 -2.13022

C 3.37225 0.32894 -2.25028

C 0.88929 -0.00983 -2.00646

C 0.63717 0.62637 -0.6263

F -0.04779 -1.03358 -2.23981

N -0.21915 -0.04523 0.16926

O 1.21586 1.66367 -0.33116

C -0.57817 0.3952 1.52118

C -1.93753 1.11966 1.44001

C -0.48834 -0.79598 2.51084

C -0.34078 -0.33521 3.96902

O -2.38265 1.66122 2.67925

C -1.64519 -1.79573 2.36428

H 5.50053 0.53189 -2.43828

H 5.91936 -1.91555 -2.40843

H 4.00931 -3.4873 -2.20418

H 1.70022 -2.61844 -2.03231

H 3.19089 1.39666 -2.25673

H 0.70303 0.7636 -2.75467

H -0.66123 -0.8676 -0.21504

H 0.18349 1.12952 1.79705

H -1.86033 1.90071 0.67397

H -2.71513 0.42243 1.12052

H 0.43884 -1.31507 2.24014

H -0.16269 -1.19606 4.61975

H 0.50666 0.34779 4.08139

H -1.23967 0.17705 4.31365

H -1.77605 2.36545 2.93279

H -1.45465 -2.68406 2.97195

H -1.77878 -2.13464 1.33192

H -2.59077 -1.36204 2.7002

**Ball-cylinder model for (*S*)-FPA-amide 15 and cartesian coordinates (Gaussian 09).**


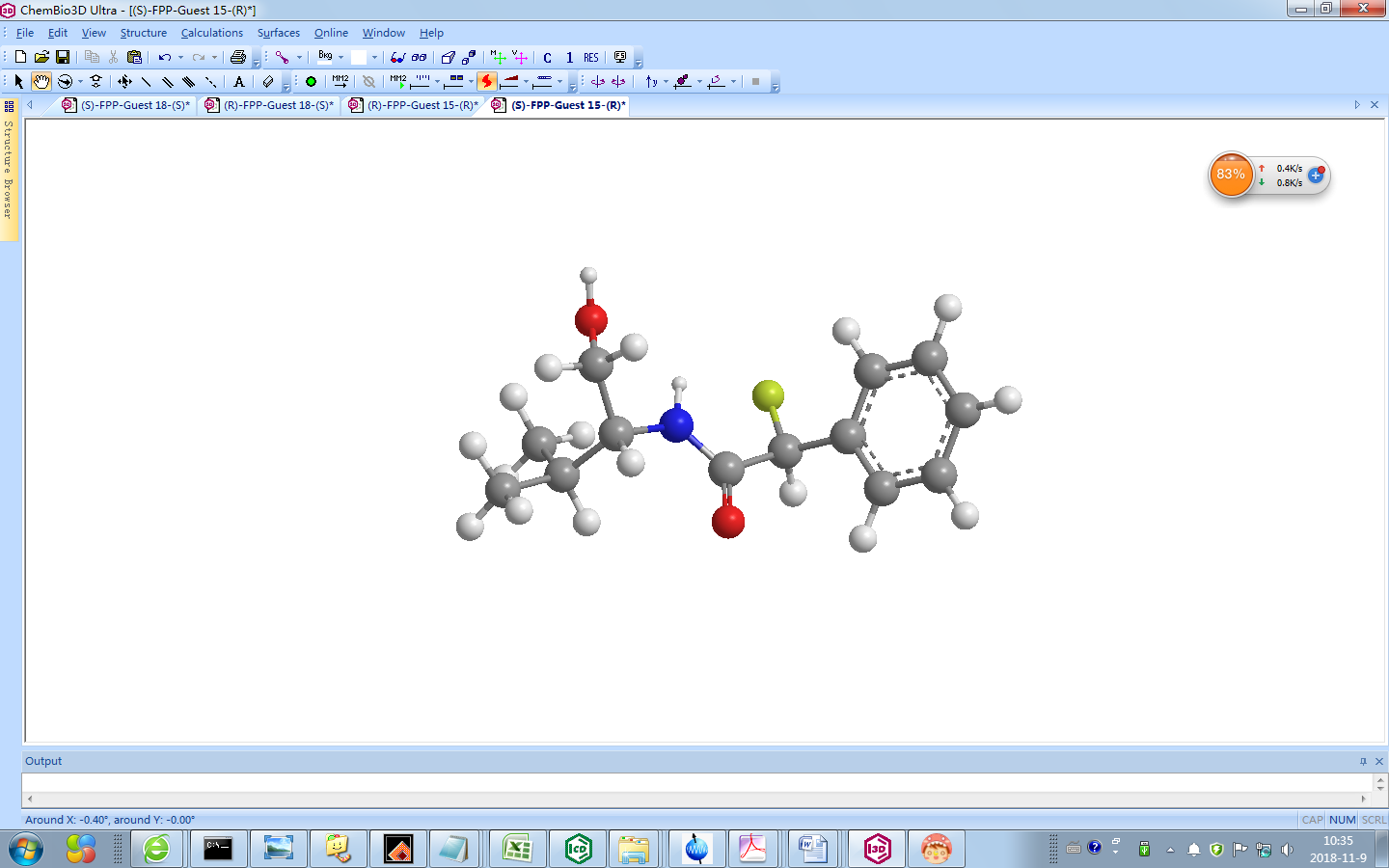
 δ_α-F_*^S^* _(calcd.)_ = -193.45 ppm

C 4.04522 -1.82656 -2.58484

C 3.57385 -2.4072 -3.75979

C 2.22049 -2.3094 -4.08306

C 1.3445 -1.63451 -3.23754

C 1.81591 -1.05537 -2.05498

C 3.17055 -1.15349 -1.73256

C 0.85166 -0.3384 -1.1342

C -0.10022 -1.31412 -0.41594

F 1.56322 0.42016 -0.18646

N 0.07731 -1.41432 0.9121

O -0.93503 -1.92898 -1.06918

C -0.71849 -2.2904 1.77111

C 0.20991 -2.83983 2.85542

C -2.02402 -1.59766 2.251

C -2.92769 -2.57657 3.01309

O 0.87468 -1.74434 3.49369

C -1.80007 -0.30629 3.0495

H 5.09641 -1.89446 -2.32802

H 4.25551 -2.92918 -4.42169

H 1.84604 -2.75543 -4.99748

H 0.29242 -1.56598 -3.48483

H 3.54061 -0.69588 -0.82434

H 0.22841 0.36333 -1.69326

H 0.78531 -0.84779 1.35729

H -1.02487 -3.13829 1.15188

H -0.36483 -3.42832 3.57847

H 0.942 -3.50218 2.37919

H -2.545 -1.32911 1.32448

H -3.91408 -2.13363 3.17396

H -3.06891 -3.50989 2.45929

H -2.5184 -2.82466 3.99704

H 1.60007 -2.09657 4.01833

H -1.29476 -0.50425 3.99667

H -2.76023 0.17247 3.26322

H -1.19121 0.40787 2.48961

**Ball-cylinder model for (*R*)-FPA-amide 16 and cartesian coordinates (Gaussian 09).**

**
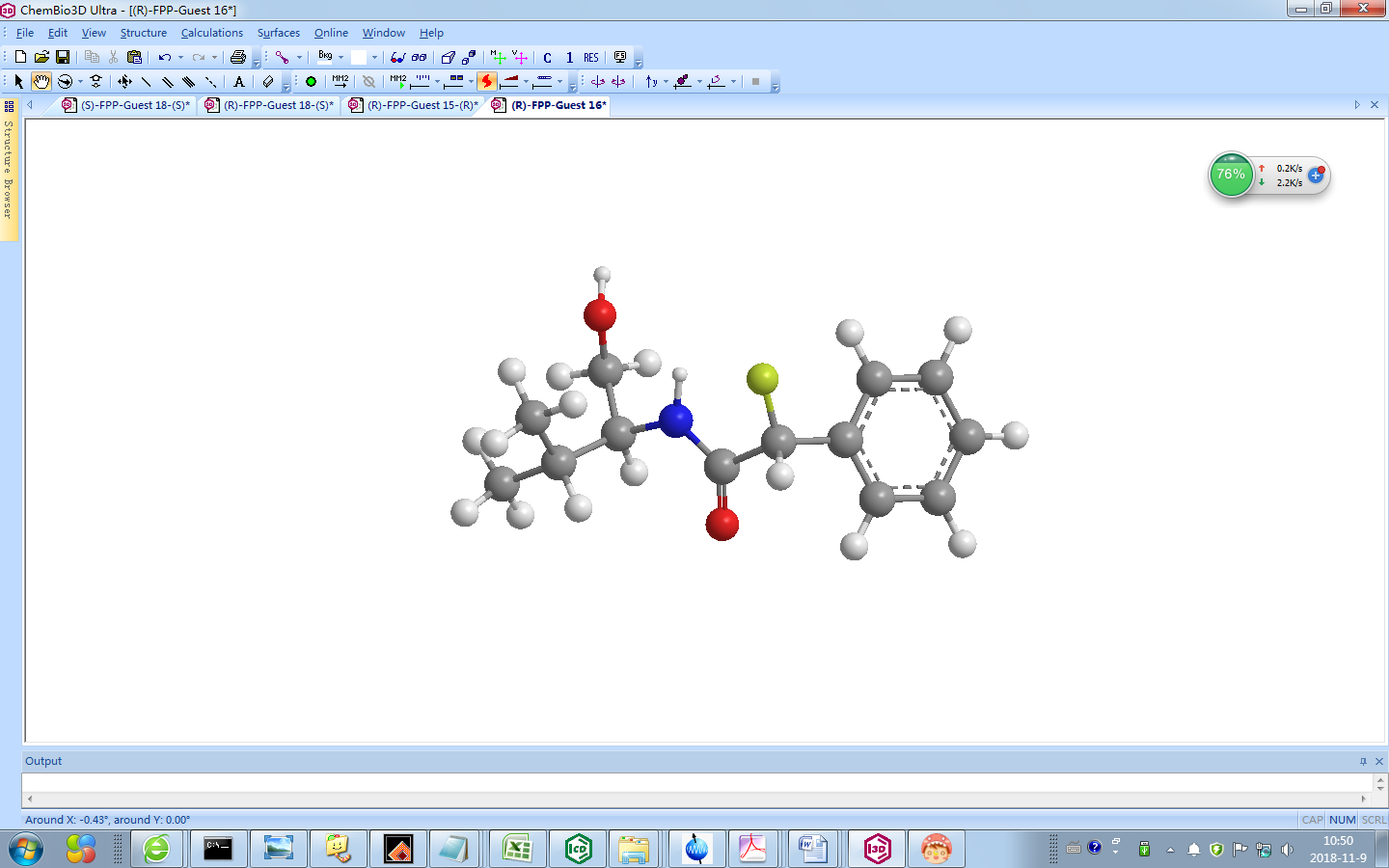
** δ_α-F_*^R^* _(calcd.)_ = -193.45 ppm

C 4.045220 -1.826560 2.584840

C 3.573850 -2.407200 3.759790

C 2.220490 -2.309400 4.083060

C 1.344500 -1.634510 3.237540

C 1.815910 -1.055370 2.054980

C 3.170550 -1.153490 1.732560

C 0.851660 -0.338400 1.134200

C -0.100220 -1.314120 0.415940

F 1.563220 0.420160 0.186460

N 0.077310 -1.414320 -0.912100

O -0.935030 -1.928980 1.069180

C -0.718490 -2.290400 -1.771110

C 0.209910 -2.839830 -2.855420

C -2.024020 -1.597660 -2.251000

C -2.927690 -2.576570 -3.013090

O 0.874680 -1.744340 -3.493690

C -1.800070 -0.306290 -3.049500

H 5.096410 -1.894460 2.328020

H 4.255510 -2.929180 4.421690

H 1.846040 -2.755430 4.997480

H 0.292420 -1.565980 3.484830

H 3.540610 -0.695880 0.824340

H 0.228410 0.363330 1.693260

H 0.785310 -0.847790 -1.357290

H -1.024870 -3.138290 -1.151880

H -0.364830 -3.428320 -3.578470

H 0.942000 -3.502180 -2.379190

H -2.545000 -1.329110 -1.324480

H -3.914080 -2.133630 -3.173960

H -3.068910 -3.509890 -2.459290

H -2.518400 -2.824660 -3.997040

H 1.600070 -2.096570 -4.018330

H -1.294760 -0.504250 -3.996670

H -2.760230 0.172470 -3.263220

H -1.191210 0.407870 -2.489610

**Ball-cylinder model for (*S*)-FPA-amide 16 and cartesian coordinates (Gaussian 09).**


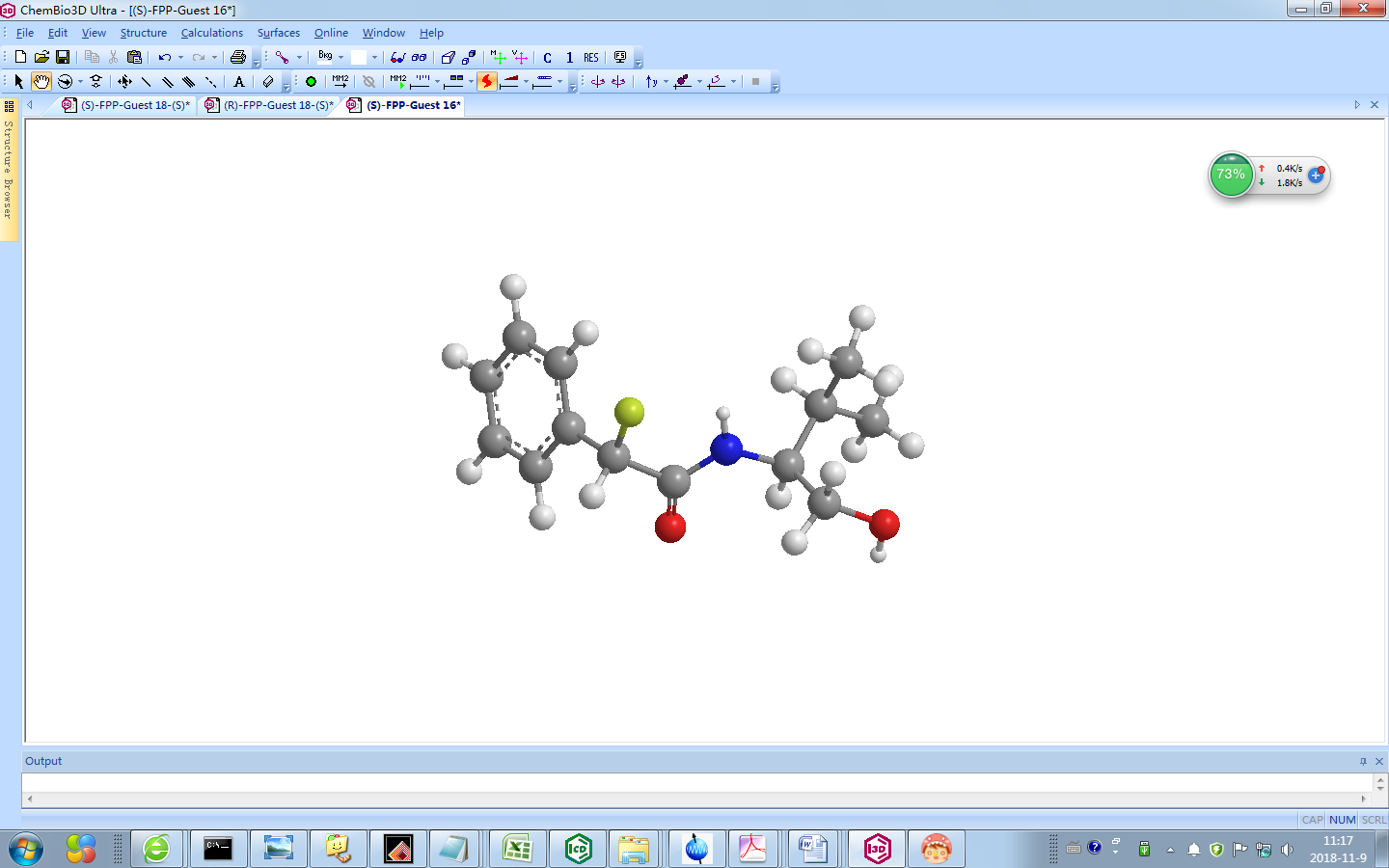
 δ_α-F_*^S^* _(calcd.)_ = -194.34 ppm

C 3.968057 0.221070 2.646960

C 4.203527 -1.153610 2.630070

C 3.131517 -2.035360 2.515390

C 1.828567 -1.549170 2.414000

C 1.592037 -0.173470 2.431410

C 2.668527 0.710830 2.551470

C 0.185567 0.372060 2.307650

C -0.066553 1.008260 0.927490

F -0.751513 -0.651690 2.541000

N -0.922873 0.336660 0.131930

O 0.512137 2.045560 0.632350

C -1.281893 0.777090 -1.219990

C -2.641253 1.501550 -1.138820

C -1.192063 -0.414090 -2.209650

C -1.044503 0.046680 -3.667830

O -3.086373 2.043110 -2.378060

C -2.348913 -1.413840 -2.063090

H 4.796807 0.913780 2.739470

H 5.215637 -1.533660 2.709620

H 3.305587 -3.105410 2.505370

H 0.996497 -2.236550 2.333500

H 2.487167 1.778550 2.557920

H -0.000693 1.145490 3.055860

H -1.364953 -0.485710 0.516230

H -0.520233 1.511410 -1.495860

H -2.564053 2.282600 -0.372780

H -3.418853 0.804320 -0.819330

H -0.264883 -0.933180 -1.938950

H -0.866413 -0.814170 -4.318560

H -0.197063 0.729680 -3.780200

H -1.943393 0.558940 -4.012460

H -2.479773 2.747340 -2.631600

H -2.158373 -2.302170 -2.670760

H -2.482503 -1.752750 -1.030730

H -3.294493 -0.980150 -2.399010

**Ball-cylinder model for (*R*)-FPA-amide 17 and cartesian coordinates (Gaussian 09).**

**
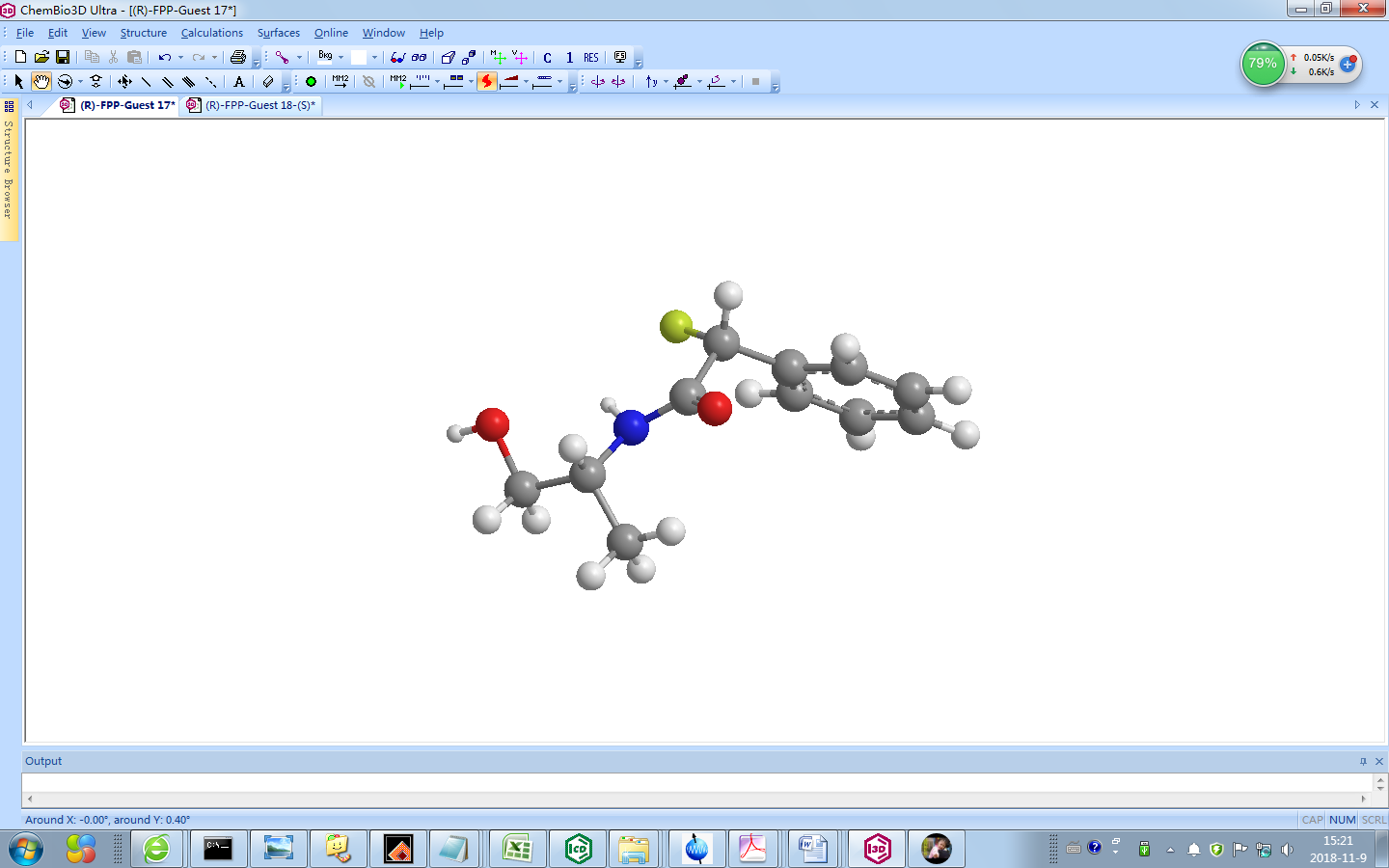
** δ_α-F_*^R^* _(calcd.)_ = -189.90 ppm

C 3.085543 -0.707293 2.091460

C 3.249553 -2.090613 2.067340

C 2.139733 -2.914663 1.882790

C 0.872693 -2.358813 1.726670

C 0.707443 -0.970983 1.746980

C 1.819713 -0.146963 1.928480

C -0.668347 -0.375133 1.548090

C -1.097037 -0.402613 0.067600

F -0.694967 0.946397 2.032880

N -1.139667 0.799337 -0.538730

O -1.345157 -1.479413 -0.459610

C -1.659197 1.000677 -1.896820

C -1.760567 2.503637 -2.135640

C -0.776657 0.340247 -2.958390

O -2.511107 3.078887 -1.065980

H 3.943133 -0.060273 2.237800

H 4.234653 -2.524723 2.195130

H 2.258803 -3.992063 1.866360

H 0.011753 -2.999783 1.579760

H 1.692083 0.927597 1.952820

H -1.420767 -0.933353 2.109120

H -1.135857 1.613207 0.061580

H -2.666587 0.570867 -1.948100

H -2.248337 2.673417 -3.102440

H -0.750907 2.933907 -2.176740

H -1.186627 0.523867 -3.955110

H -0.731187 -0.735283 -2.795330

H 0.239053 0.743247 -2.919370

H -2.461197 4.036687 -1.142610

**Ball-cylinder model for (*S*)-FPA-amide 17 and cartesian coordinates (Gaussian 09).**


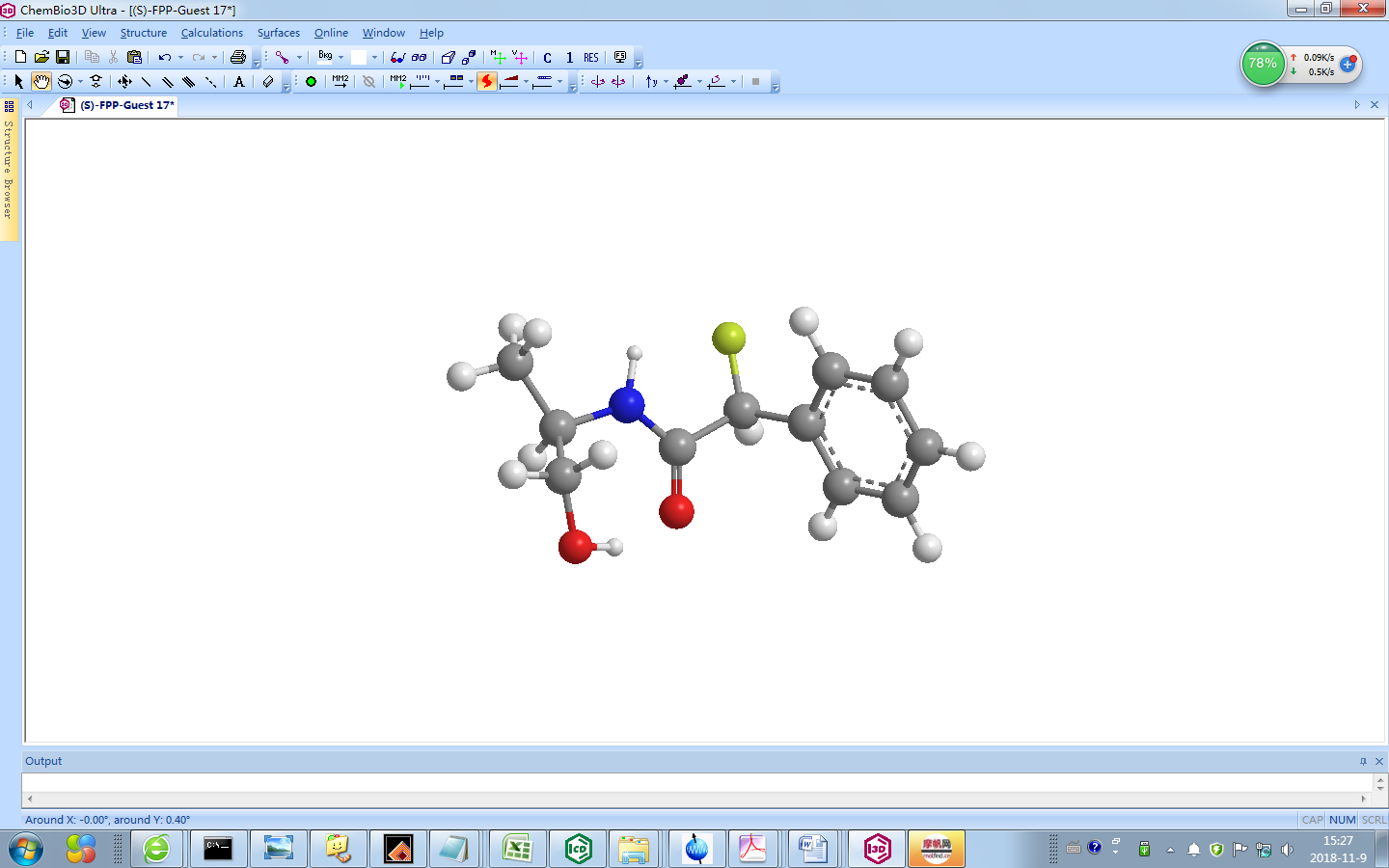
 δ_α-F_*^S^* _(calcd.)_ = -188.65 ppm

C 3.366783 -0.136593 2.084686

C 4.001743 -1.025203 1.217566

C 3.239683 -1.886883 0.431616

C 1.848043 -1.859733 0.506226

C 1.211983 -0.971503 1.375376

C 1.977183 -0.111003 2.167296

C -0.298257 -0.918233 1.452186

C -0.872997 0.280977 0.678976

F -0.849527 -2.113423 0.955816

N -1.608987 -0.018373 -0.399664

O -0.621877 1.415317 1.087426

C -2.357617 0.949027 -1.230474

C -1.446947 2.007457 -1.883004

C -3.138627 0.154487 -2.277144

O -1.135287 3.096737 -1.033244

H 3.953453 0.533917 2.702126

H 5.083873 -1.048013 1.158276

H 3.726473 -2.583393 -0.241564

H 1.256743 -2.534773 -0.099034

H 1.485743 0.582417 2.838906

H -0.633997 -0.818713 2.486796

H -1.757837 -1.001413 -0.579374

H -3.056197 1.486127 -0.579514

H -1.975957 2.430037 -2.740914

H -0.542137 1.507737 -2.260814

H -3.796717 0.818297 -2.839384

H -3.758237 -0.612593 -1.805924

H -2.461277 -0.334563 -2.983784

H -0.839217 2.711887 -0.189444

**Ball-cylinder model for (*R*)-FPA-amide 18 and cartesian coordinates (Gaussian 09).**

**
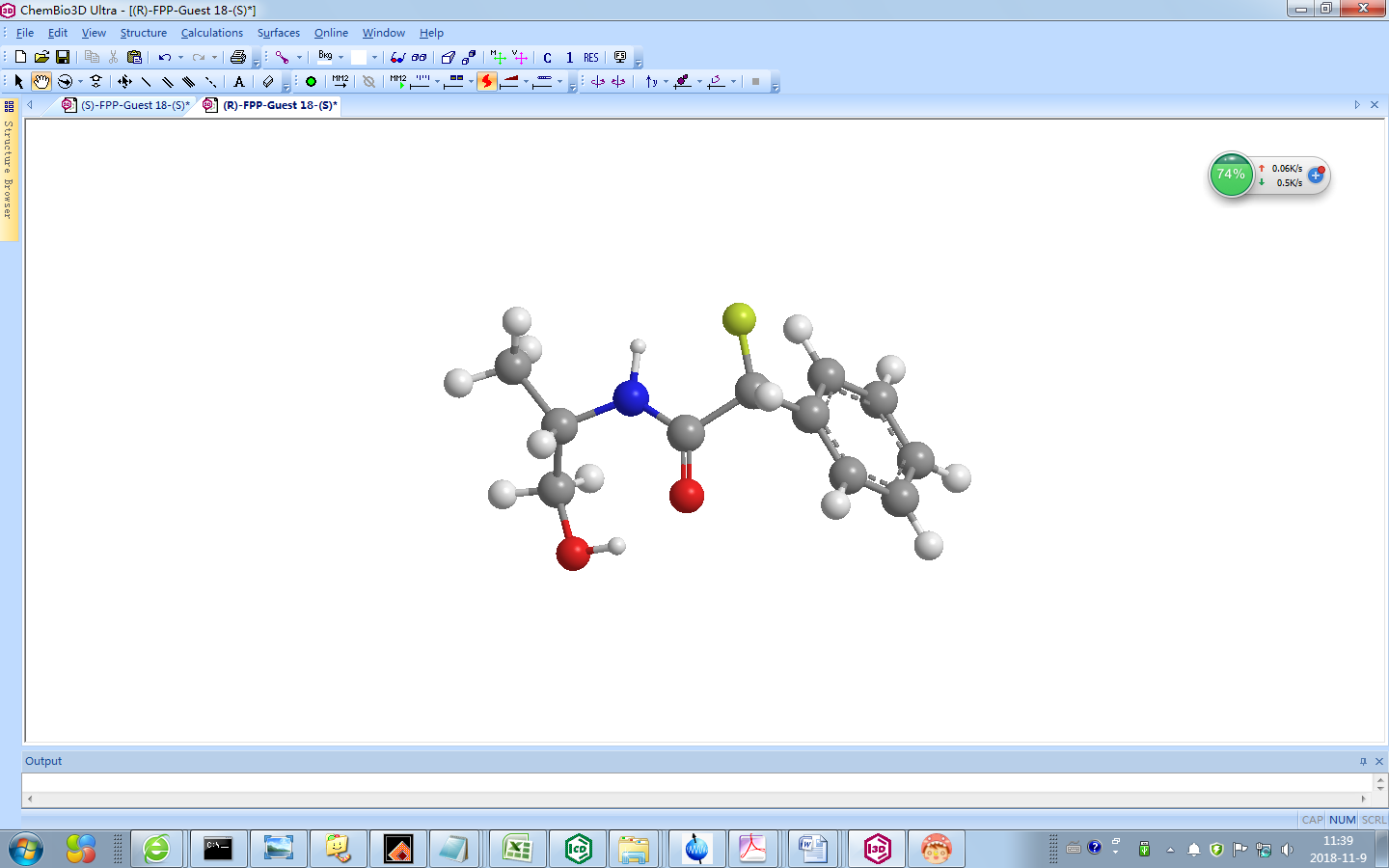
** δ_α-F_*^R^* _(calcd.)_ = -188.65 ppm

C 3.28214 -0.00649 -2.16759

C 3.9171 -0.8951 -1.30047

C 3.15504 -1.75678 -0.51452

C 1.7634 -1.72963 -0.58913

C 1.12734 -0.8414 -1.45828

C 1.89254 0.0191 -2.2502

C -0.3829 -0.78813 -1.53509

C -0.95764 0.41108 -0.76188

F -0.93417 -1.98332 -1.03872

N -1.69363 0.11173 0.31676

O -0.70652 1.54542 -1.17033

C -2.44226 1.07913 1.14757

C -1.53159 2.13756 1.8001

C -3.22327 0.28459 2.19424

O -1.21993 3.22684 0.95034

H 3.86881 0.66402 -2.78503

H 4.99923 -0.91791 -1.24118

H 3.64183 -2.45329 0.15866

H 1.1721 -2.40467 0.01613

H 1.4011 0.71252 -2.92181

H -0.71864 -0.68861 -2.5697

H -1.84248 -0.87131 0.49647

H -3.14084 1.61623 0.49661

H -2.0606 2.56014 2.65801

H -0.62678 1.63784 2.17791

H -3.88136 0.9484 2.75648

H -3.84288 -0.48249 1.72302

H -2.54592 -0.20446 2.90088

H -0.92386 2.84199 0.10654

**Ball-cylinder model for (*S*)-FPA-amide 18 and cartesian coordinates (Gaussian 09).**


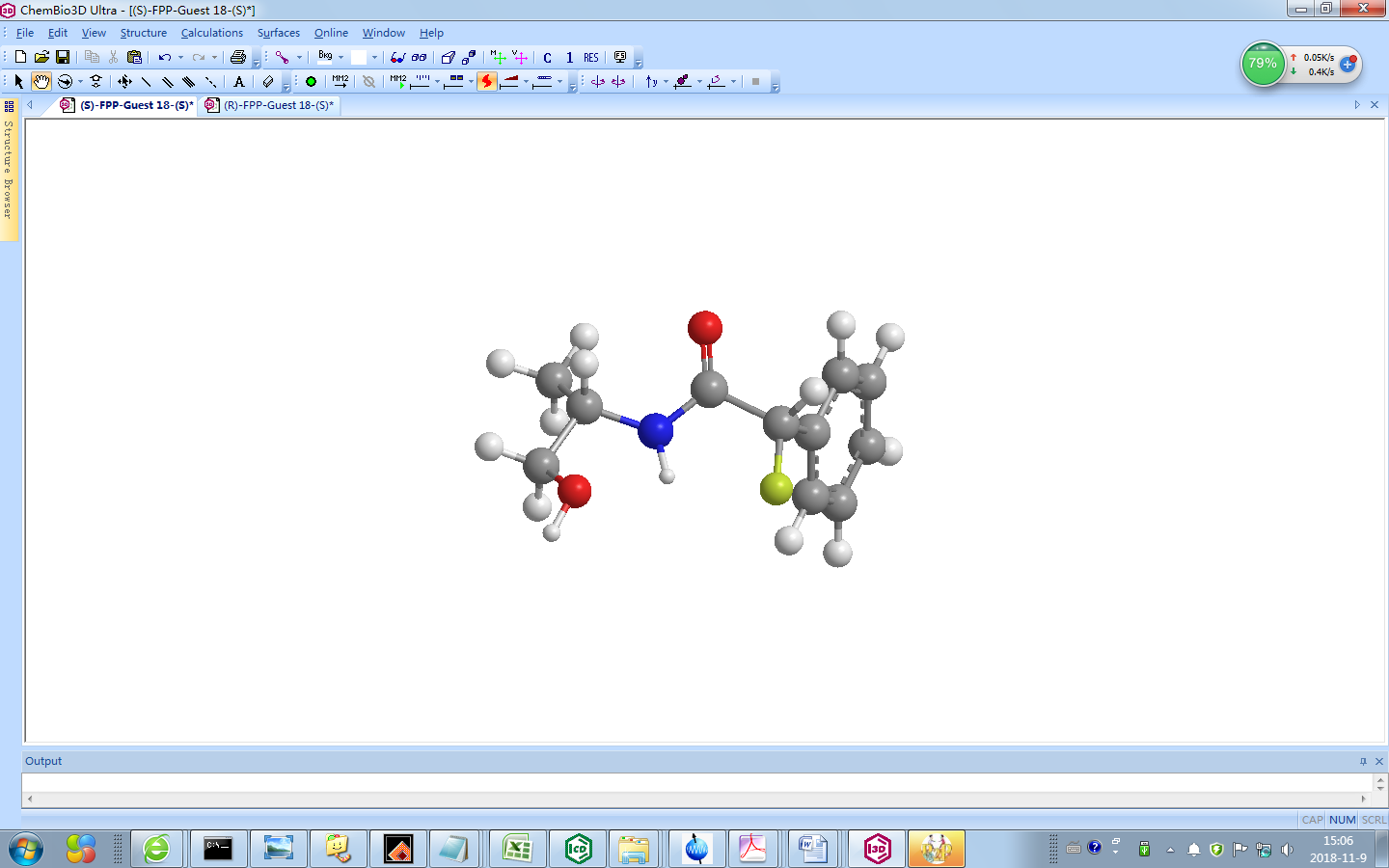
 δ_α-F_*^S^* _(calcd.)_ = -189.90 ppm

C 3.88118 -0.29635 -1.92742

C 4.04519 -1.67967 -1.9033

C 2.93537 -2.50372 -1.71875

C 1.66833 -1.94787 -1.56263

C 1.50308 -0.56004 -1.58294

C 2.61535 0.26398 -1.76444

C 0.12729 0.03581 -1.38405

C -0.3014 0.00833 0.09644

F 0.10067 1.35734 -1.86884

N -0.34403 1.21028 0.70277

O -0.54952 -1.06847 0.62365

C -0.86356 1.41162 2.06086

C -0.96493 2.91458 2.29968

C 0.01898 0.75119 3.12243

O -1.71547 3.48983 1.23002

H 4.73877 0.35067 -2.07376

H 5.03029 -2.11378 -2.03109

H 3.05444 -3.58112 -1.70232

H 0.80739 -2.58884 -1.41572

H 2.48772 1.33854 -1.78878

H -0.62513 -0.52241 -1.94508

H -0.34022 2.02415 0.10246

H -1.87095 0.98181 2.11214

H -1.4527 3.08436 3.26648

H 0.04473 3.34485 2.34078

H -0.39099 0.93481 4.11915

H 0.06445 -0.32434 2.95937

H 1.03469 1.15419 3.08341

H -1.66556 4.44763 1.30665

**Ball-cylinder model for (*R*)-FPA-amide 19 and cartesian coordinates (Gaussian 09).**

**
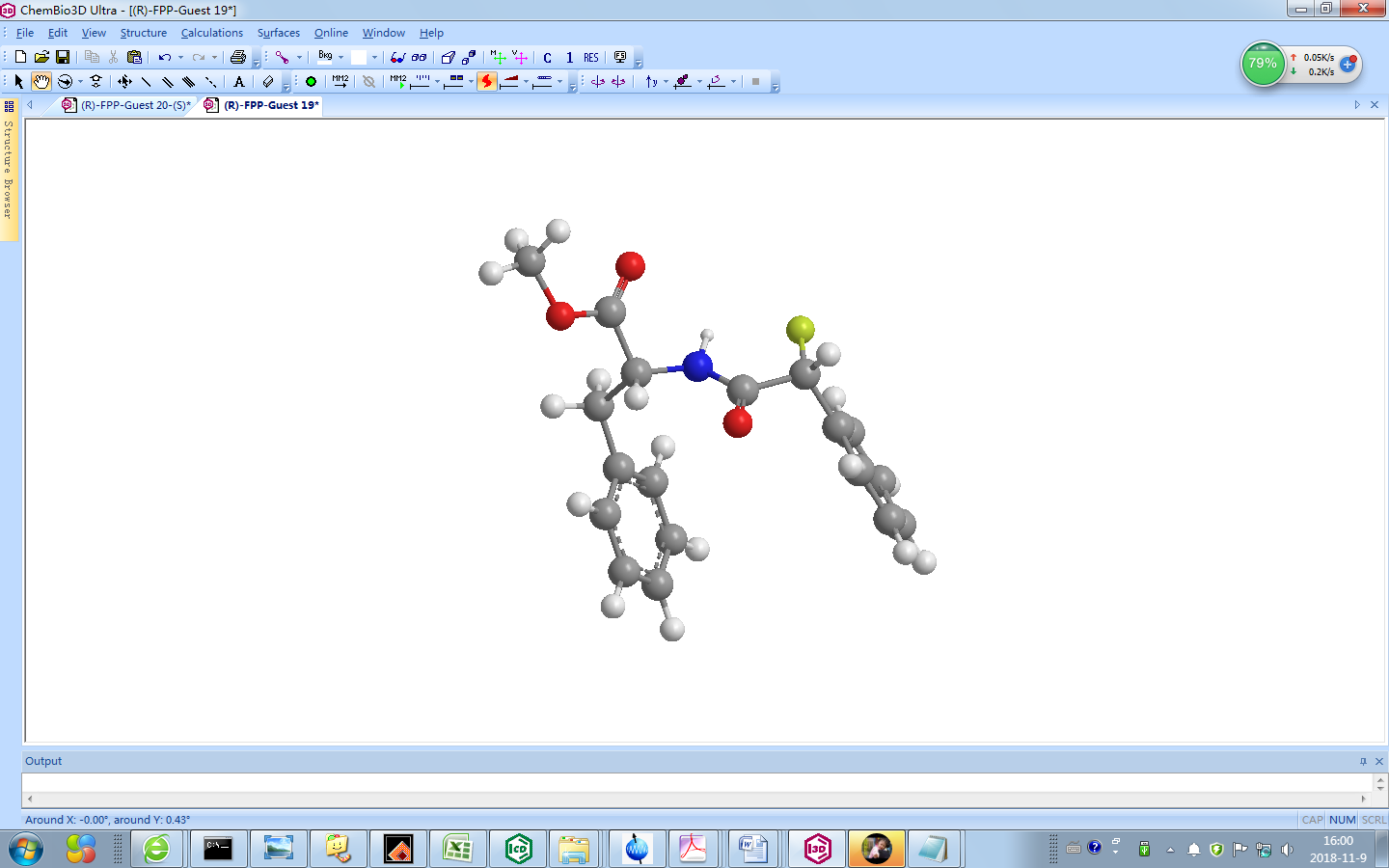
** δ_α-F_*^R^* _(calcd.)_ = -194.83 ppm

C 3.141110 -2.654470 2.668230

C 4.161130 -1.859600 3.190740

C 3.943150 -0.497750 3.384400

C 2.712990 0.071100 3.056570

C 1.691610 -0.724050 2.534660

C 1.909800 -2.091960 2.344210

C 0.350640 -0.121020 2.174320

C 0.115990 -0.066240 0.653390

F 0.237470 1.169780 2.721340

N 0.036060 1.178320 0.130930

O 0.016970 -1.105900 0.020020

C -0.297670 1.421780 -1.258260

C -1.571150 2.262450 -1.329770

C 0.840990 2.144340 -2.031690

O -2.119820 2.196780 -2.548350

C 2.127160 1.352610 -2.086650

C 2.270720 0.291670 -2.988510

C 3.449800 -0.446840 -3.039530

C 4.508350 -0.135000 -2.186700

C 4.378260 0.919610 -1.286260

C 3.195730 1.656800 -1.238330

O -2.004500 2.927060 -0.418990

C -3.304440 2.998510 -2.762680

H 3.302150 -3.715690 2.516160

H 5.117580 -2.300560 3.447660

H 4.729640 0.126680 3.793060

H 2.542000 1.127660 3.215910

H 1.121220 -2.708780 1.931550

H -0.465180 -0.712850 2.596430

H -0.010660 1.956920 0.773180

H -0.487770 0.452610 -1.719890

H 1.019050 3.114700 -1.558890

H 0.476460 2.338940 -3.043350

H 1.454200 0.043550 -3.659430

H 3.543520 -1.264080 -3.745980

H 5.427060 -0.709250 -2.225340

H 5.195810 1.169520 -0.619390

H 3.103860 2.479860 -0.537410

H -3.079470 4.053560 -2.605730

H -4.096390 2.687800 -2.081270

H -3.592640 2.816410 -3.794400

**Ball-cylinder model for (*S*)-FPA-amide 19 and cartesian coordinates (Gaussian 09).**


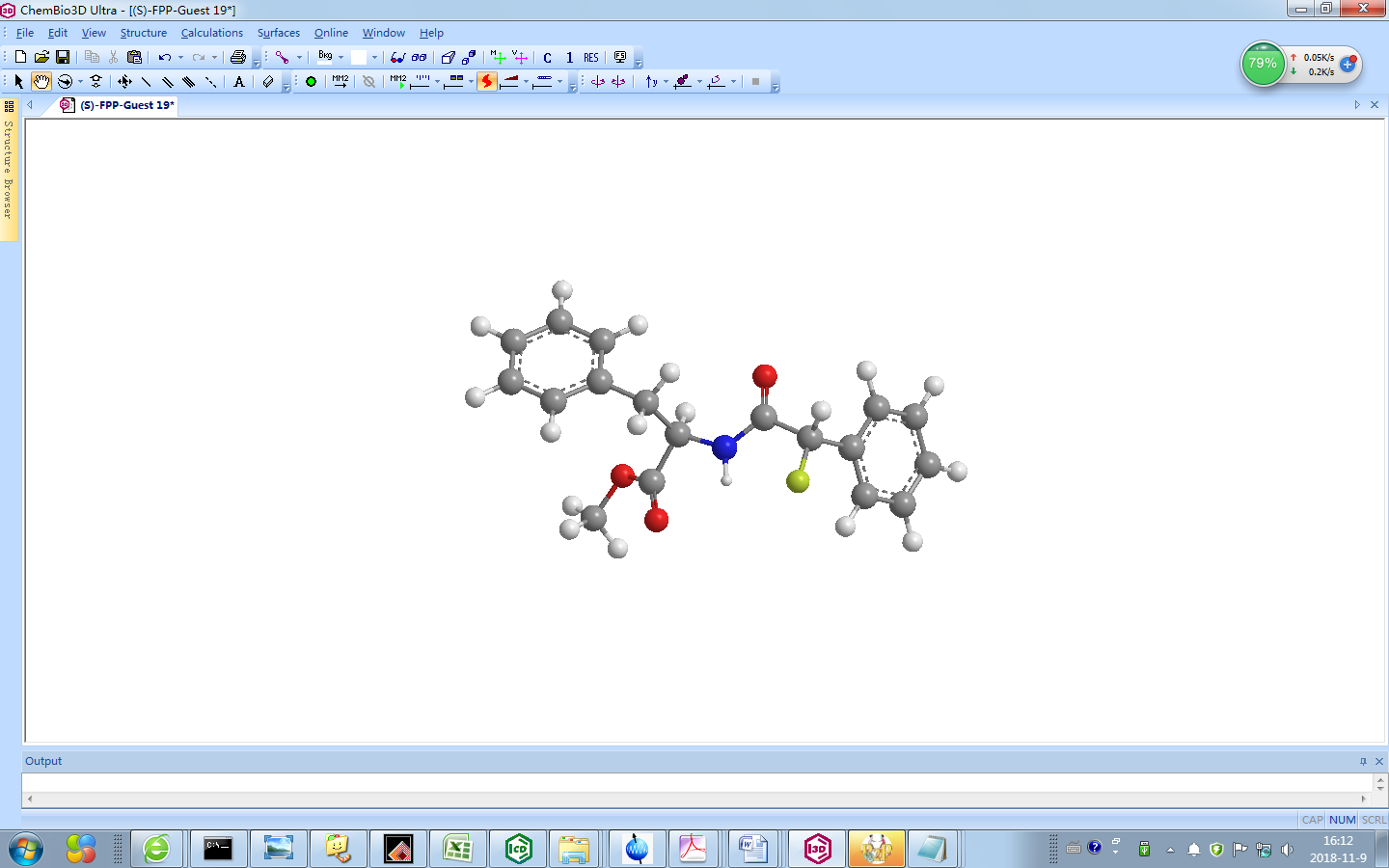
 δ_α-F_*^S^* _(calcd.)_ = -194.06 ppm

C 3.511330 -4.529053 0.705987

C 2.653520 -5.620613 0.571477

C 1.332800 -5.516903 1.001447

C 0.866840 -4.328163 1.561787

C 1.725280 -3.236013 1.698167

C 3.052080 -3.341893 1.269587

C 1.231590 -1.936373 2.297467

C 1.080570 -0.830483 1.237787

F 0.002400 -2.139773 2.948117

N -0.184560 -0.441943 0.988547

O 2.073550 -0.373973 0.686837

C -0.506130 0.589637 0.015647

C -1.931070 0.334147 -0.458593

C -0.365090 2.010157 0.642557

O -2.120710 0.761747 -1.706103

C -0.514890 3.153597 -0.334323

C 0.519960 3.462647 -1.225563

C 0.391100 4.512617 -2.131063

C -0.775940 5.276007 -2.157273

C -1.810020 4.982097 -1.270993

C -1.678400 3.928247 -0.367563

O -2.785070 -0.172753 0.233287

C -3.463220 0.639547 -2.231623

H 4.541530 -4.602783 0.376627

H 3.014320 -6.546153 0.137457

H 0.660440 -6.361673 0.902987

H -0.157590 -4.251703 1.902207

H 3.717150 -2.492753 1.367637

H 1.930330 -1.564403 3.050377

H -0.948430 -0.851373 1.509227

H 0.179010 0.503027 -0.827673

H 0.630080 2.038147 1.092677

H -1.093560 2.098537 1.452887

H 1.435590 2.880027 -1.204983

H 1.204070 4.739337 -2.811873

H -0.875230 6.096407 -2.859033

H -2.718160 5.574687 -1.278943

H -2.486440 3.709007 0.323107

H -3.763790 -0.407893 -2.252593

H -3.414780 1.047047 -3.237473

H -4.160460 1.209997 -1.618203

**Ball-cylinder model for (*R*)-FPA-amide 20 and cartesian coordinates (Gaussian 09).**


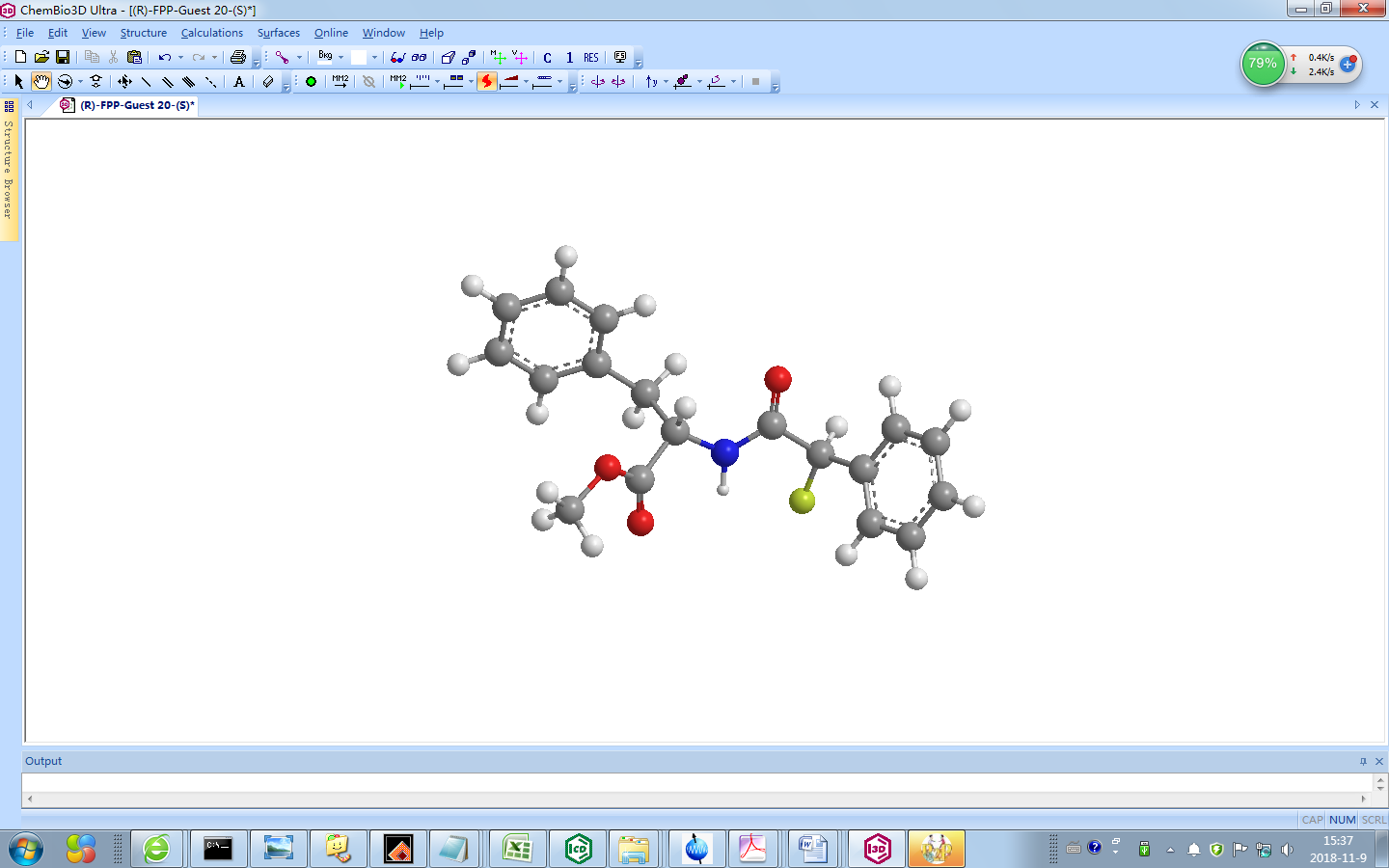
 δ_α-F_*^R^* _(calcd.)_ = -194.06 ppm

C 3.84246 -3.42556 -1.18541

C 2.98465 -4.51712 -1.0509

C 1.66393 -4.41341 -1.48087

C 1.19797 -3.22467 -2.04121

C 2.05641 -2.13252 -2.17759

C 3.38321 -2.2384 -1.74901

C 1.56272 -0.83288 -2.77689

C 1.4117 0.27301 -1.71721

F 0.33353 -1.03628 -3.42754

N 0.14657 0.66155 -1.46797

O 2.40468 0.72952 -1.16626

C -0.175 1.69313 -0.49507

C -1.59994 1.43764 -0.02083

C -0.03396 3.11365 -1.12198

O -1.78958 1.86524 1.22668

C -0.18376 4.25709 -0.1451

C 0.85109 4.56614 0.74614

C 0.72223 5.61611 1.65164

C -0.44481 6.3795 1.67785

C -1.47889 6.08559 0.79157

C -1.34727 5.03174 -0.11186

O -2.45394 0.93074 -0.71271

C -3.13209 1.74304 1.7522

H 4.87266 -3.49929 -0.85605

H 3.34545 -5.44266 -0.61688

H 0.99157 -5.25818 -1.38241

H 0.17354 -3.14821 -2.38163

H 4.04828 -1.38926 -1.84706

H 2.26146 -0.46091 -3.5298

H -0.6173 0.25212 -1.98865

H 0.51014 1.60652 0.34825

H 0.96121 3.14164 -1.5721

H -0.76243 3.20203 -1.93231

H 1.76672 3.98352 0.72556

H 1.5352 5.84283 2.33245

H -0.5441 7.1999 2.37961

H -2.38703 6.67818 0.79952

H -2.15531 4.8125 -0.80253

H -3.43266 0.6956 1.77317

H -3.08365 2.15054 2.75805

H -3.82933 2.31349 1.13878

**Ball-cylinder model for (*S*)-FPA-amide 20 and cartesian coordinates (Gaussian 09).**


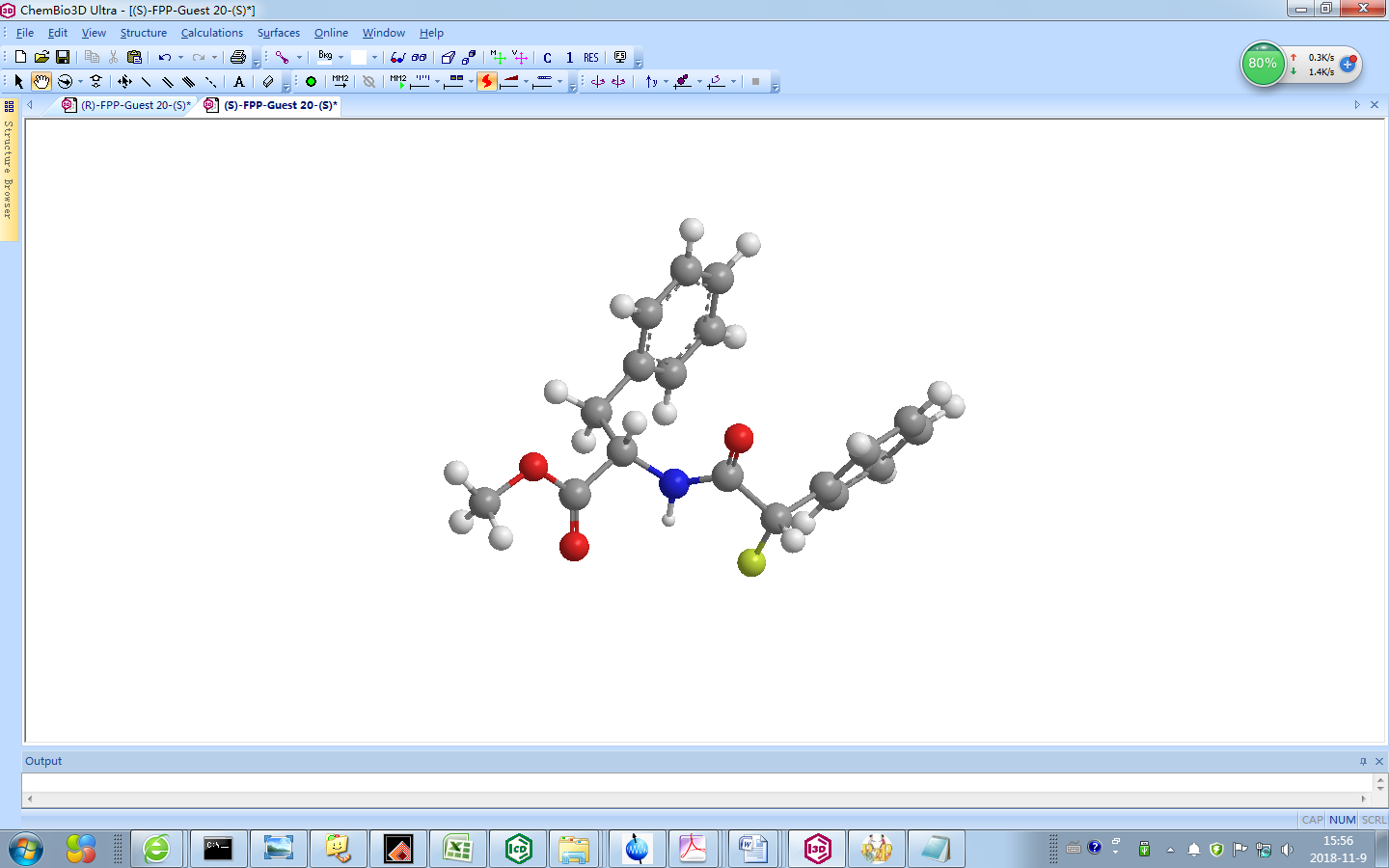
 δ_α-F_*^S^* _(calcd.)_ = -194.83 ppm

C 3.14111 -2.65447 -2.66823

C 4.16113 -1.8596 -3.19074

C 3.94315 -0.49775 -3.3844

C 2.71299 0.0711 -3.05657

C 1.69161 -0.72405 -2.53466

C 1.9098 -2.09196 -2.34421

C 0.35064 -0.12102 -2.17432

C 0.11599 -0.06624 -0.65339

F 0.23747 1.16978 -2.72134

N 0.03606 1.17832 -0.13093

O 0.01697 -1.1059 -0.02002

C -0.29767 1.42178 1.25826

C -1.57115 2.26245 1.32977

C 0.84099 2.14434 2.03169

O -2.11982 2.19678 2.54835

C 2.12716 1.35261 2.08665

C 2.27072 0.29167 2.98851

C 3.4498 -0.44684 3.03953

C 4.50835 -0.135 2.1867

C 4.37826 0.91961 1.28626

C 3.19573 1.6568 1.23833

O -2.0045 2.92706 0.41899

C -3.30444 2.99851 2.76268

H 3.30215 -3.71569 -2.51616

H 5.11758 -2.30056 -3.44766

H 4.72964 0.12668 -3.79306

H 2.542 1.12766 -3.21591

H 1.12122 -2.70878 -1.93155

H -0.46518 -0.71285 -2.59643

H -0.01066 1.95692 -0.77318

H -0.48777 0.45261 1.71989

H 1.01905 3.1147 1.55889

H 0.47646 2.33894 3.04335

H 1.4542 0.04355 3.65943

H 3.54352 -1.26408 3.74598

H 5.42706 -0.70925 2.22534

H 5.19581 1.16952 0.61939

H 3.10386 2.47986 0.53741

H -3.07947 4.05356 2.60573

H -4.09639 2.6878 2.08127

H -3.59264 2.81641 3.7944

**Ball-cylinder model for (*R*)-FPA-amide 21 and cartesian coordinates (Gaussian 09).**

**
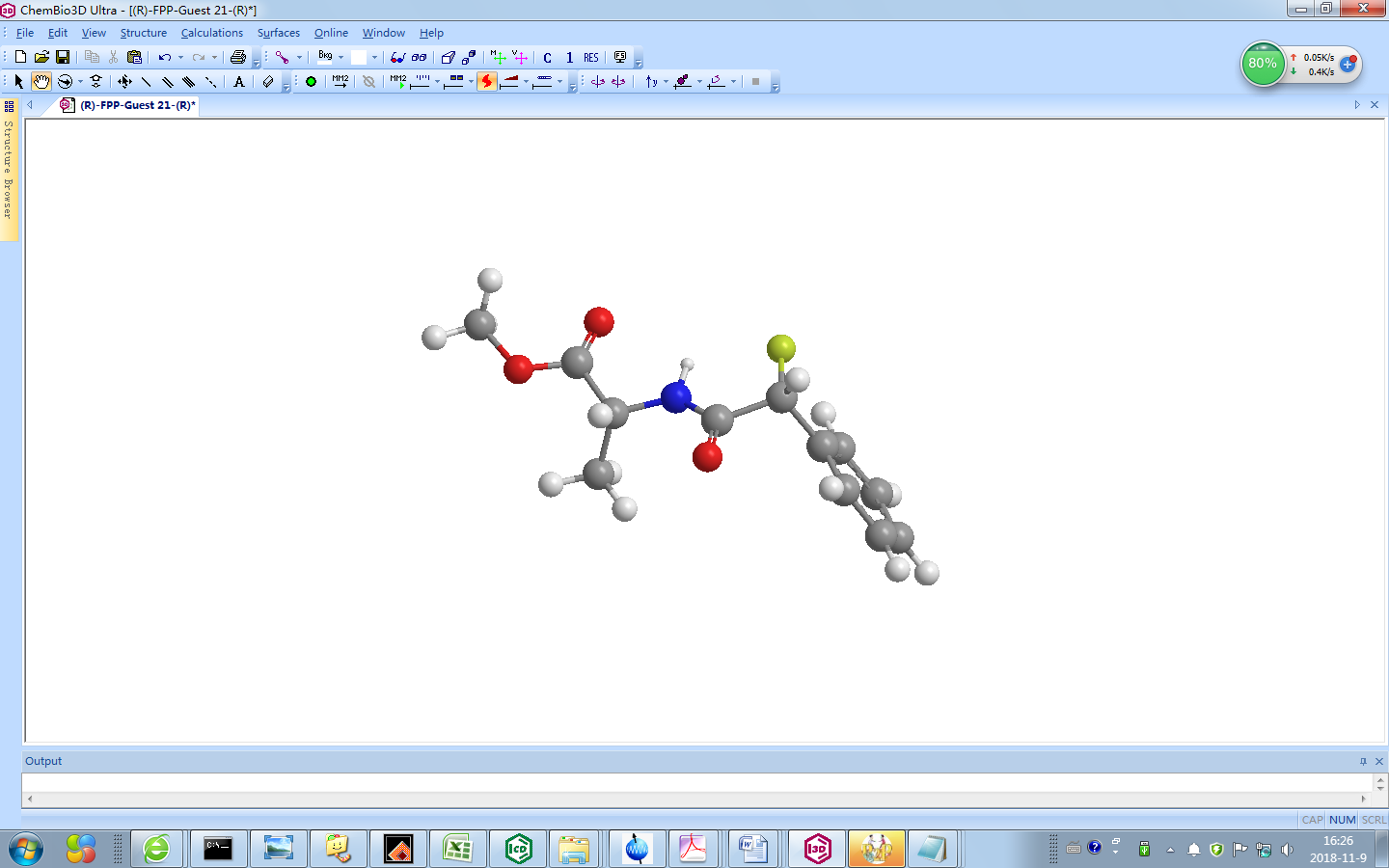
** δ_α-F_*^R^* _(calcd.)_ = -191.77 ppm

C 4.37214 -2.12705 -0.9258

C 4.0985 -3.32693 -1.58183

C 2.88319 -3.49058 -2.24294

C 1.94142 -2.4628 -2.24718

C 2.21561 -1.26051 -1.59279

C 3.4369 -1.09572 -0.93323

C 1.1961 -0.14288 -1.57386

C 0.4676 -0.05213 -0.221

F 0.26085 -0.3247 -2.60784

N -0.83942 -0.37208 -0.24829

O 1.08624 0.28561 0.7798

C -1.68434 -0.29827 0.93308

C -3.12341 -0.14042 0.45762

C -1.52903 -1.5276 1.8464

O -3.94093 0.12573 1.479

O -3.48039 -0.25895 -0.69184

C -5.34443 0.26579 1.15813

H 5.31723 -1.99087 -0.41252

H 4.82986 -4.12711 -1.5807

H 2.66471 -4.4191 -2.75821

H 1.00025 -2.58848 -2.76676

H 3.64905 -0.16562 -0.41997

H 1.67461 0.82411 -1.74444

H -1.29268 -0.55256 -1.13434

H -1.41418 0.59553 1.50212

H -1.80327 -2.4398 1.31145

H -2.16215 -1.42781 2.72884

H -0.48933 -1.60471 2.16499

H -5.72419 -0.65648 0.71856

H -5.83626 0.47156 2.10484

H -5.48961 1.09101 0.46102

**Ball-cylinder model for (*S*)-FPA-amide 21 and cartesian coordinates (Gaussian 09).**


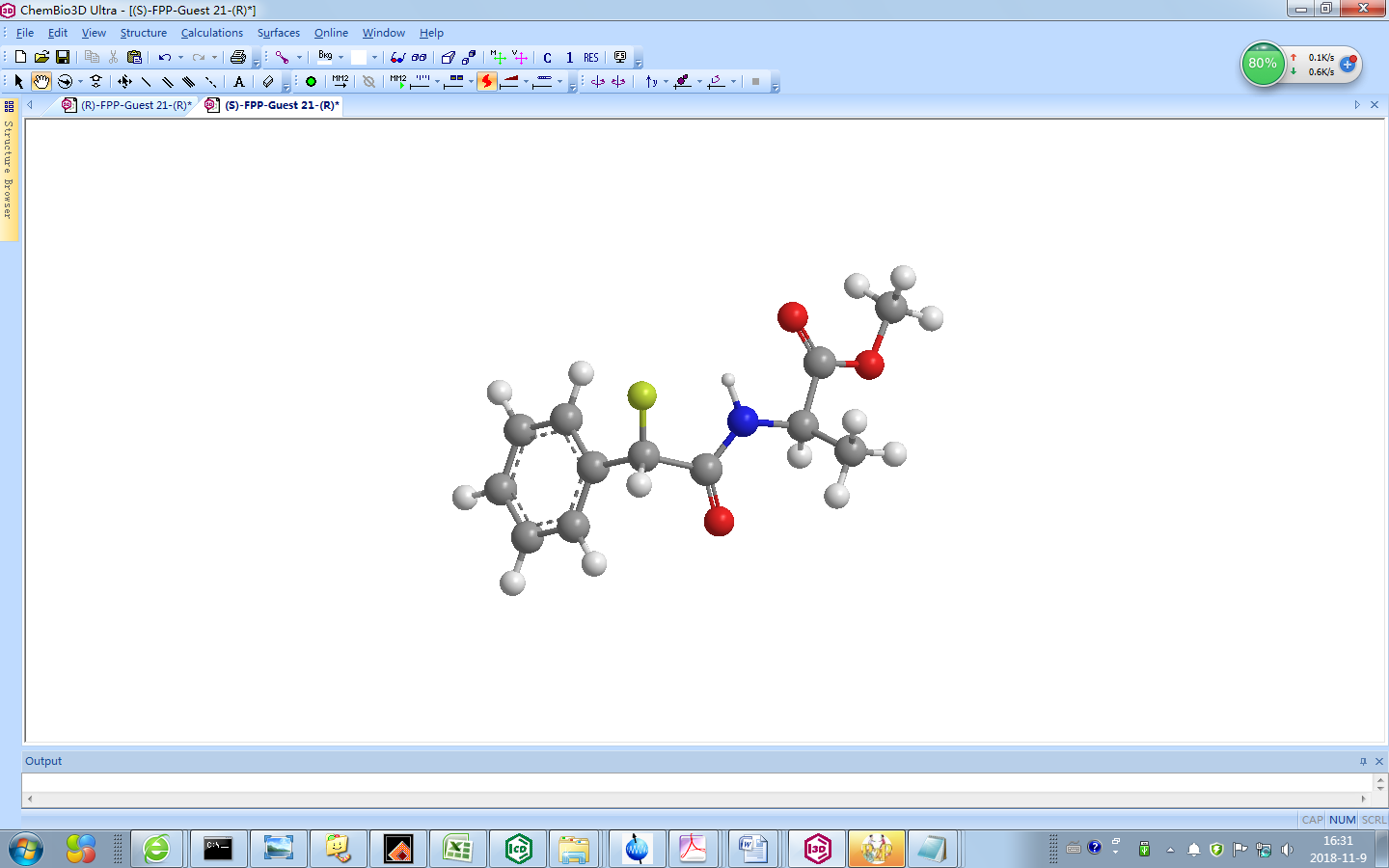
 δ_α-F_*^S^* _(calcd.)_ = -191.27 ppm

C 3.29639 0.17135 -2.86384

C 4.05847 -0.98701 -2.72646

C 3.45509 -2.16263 -2.28079

C 2.09644 -2.18061 -1.9762

C 1.33243 -1.01802 -2.1089

C 1.93773 0.15904 -2.55339

C -0.13553 -1.03683 -1.7462

C -0.35117 -0.95586 -0.22365

F -0.80245 0.0279 -2.37882

N -0.96729 0.15835 0.21171

O 0.03192 -1.87078 0.49346

C -1.20696 0.41916 1.62193

C -1.3852 1.92376 1.78481

C -2.42688 -0.34574 2.16691

O -1.38953 2.26894 3.0746

O -1.52563 2.69913 0.8674

C -1.59014 3.67175 3.36458

H 3.75785 1.08794 -3.21352

H 5.11501 -0.97566 -2.96888

H 4.04081 -3.06874 -2.17563

H 1.62968 -3.0943 -1.62818

H 1.3445 1.05745 -2.66635

H -0.61076 -1.95846 -2.08969

H -1.22923 0.87877 -0.44774

H -0.32263 0.11636 2.18864

H -2.56448 -0.13476 3.22814

H -2.26174 -1.41561 2.03853

H -3.33178 -0.05924 1.62595

H -2.55739 4.00051 2.98437

H -1.55711 3.74854 4.44787

H -0.79667 4.26624 2.91182

**Ball-cylinder model for (*R*)-FPA-amide 22 and cartesian coordinates (Gaussian 09).**

**
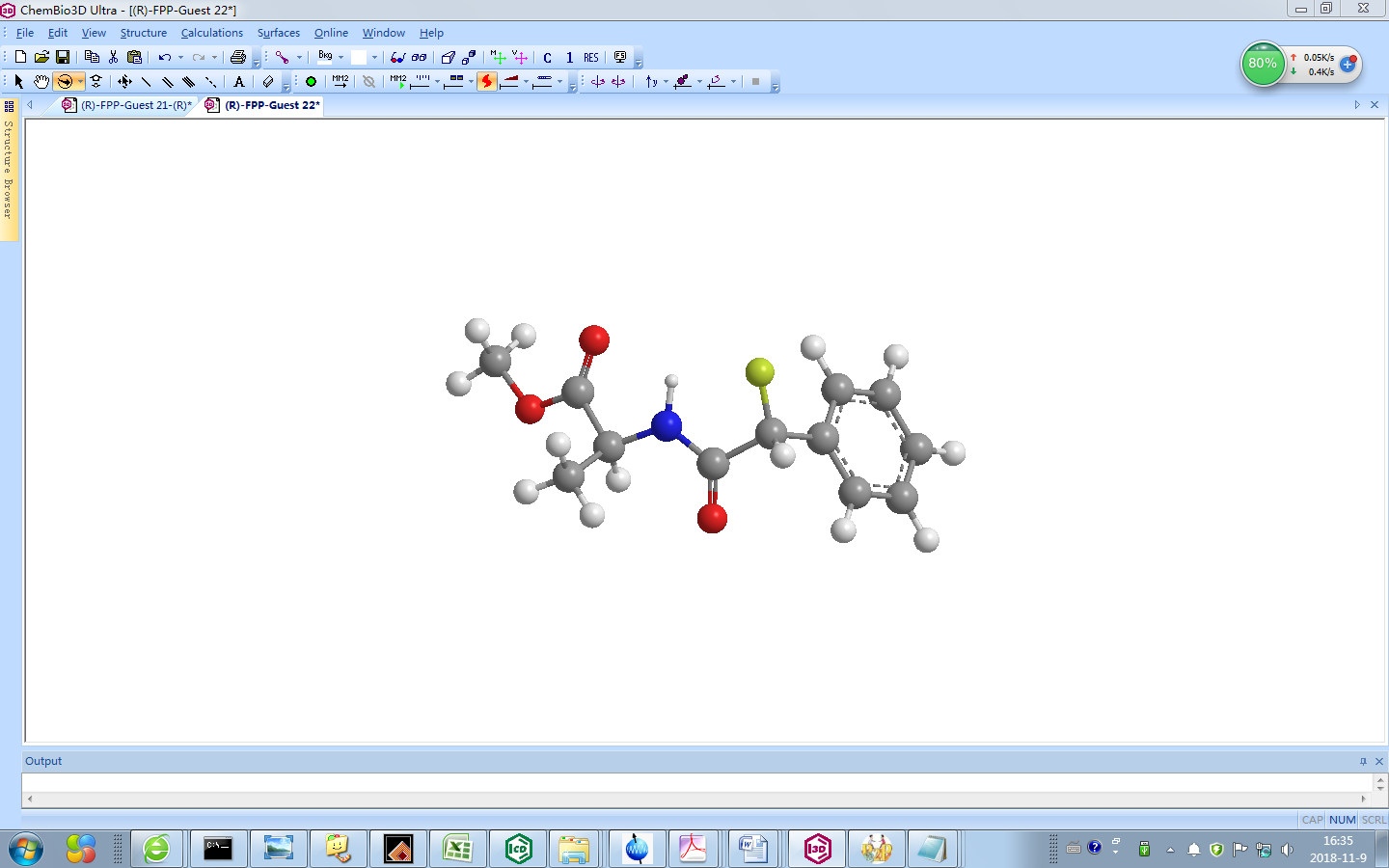
** δ_α-F_*^R^* _(calcd.)_ = -191.27 ppm

C 3.296390 0.171350 2.863840

C 4.058470 -0.987010 2.726460

C 3.455090 -2.162630 2.280790

C 2.096440 -2.180610 1.976200

C 1.332430 -1.018020 2.108900

C 1.937730 0.159040 2.553390

C -0.135530 -1.036830 1.746200

C -0.351170 -0.955860 0.223650

F -0.802450 0.027900 2.378820

N -0.967290 0.158350 -0.211710

O 0.031920 -1.870780 -0.493460

C -1.206960 0.419160 -1.621930

C -1.385200 1.923760 -1.784810

C -2.426880 -0.345740 -2.166910

O -1.389530 2.268940 -3.074600

O -1.525630 2.699130 -0.867400

C -1.590140 3.671750 -3.364580

H 3.757850 1.087940 3.213520

H 5.115010 -0.975660 2.968880

H 4.040810 -3.068740 2.175630

H 1.629680 -3.094300 1.628180

H 1.344500 1.057450 2.666350

H -0.610760 -1.958460 2.089690

H -1.229230 0.878770 0.447740

H -0.322630 0.116360 -2.188640

H -2.564480 -0.134760 -3.228140

H -2.261740 -1.415610 -2.038530

H -3.331780 -0.059240 -1.625950

H -2.557390 4.000510 -2.984370

H -1.557110 3.748540 -4.447870

H -0.796670 4.266240 -2.911820

**Ball-cylinder model for (*S*)-FPA-amide 22 and cartesian coordinates (Gaussian 09).**


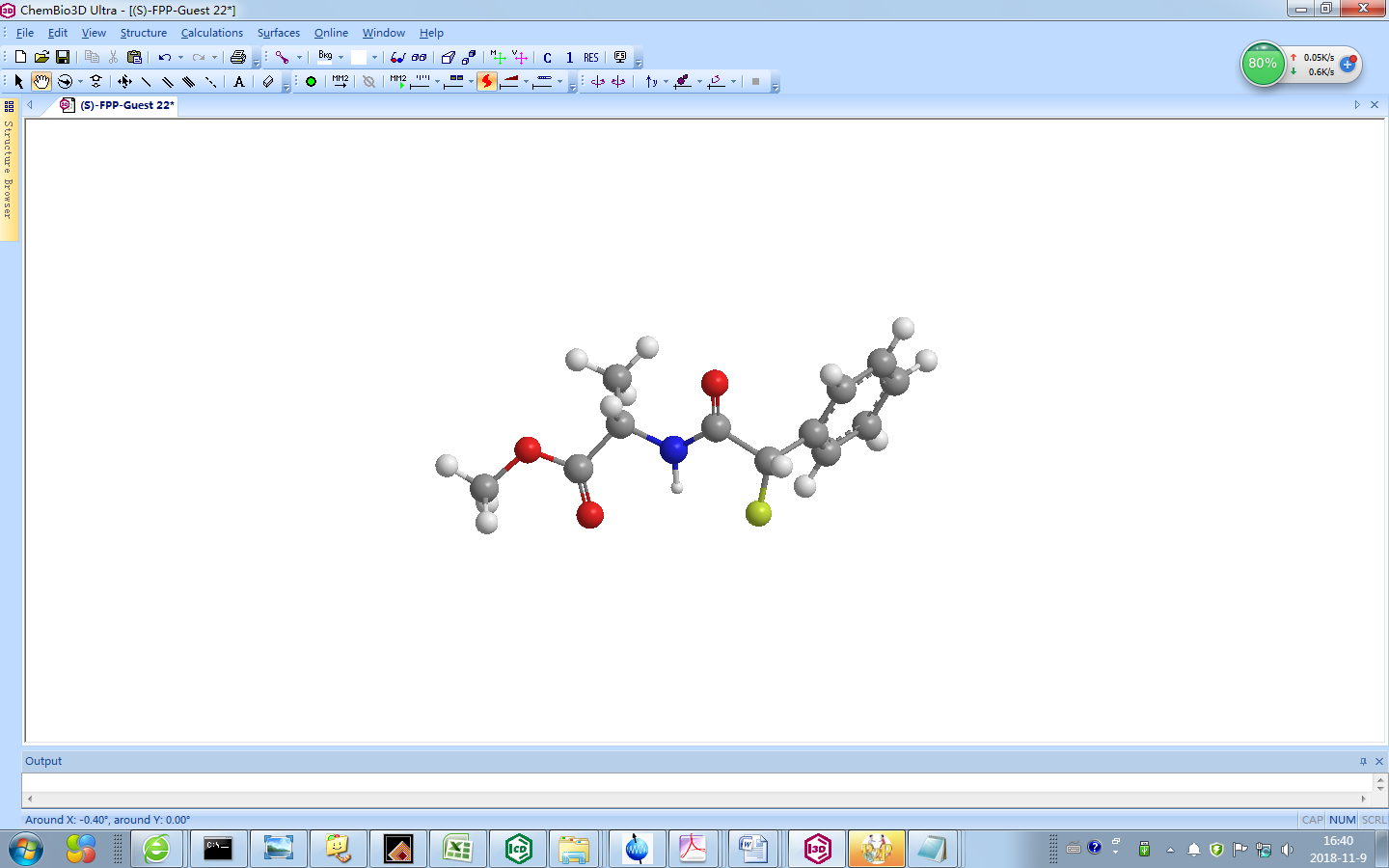
 δ_α-F_*^S^* _(calcd.)_ = -191.77 ppm

C 4.470829 -1.056282 0.666520

C 4.197189 -2.256162 1.322550

C 2.981879 -2.419812 1.983660

C 2.040109 -1.392032 1.987900

C 2.314299 -0.189742 1.333510

C 3.535589 -0.024952 0.673950

C 1.294789 0.927888 1.314580

C 0.566289 1.018638 -0.038280

F 0.359539 0.746068 2.348560

N -0.740731 0.698688 -0.010990

O 1.184929 1.356378 -1.039080

C -1.585651 0.772498 -1.192360

C -3.024721 0.930348 -0.716900

C -1.430341 -0.456832 -2.105680

O -3.842241 1.196498 -1.738280

O -3.381701 0.811818 0.432560

C -5.245741 1.336558 -1.417410

H 5.415919 -0.920102 0.153240

H 4.928549 -3.056342 1.321420

H 2.763399 -3.348332 2.498930

H 1.098939 -1.517712 2.507480

H 3.747739 0.905148 0.160690

H 1.773299 1.894878 1.485160

H -1.193991 0.518208 0.875060

H -1.315491 1.666298 -1.761400

H -1.704581 -1.369032 -1.570730

H -2.063461 -0.357042 -2.988120

H -0.390641 -0.533942 -2.424270

H -5.625501 0.414288 -0.977840

H -5.737571 1.542328 -2.364120

H -5.390921 2.161778 -0.720300

**Ball-cylinder model for (*R*)-FPP-(*R*)-amine 23 and cartesian coordinates (Gaussian 09).**

**
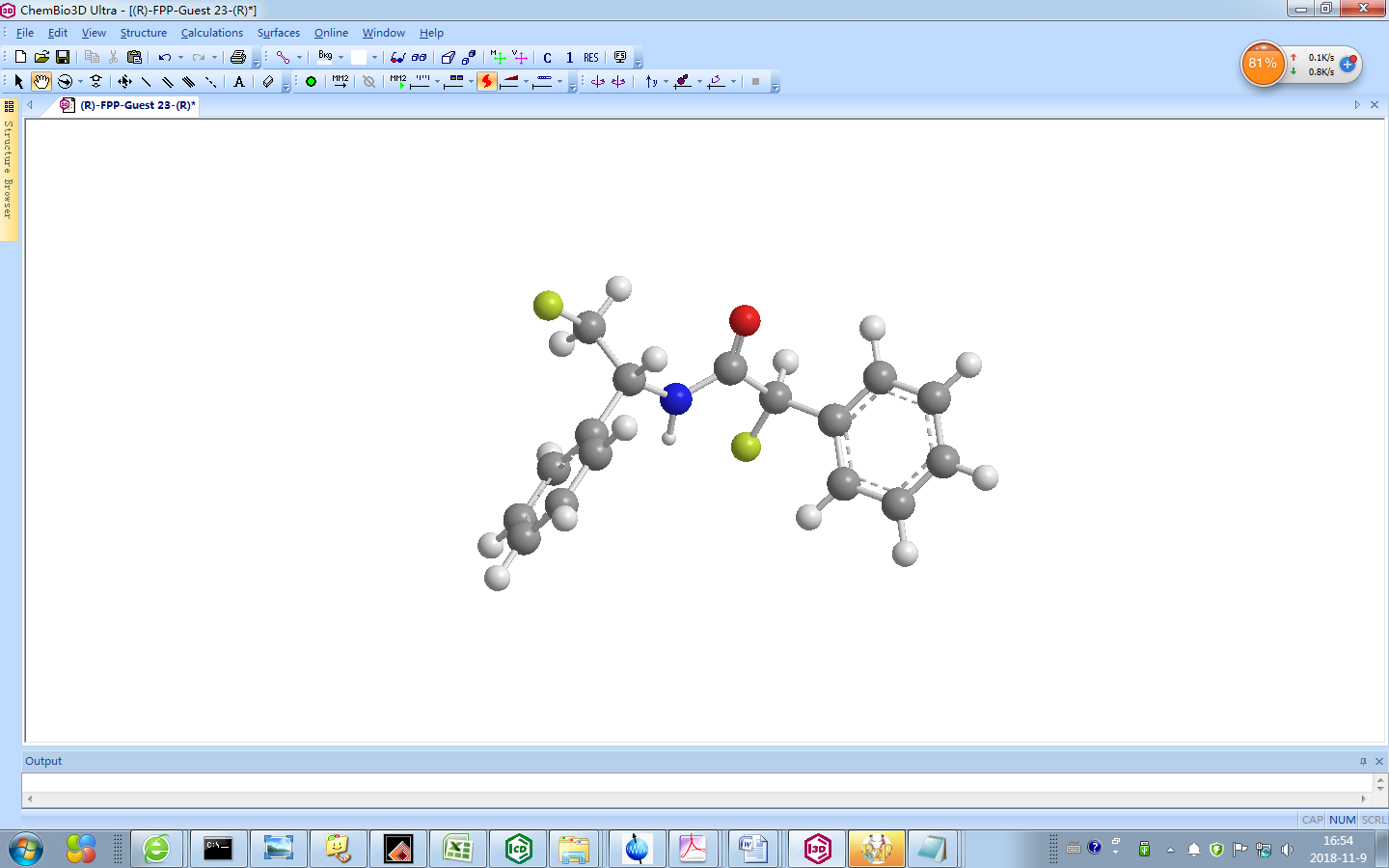
** δ_α-F_*^R^* _(calcd.)_ = -193.63 ppm

C 4.32136 -2.32897 -1.06888

C 3.75502 -2.98243 -2.16315

C 2.66391 -2.41487 -2.81731

C 2.13593 -1.20101 -2.38013

C 2.7038 -0.54552 -1.2863

C 3.80127 -1.11344 -0.63283

C 2.13063 0.76311 -0.78909

C 1.3039 0.57672 0.49655

F 1.33168 1.35256 -1.78605

N -0.01492 0.84032 0.38035

O 1.85439 0.21103 1.52516

C -0.9563 0.63475 1.48713

C -0.9214 1.85537 2.41358

C -2.34172 0.31816 0.9563

F -1.74105 1.6091 3.52101

C -2.91743 -0.92931 1.20778

C -4.18612 -1.2386 0.72069

C -4.89283 -0.30255 -0.03103

C -4.32642 0.94495 -0.28966

C -3.06107 1.25407 0.20336

H 5.17247 -2.76298 -0.55667

H 4.16449 -3.92611 -2.50505

H 2.22033 -2.91542 -3.67054

H 1.29247 -0.75762 -2.89342

H 4.23872 -0.60924 0.22025

H 2.92603 1.47568 -0.55939

H -0.36688 1.1249 -0.52183

H -0.58942 -0.21625 2.06478

H 0.09463 2.01771 2.77633

H -1.28939 2.75403 1.91345

H -2.36992 -1.66153 1.7912

H -4.62004 -2.21023 0.92761

H -5.87931 -0.54155 -0.41152

H -4.87148 1.67967 -0.87127

H -2.64174 2.23448 0.001

**Ball-cylinder model for (*S*)-FPP-(*R*)-amine 23 and cartesian coordinates (Gaussian 09).**


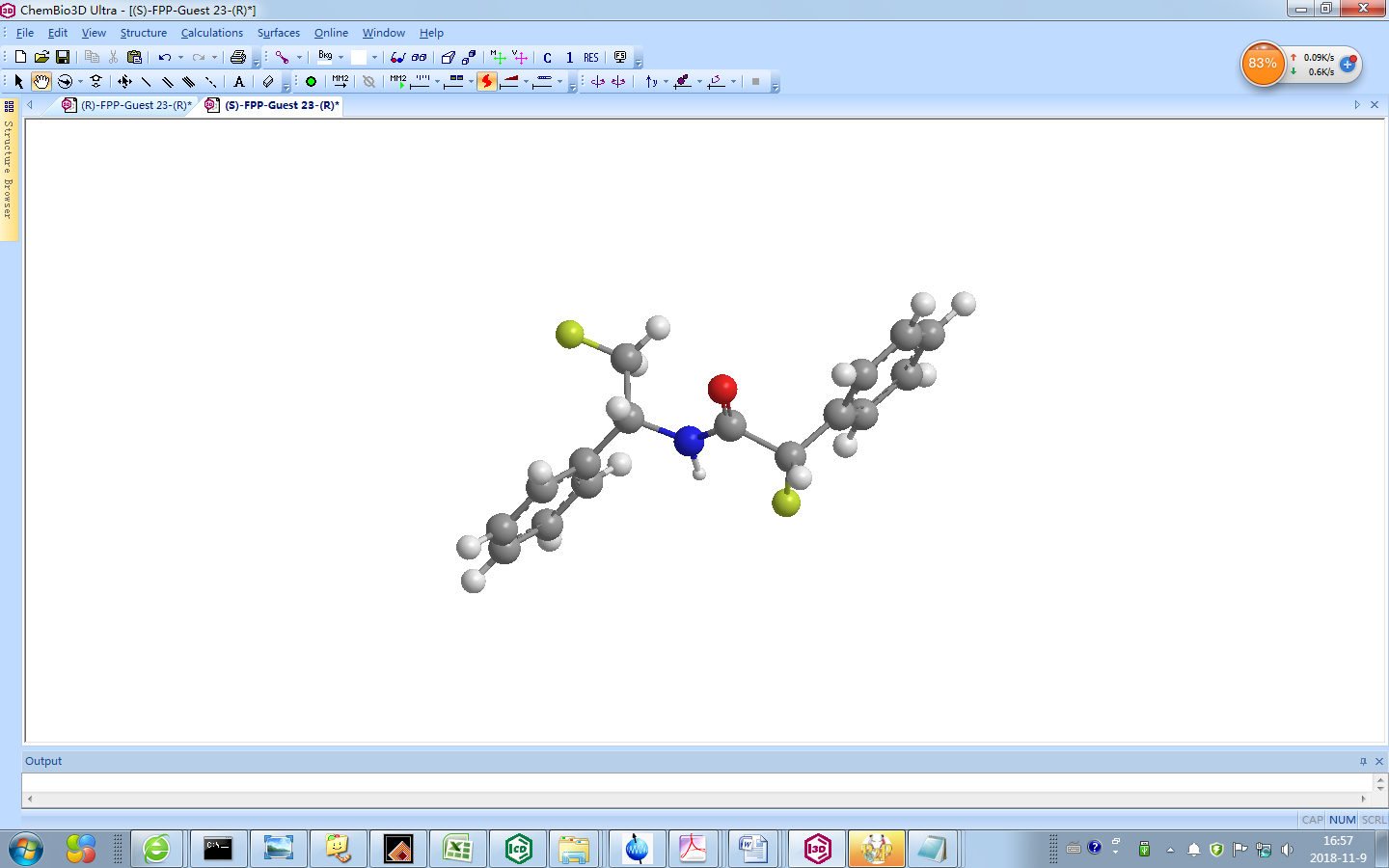
 δ_α-F_*^S^* _(calcd.)_ = -192.95 ppm

C 4.18912 0.16882 -2.66523

C 5.20114 -0.46067 -1.94425

C 4.87278 -1.41724 -0.98384

C 3.54045 -1.74433 -0.74706

C 2.52401 -1.10925 -1.46694

C 2.85323 -0.15112 -2.42715

C 1.07736 -1.45603 -1.18928

C 0.58402 -0.85957 0.14126

F 0.26195 -1.00481 -2.24315

N -0.36351 0.09627 0.03149

O 1.05297 -1.25936 1.19718

C -0.99382 0.70932 1.20642

C -0.13511 1.88862 1.67617

C -2.43198 1.08801 0.90822

F -0.67932 2.40618 2.85709

C -3.47533 0.4595 1.59174

C -4.80254 0.78969 1.32334

C -5.10202 1.75128 0.3609

C -4.06809 2.38365 -0.32836

C -2.74238 2.05619 -0.05421

H 4.43656 0.91024 -3.4165

H 6.23942 -0.21157 -2.1313

H 5.65492 -1.91444 -0.42155

H 3.28683 -2.48401 0.00252

H 2.06843 0.33253 -2.99422

H 0.94027 -2.53769 -1.1235

H -0.73136 0.29672 -0.887

H -0.97613 -0.0381 2.00232

H 0.87847 1.5477 1.89305

H -0.1094 2.69086 0.93525

H -3.24655 -0.29039 2.34109

H -5.60058 0.29521 1.8654

H -6.13365 2.00904 0.15033

H -4.29297 3.13553 -1.07621

H -1.95079 2.56667 -0.59335

**Ball-cylinder model for (*R*)-FPP-(*S*)-amine 23 and cartesian coordinates (Gaussian 09).**


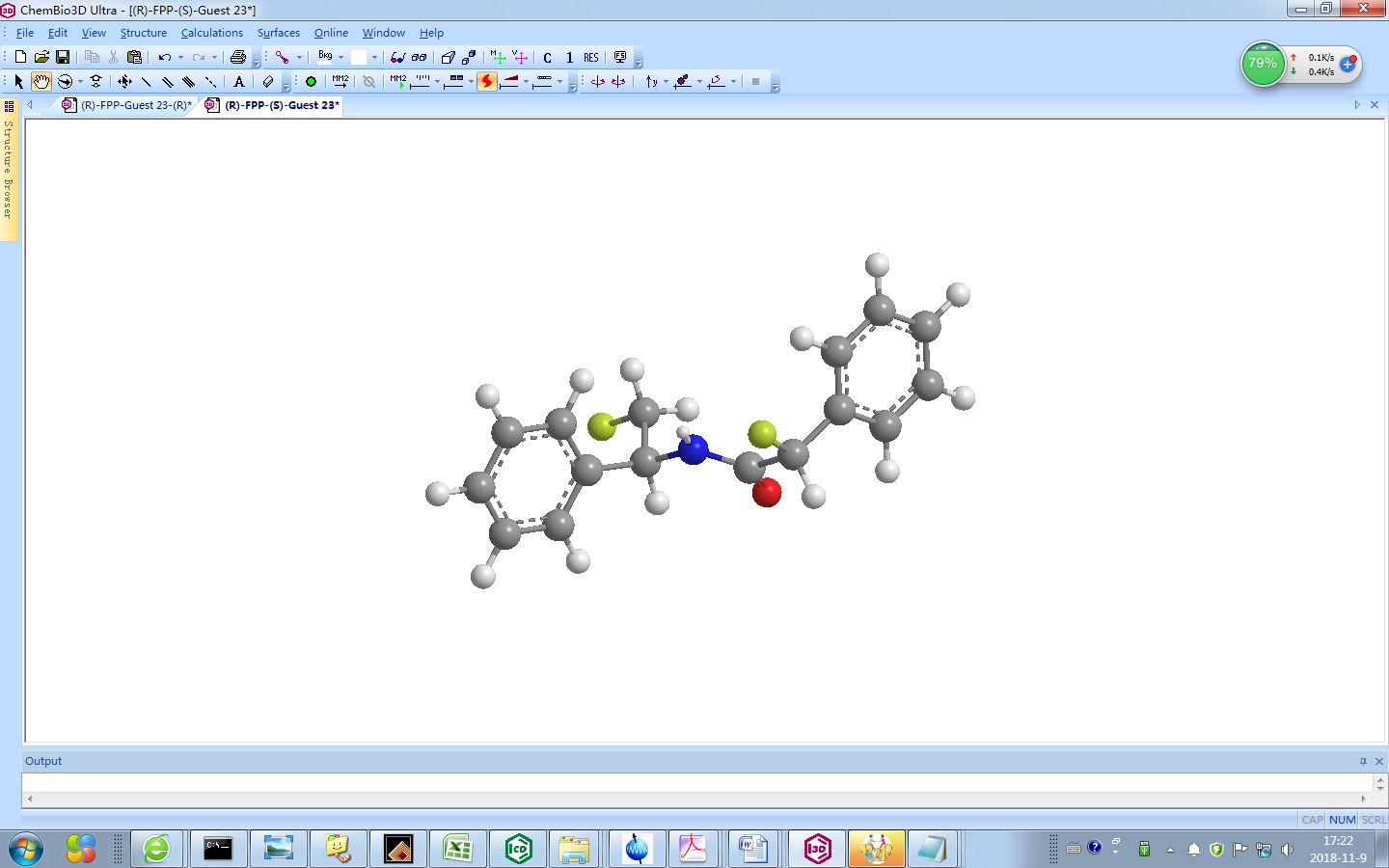
 δ_α-F_*^R^* _(calcd.)_ = -192.95 ppm

C 4.189120 0.168820 2.665230

C 5.201140 -0.460670 1.944250

C 4.872780 -1.417240 0.983840

C 3.540450 -1.744330 0.747060

C 2.524010 -1.109250 1.466940

C 2.853230 -0.151120 2.427150

C 1.077360 -1.456030 1.189280

C 0.584020 -0.859570 -0.141260

F 0.261950 -1.004810 2.243150

N -0.363510 0.096270 -0.031490

O 1.052970 -1.259360 -1.197180

C -0.993820 0.709320 -1.206420

C -0.135110 1.888620 -1.676170

C -2.431980 1.088010 -0.908220

F -0.679320 2.406180 -2.857090

C -3.475330 0.459500 -1.591740

C -4.802540 0.789690 -1.323340

C -5.102020 1.751280 -0.360900

C -4.068090 2.383650 0.328360

C -2.742380 2.056190 0.054210

H 4.436560 0.910240 3.416500

H 6.239420 -0.211570 2.131300

H 5.654920 -1.914440 0.421550

H 3.286830 -2.484010 -0.002520

H 2.068430 0.332530 2.994220

H 0.940270 -2.537690 1.123500

H -0.731360 0.296720 0.887000

H -0.976130 -0.038100 -2.002320

H 0.878470 1.547700 -1.893050

H -0.109400 2.690860 -0.935250

H -3.246550 -0.290390 -2.341090

H -5.600580 0.295210 -1.865400

H -6.133650 2.009040 -0.150330

H -4.292970 3.135530 1.076210

H -1.950790 2.566670 0.593350

**Ball-cylinder model for (*S*)-FPP-(*S*)-amine 23 and cartesian coordinates (Gaussian 09).**


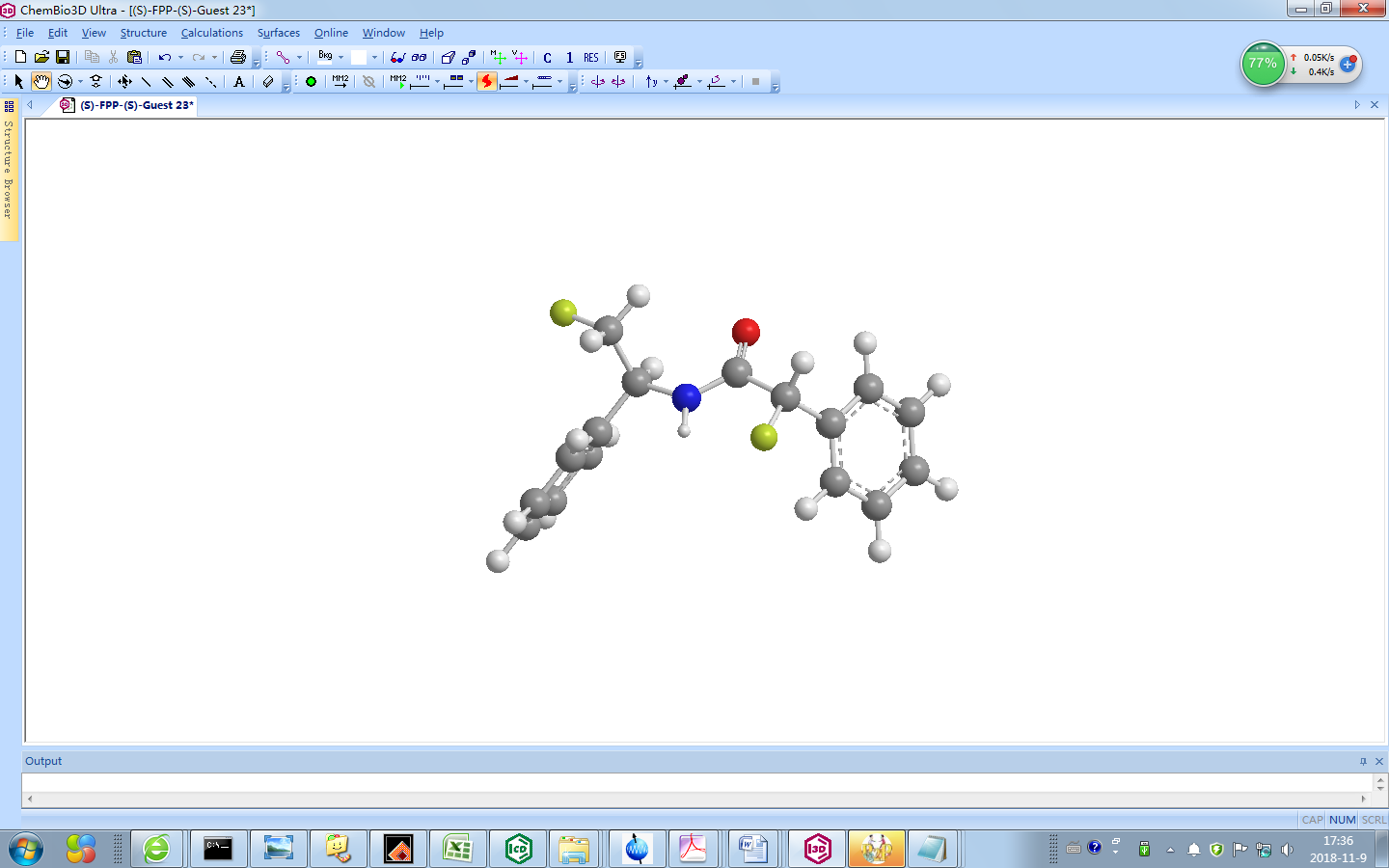
 δ_α-F_*^S^* _(calcd.)_ = -193.63 ppm

C 4.374972 -2.128655 0.993806

C 3.808632 -2.782115 2.088076

C 2.717522 -2.214555 2.742236

C 2.189542 -1.000695 2.305056

C 2.757412 -0.345205 1.211226

C 3.854882 -0.913125 0.557756

C 2.184242 0.963425 0.714016

C 1.357512 0.777035 -0.571624

F 1.385292 1.552875 1.710976

N 0.038692 1.040635 -0.455424

O 1.908002 0.411345 -1.600234

C -0.902688 0.835065 -1.562204

C -0.867788 2.055685 -2.488654

C -2.288108 0.518475 -1.031374

F -1.687438 1.809415 -3.596084

C -2.863818 -0.728995 -1.282854

C -4.132508 -1.038285 -0.795764

C -4.839218 -0.102235 -0.044044

C -4.272808 1.145265 0.214586

C -3.007458 1.454385 -0.278434

H 5.226082 -2.562665 0.481596

H 4.218102 -3.725795 2.429976

H 2.273942 -2.715105 3.595466

H 1.346082 -0.557305 2.818346

H 4.292332 -0.408925 -0.295324

H 2.979642 1.675995 0.484316

H -0.313268 1.325215 0.446756

H -0.535808 -0.015935 -2.139854

H 0.148242 2.218025 -2.851404

H -1.235778 2.954345 -1.988524

H -2.316308 -1.461215 -1.866274

H -4.566428 -2.009915 -1.002684

H -5.825698 -0.341235 0.336446

H -4.817868 1.879985 0.796196

H -2.588128 2.434795 -0.076074

# 3. ^19^F NMR calculation for all conformers according to Boltzmann equations based on free energies data by frequency calculation.

| Molecules | Δδ_α-F_*^R,S^*_Calcd_ (ppm)  (most stable conformer) ^[a]^ | Δδ_α-F_*^R,S^*_Calcd_ (ppm)  (boltzmann equations statistic) ^[a]^ |
| --- | --- | --- |
|  | - 0.6626 | -0. 6756 |
|  | -0.4975 | -0.5286 |

^[a]^ Δδ_α-F_*^R,S^* = δ_α-F_ _(_*_R_*_)-FPA-amide_ - δ_α-F_ _(_*_S_*_)-FPA-amide._
